# Improved protocols to accelerate the assembly of DNA barcode reference libraries for freshwater zooplankton

**DOI:** 10.1002/ece3.3742

**Published:** 2018-02-15

**Authors:** Manuel Elías‐Gutiérrez, Martha Valdez‐Moreno, Janet Topan, Monica R. Young, José Angel Cohuo‐Colli

**Affiliations:** ^1^ El Colegio de la Frontera Sur Chetumal Quintana Roo Mexico; ^2^ Centre for Biodiversity Genomics University of Guelph Guelph ON Canada; ^3^ Instituto Tecnológico de Chetumal Chetumal Quintana Roo Mexico

**Keywords:** Chironomidae, Cladocera, Copepoda, ichthyoplankton, Rotifera, water mites

## Abstract

Currently, freshwater zooplankton sampling and identification methodologies have remained virtually unchanged since they were first established in the beginning of the XX century. One major contributing factor to this slow progress is the limited success of modern genetic methodologies, such as DNA barcoding, in several of the main groups. This study demonstrates improved protocols which enable the rapid assessment of most animal taxa inhabiting any freshwater system by combining the use of light traps, careful fixation at low temperatures using ethanol, and zooplankton‐specific primers. We DNA‐barcoded 2,136 specimens from a diverse array of taxonomic assemblages (rotifers, mollusks, mites, crustaceans, insects, and fishes) from several Canadian and Mexican lakes with an average sequence success rate of 85.3%. In total, 325 Barcode Index Numbers (BINs) were detected with only three BINs (two cladocerans and one copepod) shared between Canada and Mexico, suggesting a much narrower distribution range of freshwater zooplankton than previously thought. This study is the first to broadly explore the metazoan biodiversity of freshwater systems with DNA barcodes to construct a reference library that represents the first step for future programs which aim to monitor ecosystem health, track invasive species, or improve knowledge of the ecology and distribution of freshwater zooplankton.

## 1 INTRODUCTION

The study of freshwater zooplankton has been challenging because of various impediments to taxonomy (Elías‐Gutiérrez, Suárez‐Morales, et al., 2008) which are expressed in all major taxa, restricting morphological identifications to a small group of experts. As a result, many studies on ecology, ecotoxicology, and distribution, among others, are based on incorrect species identifications making results impossible to verify or reproduce (Montoliu Elena, Elías‐Gutiérrez, Miracle Sole, & Korinek, 2015). However, DNA barcoding is an excellent alternative to traditional specimen identification because it can provide operational taxonomy based on molecular divergences of a standardized region of the cytochrome *c* oxidase subunit I (COI) gene in animals. DNA barcoding has been successfully implemented in freshwater and marine zooplankton studies (Bucklin et al., 2010; Elías‐Gutiérrez, Martínez‐Jerónimo, Ivanova, & Valdez‐Moreno, 2008), and its use has not yet been widely adopted in the community. This is reflected in the very limited barcode coverage for zooplankton in the Barcode of Life Datasystem (BOLD, www.boldsystems.org) in comparison with terrestrial arthropods (50K vs. 4M barcode records, respectively).

The main barrier in using DNA barcodes for freshwater zooplankton research is the historically low amplification success rates of the target gene region (COI) in the major zooplankton taxa. For example, copepods are one of the most abundant (Mauchline, Blaxter, Southward, & Tyler, 1998) and diverse groups on our planet, yet only 14 DNA barcode studies have been published for this group. Low barcode success rates have also been reported for cladocerans and rotifers, and most barcode studies on these groups are taxonomically limited in scope (Elías‐Gutiérrez, Martínez‐Jerónimo, et al., 2008; Hwang, Dahms, Park, & Lee, 2013; Jeffery, Elías‐Gutiérrez, & Adamowicz, 2011; Proios et al., 2014). Although DNA barcodes successfully discriminate other taxa of freshwater zooplankton such as ostracods (Cohuo‐Durán, Elías‐Gutiérrez, & Karanovic, 2013; Martens, Halse, & Schoen, 2015), no broad barcoding studies have been undertaken.

As a consequence of the difficulties in COI sequence recovery, Hirai, Shimode, and Tsuda (2013) suggested adopting 28S as an alternative DNA barcode marker, but this switch would come at a cost. Because 28S evolves more slowly than COI, many closely allied species will lack sufficient substitutions to enable species‐level discrimination. By contrast, COI has demonstrated its capacity to distinguish known species in all lineages of freshwater zooplankton (Elías‐Gutiérrez, Martínez‐Jerónimo, et al., 2008; García‐Morales & Elías‐Gutiérrez, 2013; Jeffery et al., 2011; Montiel‐Martínez, Ciros‐Pérez, Ortega‐Mayagoitia, & Elías‐Gutiérrez, 2008), including closely related species complexes such as the cladoceran *Moina micrura* (Montoliu et al. 2015). In addition, DNA barcode studies have often revealed many species overlooked by prior taxonomic studies (Elías‐Gutiérrez & Valdez‐Moreno, 2008; Gutiérrez‐Aguirre, Cervantes‐Martínez, & Elías‐Gutiérrez, 2014; Miracle, Alekseev, Monchenko, Sentandreu, & Vicente, 2013; Montiel‐Martínez et al., 2008; Quiroz‐Vazquez & Elías‐Gutiérrez, 2009; Sukhikh & Alekseev, 2015), allowing more comprehensive research at broader taxonomic scales.

Considering the benefits and effectiveness of COI as the DNA barcode region for freshwater zooplankton, there is a clear need to develop protocols which enhance sequencing success. Prosser, Martínez‐Arce, and Elías‐Gutiérrez (2013) developed COI primers that substantially improved barcode recovery for cladocerans and copepods and showed that sequencing success was enhanced when samples were immediately fixed with chilled ethanol and then held for a week at −18°C. Furthermore, Prosser et al. (2013) demonstrated a drastic reduction in sequencing success when using 70% ethanol as a preservative, the concentration most commonly used by zooplankton researchers, in comparison with 95% ethanol. Applying these protocols to all taxa found in freshwater zooplankton samples may drastically improve COI sequence recovery for this group.

At the same time, there is a need for more efficient collection methods. Until now, zooplankton sampling in freshwater systems has relied almost entirely on vertical or horizontal net tows. By contrast, sampling programs in marine environments often also employ light traps. Although these traps were initially employed for collecting fish larvae (Vásquez‐Yeomans, Vega‐Cendejas, Montero, & Sosa‐Cordero, 2011), their value in collecting other groups has been recently recognized (Chan, Shao, Shao, & Chang, 2016). A few studies have used light traps in freshwater environments (Kehayias, 2006), but despite their potential ability to more comprehensively survey groups of freshwater zooplankton (Davies, 1976), their capacity to attract diverse taxa has seen little investigation.

Accordingly, the goals for this study are to demonstrate that (1) the use of light traps and traditional plankton nets for sampling give a more comprehensive survey of all species dwelling in a particular system; (2) the use of improved preservation techniques will increase the amplification success in a wide range of taxa present in a complex zooplankton sample; and (3) the use of a single set of primers will enable amplification of all groups present in the freshwater zooplankton.

## 2 METHODS

### 2.1 Collection and preservation of samples

**Figure 1:**
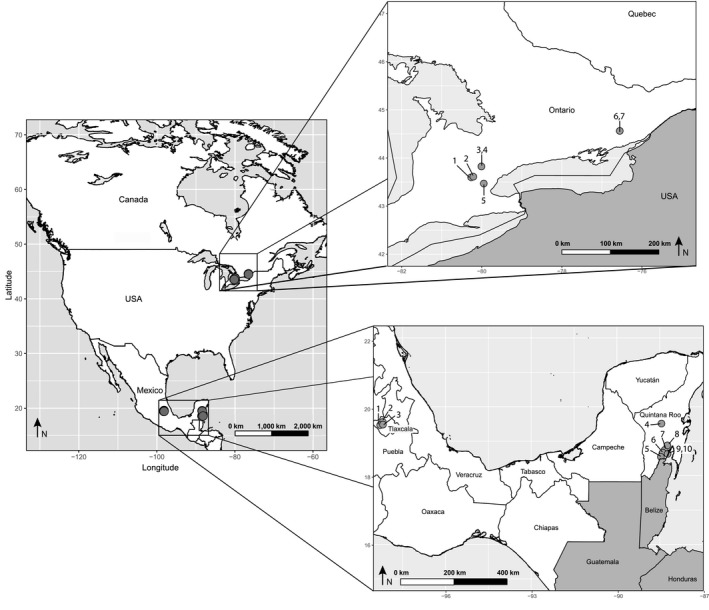
Map of sampling localities for this study

Samples were collected from 17 locations in nine water bodies (five in Mexico; four in Canada) (Table 1, Figure 1). Light traps (Jones, 2006) were deployed in the limnetic and littoral zones, plankton nets in the limnetic zone, and hand nets in the littoral zone. Plankton tows required just 10–15 min, while light traps were deployed overnight. All traps/nets employed a 50‐μm mesh, excepting those employed in two Mexican lakes (Bacalar, Xul Ha) where we also made collections with plankton nets with a 300‐μm mesh. Immediately after collection, samples were washed on a 50‐μm sieve with cold (4°C) 96% ethanol to extract any remaining water, before the specimens were transferred into a jar with approximately one‐third sample and two‐third ethanol. The sample jars were placed in a container with ice for transfer to the laboratory where they were then stored in a freezer at −18°C for at least 1 week. After this period, samples were stored at room temperature. The impact of this preservation protocol on sequence recovery was tested on specimens from lakes in Canada (temperate) and Mexico (tropical).

**Table 1:** Summary of collection locations, samples and taxa

Country	Site	Lat N	Long W	Specimens	Phyla	Barcodes	BINs	Collection method
Canada	1 Guelph Lake swamp	43.611	80.209	307	2	230	73	HN, TL
	2 Guelph Lake	43.599	80.264	188	3	159	28	HN
	3 Forks of the Credit Pond	43.824	80.008	30	1	22	13	HN
	4 Forks of the Credit Lake	43.823	80.007	38	1	26	16	HN
	5 Crawford lake	43.469	79.948	154	2	140	46	TL
	6 Eel Lake	43.563	76.554	95	2	77	33	TL
	7 Eel Lake (vegetation)	43.563	76.553	301	3	243	75	TL
Mexico	1 Cacaxtla	19.468	98.286	15	2	14	12	HN
	2 Atlangatepec	19.560	98.196	16	1	15	8	HN
	3 Chipila	19.481	98.191	44	3	39	24	HN
	4 Huay Pix	19.513	88.439	1	1	1	1	TL
	5 Bacalar (Buenavista)	18.880	88.231	7	1	7	3	TL
	6 Bacalar	18.746	88.325	8	1	8	2	HN
	7 Near Calderitas, pond in a road	18.631	88.225	23	1	23	7	
	8 Bacalar	18.667	88.394	9	1	9	2	TL
	9 Bacalar (XulHa)	18.544	88.460	19	2	16	8	RPL
	10 Bacalar (Cocalitos sinkhole)^a^	18.651^a^	88.409^a^	881	4	809	84	TL, RPL5 and 3, HN
Total				2,136	4	1,838	325	

Valid barcodes are considered those that allowed the specimen identification.

TL, light trap; RPL, horizontal plankton net tow (10 min in the night), five with 50 μm, three with 300 μm; HN, hand net 50 μm.

a Main collection locality in Bacalar Lake.

In several systems (Canada: Guelph Lake, Eel Lake, Crawford Lake; Mexico: Bacalar Lake), replicate samples were simultaneously collected to test the effect of denatured (methylated) versus nondenatured ethanol treatments, where two samples were fixed in 4°C ethanol (denatured and nondenatured ethanol) followed by chilling on ice, while the other two were fixed and stored in room temperature ethanol (denatured and nondenatured) at 20–25°C. Other samples from Central Mexico (Atlangatepec, Cacaxtla, and Chipila) were fixed following only the standard procedures suggested by Prosser et al. (2013).

All specimens collected by the light traps, plankton nets, and hand nets are treated as zooplankton, although some collected by hand nets were clearly displaced from the substrate. We compared the number of taxa recovered with net sampling and light traps in Bacalar and Guelph Lake.

### 2.2 Specimen preparation

All specimens were sorted under a stereomicroscope, and representatives of each morphologically distinct taxon were photographed using a compound or stereo microscope. Following photography, specimens were again placed in ethanol and stored at room temperature. *z*‐axis stacked images were generated for some specimens using Helicon Focus 6.5.1 software.

### 2.3 DNA extraction and amplification

DNA was extracted using a standard glass fiber method (Ivanova, DeWaard, & Hebert, 2006) from whole individuals in the case of small taxa (e.g., cladocerans < 0.3 mm). In case of water mites, voucher specimens were recovered following Porco, Rougerie, Deharveng, and Hebert (2010) and preserved in 96% ethanol with a drop of glycerol. All other small specimens were destructively analyzed. DNA was extracted from tissue samples in the case of larger specimens. For cladocerans, the embryos or second antenna was used. In case of copepods, the dorsal muscles of the cephalothorax or the eggs (in carrying egg females) were extracted. In other groups, as insects, only one fraction of a leg was dissected. Finally, for fish larvae, one eye of the right side was used. In all cases, the vouchers (specimens not lost during extraction) are deposited in the Reference Collection at the El Colegio de la Frontera Sur, Unidad Chetumal (Access Numbers ECO‐CH‐Z‐09536‐ECO‐CH‐Z‐09696).

Following DNA extraction, 2 μl of each DNA extract was added to a PCR mixture consisting of 2 μl of Hyclone ultrapure water (Thermo Fisher scientific), 6.25 μl of 10% d‐(+)‐trehalose dihydrate (Fluka Analytical), 1.25 μl of 10X Platinum Taq buffer (Invitrogen), 0.625 μl of 50 μmol/L MgCl_2_ (Invitrogen), 0.0625 μl of 10 μmol/L dNTP (KAPA Biosystems), 0.125 μl of each 10 μmol/L primer (Zplank primers, see Prosser et al., 2013 for details), and 0.06 μl of PlatinumTaq (Invitrogen). All specimens (1,978 of 2,136) were amplified with the Zplank primers, except 158 fish larvae, where the C‐Fish cocktail (Ivanova, Zemlak, Hanner, & Hebert, 2007) was used. These fish are found in the Bacalar Fish I project. We compared performance of Zplank primers versus C‐Fish cocktail in 106 specimens. The reactions were cycled at 95°C for 1 min, followed by five cycles of (94°C for 40 s, 45°C for 40 s, 72°C for 1 min), then 35 cycles of (94°C for 40 s, 51°C for 40 s, 72°C for 1 min), and a final extension of 72°C for 5 min. PCR products were visualized on a 2% agarose gel using an E‐Gel 95 well Precast Agarose Electrophoresis System (Invitrogen), and those showing a PCR product were selected for sequencing.

### 2.4 Sequencing and data analysis

PCR products were cycle sequenced using a modified (Hajibabaei et al., 2005) BigDye^©^ Terminator v.3.1 Cycle Sequencing Kit (Applied Biosystems, Inc.), and sequenced bidirectionally on an ABI 3730XL automated sequencer using M13F and M13R primers. Sequences were edited using CodonCode v. 3.0.1 (CodonCode Corporation, Dedham, MA) and uploaded to BOLD and are available in the dataset freshwater zooplankton from light traps (DS‐LTZPL; http://www.boldsystems.org/index.php/Public_SearchTerms?query=DS-LTZPL). All data were analyzed with the tools on BOLD, and all sequences were examined for the presence of stop codons and indels as a check against NUMTS (Ratnasingham & Hebert, 2007). In addition, all sequences are available on GenBank via accessions MG448608‐MG450325.

A Neighbor‐Joining tree was calculated with the Kimura two‐parameter (K2P) distance model (Kimura, 1980) for each major group (Rotifera, Mollusca, Arachnida, Crustacea, Insecta, and Actinopterygii) using the analytical tools provided by BOLD. We selected this method because it allows the rapid analysis of large datasets and for species delimitation (Mutanen et al., 2016). Simplified trees were prepared using Mega 7.0 (Kumar, Stecher, & Tamura, 2016).

All sequences which met minimum quality standards (>500 bp, <1% ambiguous bases, free of stop codons, and contamination) were assigned a Barcode Index Number (BIN) on BOLD (Ratnasingham & Hebert, 2013). Because taxonomic systems are poorly developed for most freshwater invertebrate groups and overlooked species are common (Balian et al., 2008), we generally employ the BIN system as a proxy for species (Ratnasingham & Hebert, 2013), even though we refer to taxa by their species name in some cases.

To establish the efficiency of the collecting method, accumulation curves and Shannon Index diversity values were calculated for all BINs using the tools on BOLD 4.0 for two systems: one Mexican lake (Bacalar) and one from Ontario (Eel Lake). Accumulation curves were also extrapolated to a maximum of 2,000 individuals in EstimateS (Colwell, 2013), and total BIN richness for each system was estimated using the classic Chao1 estimator in EstimateS.

Finally, we compared sequencing success rates by region (Tropical and Temperate), preservation method (Ice vs. No Ice), and type of ethanol used (Denatured vs. Nondenatured). For Actinopterygii, we also compared the use of the Zplank primers (Prosser et al., 2013) versus the Fish Cocktail primers (Ivanova et al., 2007). Total success rate for each sample was obtained per sample and factor (Support file 2). We then performed a nonparametric multivariate analysis of variances (NPMANOVA; Anderson, 2001 as in the R‐package “vegan” v. 3.4.1, using full orthogonal model with Euclidean distance metric. We also applied a *betadisper* (Oksanen et al., 2017) analyses to establish the differences in group homogeneities that is analogous to Levene's test of the equality of variances (Anderson, 2006; O'Neill & Mathews, 2000). In this case, each treatment was a group (region, preservation method, and type of ethanol). Boxplots with the distance to the centroid for the Arachnida, Crustacea, Insecta, and Actinopterygii with each treatment were also calculated.

## 3 RESULTS

**Figure 2:**
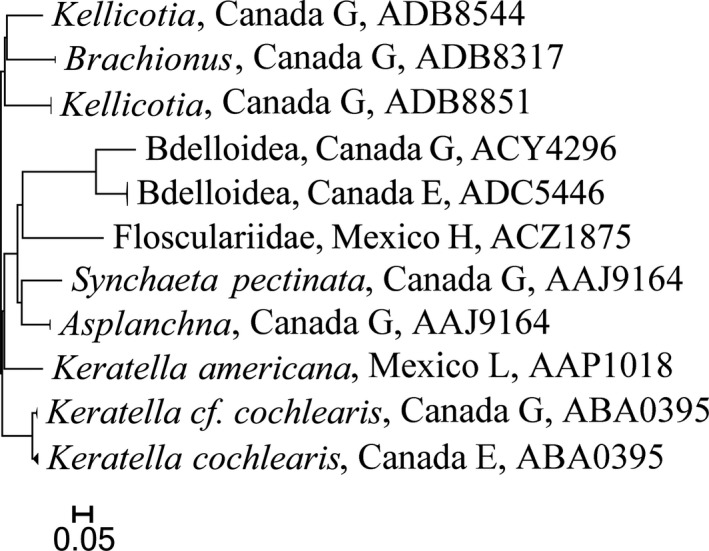
Simplified tree for Rotifera. After the taxonomic name is the country of collection and location (G, Guelph Lake; E, Eel Lake; H, highlands, 2,000 m or more above sea level; L, lowlands, near sea level in Yucatan Peninsula). Last number is the BOLD assigned BIN

**Figure 3:**
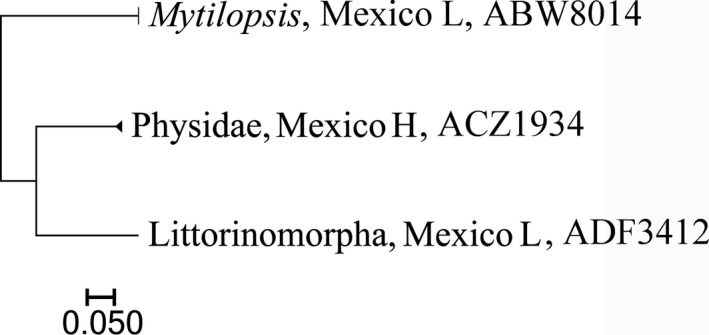
Simplified tree for Mollusca. After the country name is the location (H, highlands, 2,000 m or more above sea level; L, lowlands, near sea level in Yucatan Peninsula). Last number is the BOLD assigned BIN

**Figure 4:**
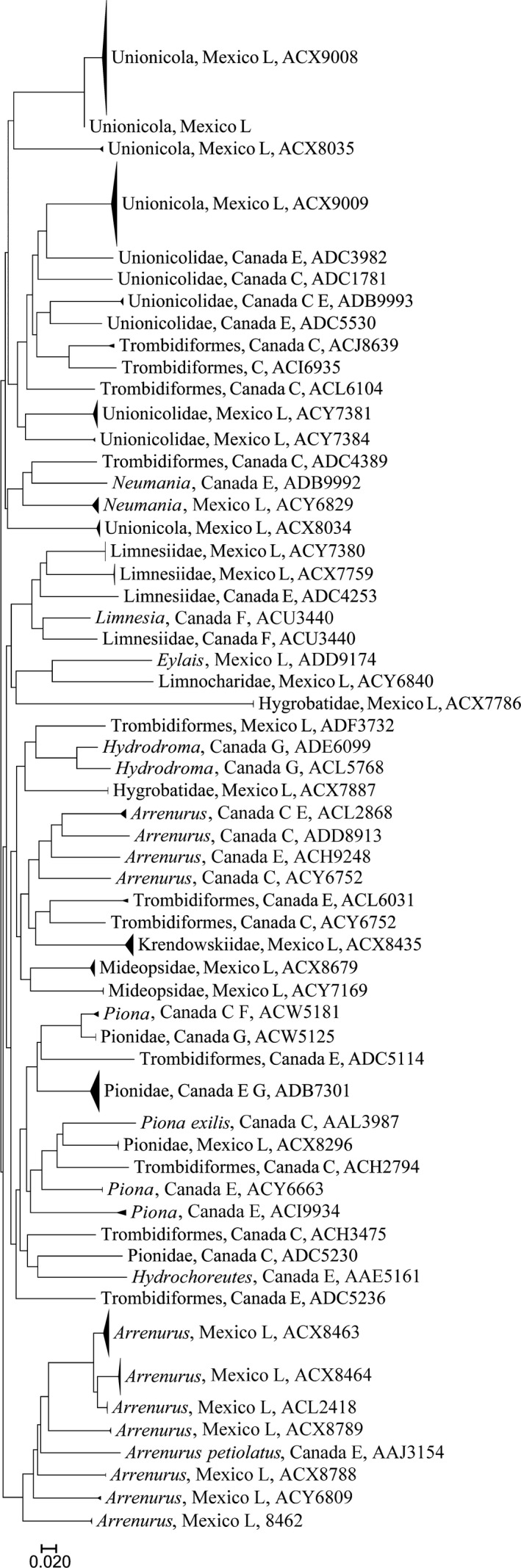
ID simplified tree for Arachnida. After the taxonomic name is the country of collection and location (G, Guelph Lake; E, Eel Lake; F, Forks of the Credit Lake, H, highlands, 2,000 m or more above sea level; L, lowlands, near sea level in Yucatan Peninsula). Last number is the BOLD assigned BIN

**Figure 5:**
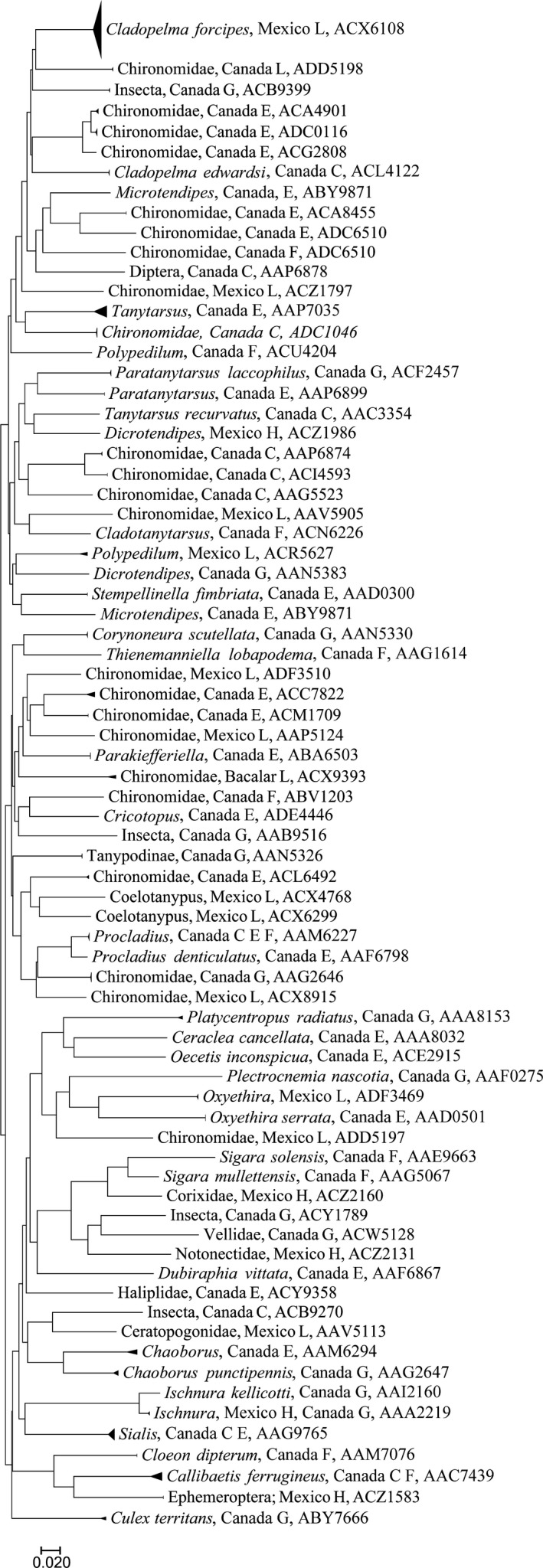
Simplified tree for Insecta. After the taxonomic name is found the country of collection and location (G, Guelph Lake; E, Eel Lake; F, Forks of the Credit Lake, H, highlands, 2,000 m or more above sea level; L, lowlands, near sea level in Yucatan Peninsula). Last number is the BOLD assigned BIN

**Figure 6:**
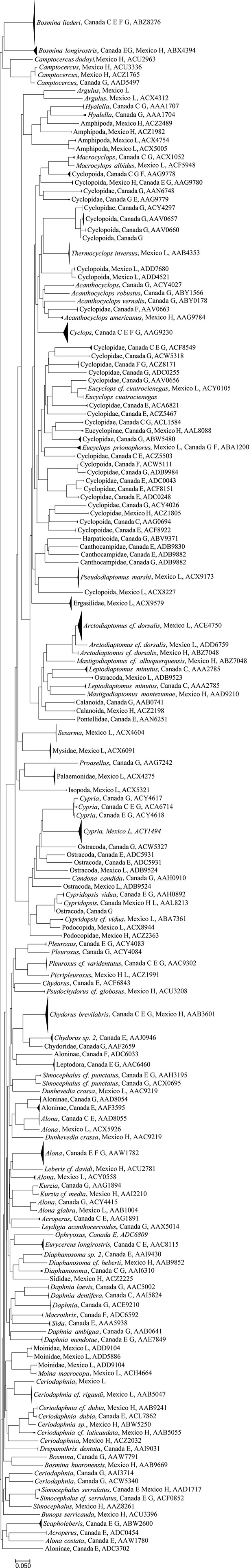
Simplified tree for Crustacea. After the taxonomic name is found the country of collection and location (G, Guelph Lake; E, Eel Lake; F, Forks of the Credit Lake, H, highlands, 2,000 m or more above sea level; L, lowlands, near sea level in Yucatan Peninsula). Last number is the BOLD assigned BIN

**Figure 7:**
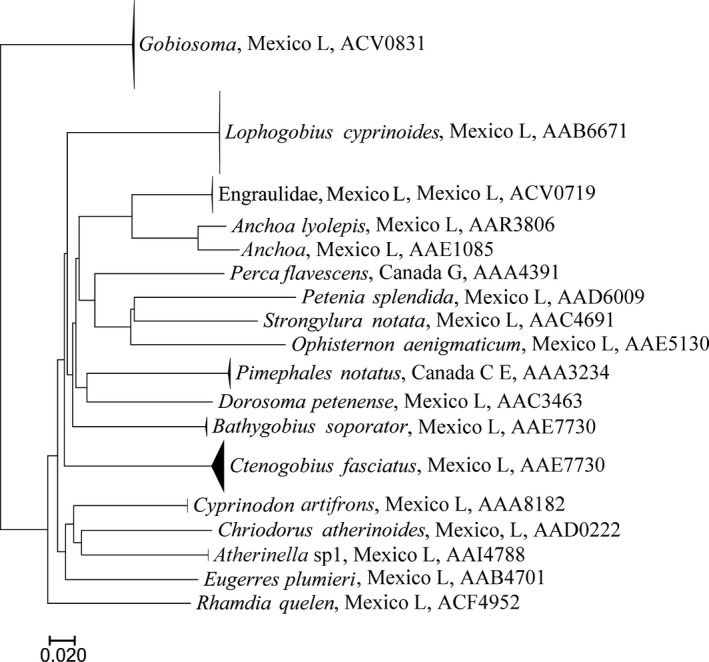
Simplified tree for Actinopterygii. After the taxonomic name is found the country of collection and location (G, Guelph Lake; C, Crawford Lake; E, Eel Lake; H, highlands, 2,000 m or more above sea level; L, lowlands, near sea level in Yucatan Peninsula). Last number is the BOLD assigned BIN

Overall, sequences were recovered from 1,864 of the 2,136 specimens that were analyzed (Support file 3) and most (1,838) were long enough to allow assignment to an order or lower taxonomic level (Table 1, Support file 3). In total, 1,767 records received a BIN assignment revealing 325 BINs comprising rotifers, mollusks, mites, crustaceans, insects, and fishes (Figures 2, 3, 4, 5, 6, 7).

**Figure 8:**
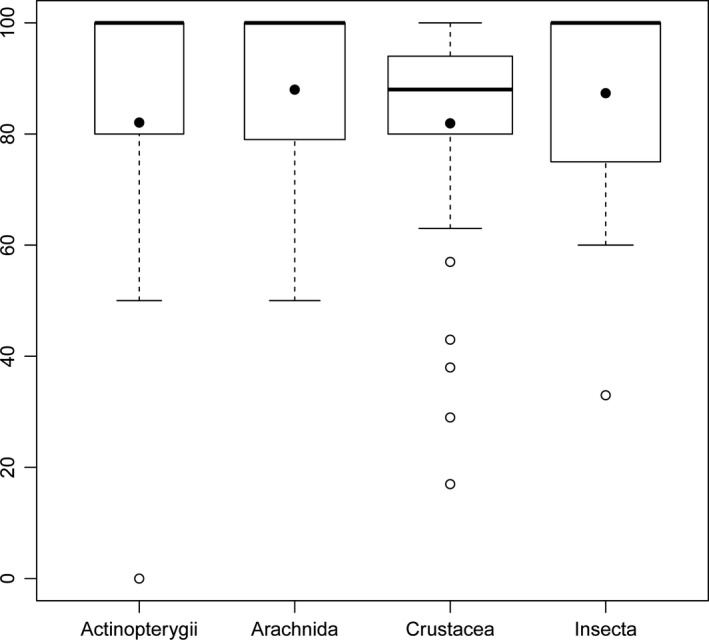
Boxplots for overall sequencing success and dispersion from the median in each major group. Rotifera and Mollusca are not included, because in them, success was 100%. Variable width of the boxplot is related to the number of specimens processed. Dispersion is from the median. Filled circles are the mean. Open circles are outliers

Among the 1,864 sequences recovered, just 24 were contaminants (Table 2). As a result, valid sequences were obtained from 1,821 specimens, a mean success rate of 85.3%. Sequencing success across the seven classes ranged from 100% for Rotifera and Mollusca, to 82.01% for Actinopterygii, 88.0% for Arachnida (water mites), 81.93% for Crustacea, and 87.36% for Insecta (Figure 8).

**Table 2:** Taxonomic and sequence summary among groups

Taxa	Orders	Families	Specimens processed	Contaminated sequences	Barcode compliant sequences (%)	BINs
Rotifera	2	3	27	0	92.6	11
Mollusca	2	2	9	0	100.0	3
Arachnida	1	11	407	14	82.1	59
Crustacea	4	22	1,282	9	80.0	161
Insecta	7	14	213	1	80.4	75
Actinopterygii^a^	11	15	196	0	69.7	16
Not identified	0	0	2	0	0	0
Total	27	67	2,136	24	79.02	325

The number of orders and families may change with further curation of the specimens.

a Two sets of primers were used for this group (see text), and all others were analyzed only with Zplank primers.

Among the major groups, crustaceans were most diverse with 161 BINs (Figure 6, Table 2) from 1,080 specimens. By comparison, just 11 BINs of rotifers were detected, nine from Canada and two from Mexico (Figure 2, Table 2). Only three BINs of Mollusca from Mexico were found (Figure 3, Table 2).

From the crustaceans, the cladocerans and copepods are traditionally considered the most representative of freshwater zooplankton. In total, the 510 sequences from cladocerans were assigned to 72 BINs, 29 from Mexico, and 45 from Canada. From these, only two species were found in both Canada and Mexico, *Chydorus brevilabris* represented by BIN AAB3601 and *Simocephalus serrulatus*, BIN AAD1717. Neither of these shared species occurred in the Mexican lowlands. Only one specimen of *Picripleuroxus* from the highlands was found also in the Mexican lowlands. Four genera found in both Canada and Mexico were assigned to different BINs and different branches of the ID tree (*Diaphanosoma*,* Ceriodaphnia*,* Bosmina*, and *Kurzia*) (Figure 6, Support file 1).

Among the copepods, diversity was low for Calanoida with nine BINs from México, six from Bacalar Lake (three of them were singletons), and three from the highlands. Five calanoid BINs were detected in Canadian lakes. Cyclopoids were substantially more diverse than calanoids, with 33 BINs from Canada, including 12 from Eel Lake. In the tropics, 12 BINs were found, six from Bacalar and four from the highlands. Two more BINs were found in small pools near the shore of Chetumal Bay (Calderitas town). The only species detected in both the Mexican tropics and Canada with more than one specimen was *Eucyclops prionophorus* (BIN: ABA1200) (Figure 6, Support files 1 and 3).

Ostracods were represented by five BINs from Bacalar and eight from Canada. However, many of the BINs in Bacalar were common, while half of the Canadian ostracod BINs were singletons (Figure 6, Support files 1 and 3).

The Insecta were the second most diverse group, with 75 BINs from 213 specimens. However, almost half of the specimens could not be identified to a genus level (49.0%) either by morphology or comparison of their sequences to records on GenBank and BOLD. It is likely that many more taxa await collection as 10 genera from Canada were represented by less than five specimens. From the total, 63% were dipterans represented by four families and 16 genera, and the remaining belong to five orders represented by insect larvae (Ephemeroptera, Hemiptera, Megaloptera, Odonata, and Trichoptera) and one more order by adults (Coleoptera) (Figure 5, Support files 1 and 3).

The third most diverse group was the Arachnida, represented by 59 BINs, all of them are water mites (Figure 4, Support files 1 and 3) from the order Trombidiformes. Of those, 26 are from Mexico and the remaining 33 are from Canada, with no overlap of species between the tropics and temperate samples. Only two mite species, *Arrenurus petiolatus and Piona exilis*, could be identified. Of the remaining BINs, 27 were identified to genus, 18 to family, and 12 just to order. Most of the BINs detected belonged to *Arrenurus* (Arrenuridae), *Neumania* (Unionicolidae), or *Piona* (Pionidae). From the total, 65 specimens lack species identification (BOLD, accessed August, 2017), but this number will change continuously after curation of specimens.

Actinopterygii was represented by 16 BINs from 181 specimens (Figure 7, Support files 1 and 3). From these, 12 could be identified to species. Two more, with short sequences but identifiable, could also be identified totaling 14 recognized species. From these, only two, *Perca flavescens* and *Pimephales notatus*, were collected in Canada, all others from Mexican lowlands. Of the remaining BINs with no species found, three were identified to genera (*Gobiosoma* and *Atherinella)*, and one to family (Engraulidae).

**Figure 9:**
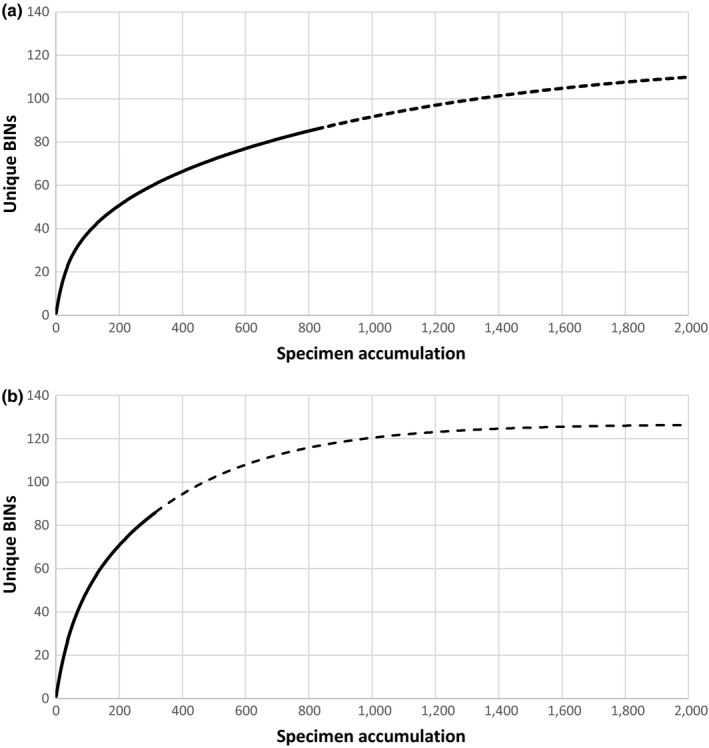
Accumulation curve for BINs in two lakes. (a) Bacalar Lake (18 sampling dates) (b) Eel Lake (27–29 May 2016). Dotted line is the extrapolation

The Shannon index was high (>3.0) in all cases and showed little variation. For example, a single sampling event in Bacalar Lake (April 18, 2015) resulted in a Shannon index of 3.36, which reached a high of 3.69 considering all 18 sampling events in this system. Crawford Lake, similar to the karstic oligotrophic sinkhole in Bacalar Lake, had a slightly lower value (3.28). However, the Shannon index of Eel Lake reached 4.02 from just one sampling event. In addition, accumulation curves for Bacalar Lake and Eel Lake (Figure 9a,b) reach a similar level of diversity even though Bacalar Lake (Figure 9a) includes a larger sample size (754 barcoded specimens and 18 sampling dates) than Eel Lake (315 specimens and two nights sampling) (Figure 9b). However, when the BIN accumulation curves are extrapolated to a sampling effort of 2,000 individuals, the Eel Lake curve reaches a plateau at around 120 BINs while the Bacalar Lake curve continues to rise despite reaching a lower level of BIN richness. In addition, the Chao1 measures indicate total BIN richness of 126 for Eel Lake (95% confidence intervals = 103.44–180.98) and 120 for Bacalar Lake (95% confidence intervals = 98.87–173.81).

### 3.1 Comparison of trapping methods

**Figure 10:**
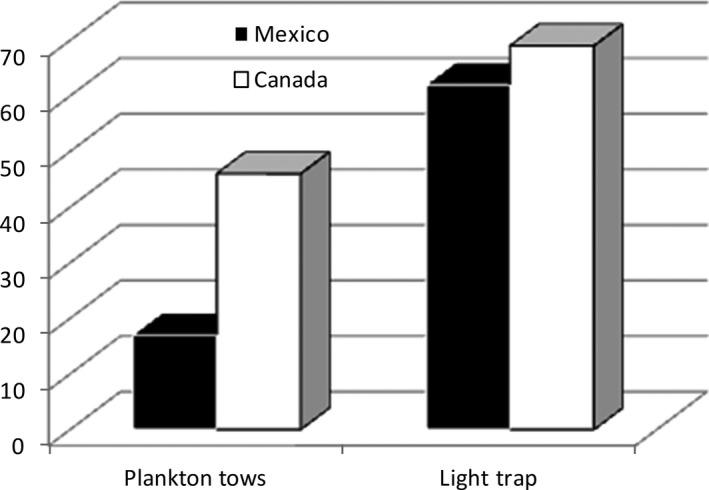
Number of taxa captured with light traps and horizontal tows in Bacalar and Guelph Lake

We compared light traps and plankton tows in tropical (Bacalar Lake) and temperate regions (Guelph Lake and Guelph Lake swamp). BIN richness of plankton tows was lower than light traps (Figure 10), although BIN richness was higher overall in Guelph Lake sites.

Several taxa were more predominantly represented in the light trap samples compared to the plankton tows. For example, all BINs from Canada collected in the tows were also attracted by the light trap excepting the singleton BIN ACY4296, a Bdelloid rotifer. However, in the samples from Mexico, *Keratella americana* (BIN AAP1018) was the only rotifer collected by the light trap. In addition, the Arachnida were more diverse in light traps, represented by at least 11 families (Table 2) and 10 genera of water mites (Trombidiformes: Prostigmata: Hydrachnidiae) (Figure 4, Support file 1).

Insects were another important group which was collected solely by light traps. Curiously, some Chironomidae larvae apparently emerged as adults inside of the light traps. Furthermore, Coleoptera were represented by just four specimens and two BINs which were found in the light traps (Figure 5). Representatives of five other insect orders were also collected in the light traps (Ephemeroptera, Hemiptera, Megaloptera, Odonata, and Trichoptera).

The only group of chordates attracted by the light trap was the Actinopterygii, most of which were larvae (Figure 7). Four BINs were collected by tows and 11 BINs collected by light trap in Bacalar Lake. The ichthyoplankton fauna from Bacalar was surprisingly rich, and 10 of the 14 BINs could be identified to a species. In Guelph Lake, the light trap trapped just one species, *P. flavescens* represented by one BIN. In Crawford and Eel lakes, only *P. notatus* was attracted to the light traps.

A total of 86 BINs were detected in Eel Lake after deploying a single light trap for two nights, including two Rotifera, 26 Cladocera, 16 Copepoda, four Ostracoda, 15 Hydracarina, and 16 Chironomidae. The first sample, collected in the limnetic zone, revealed 33 BINs, and the second sample collected among the littoral vegetation resulted in 75 BINs (Table 1). Eleven BINs from the limnetic sample were absent from the vegetation sample. The most common cladoceran taxa that avoided the littoral zone were *Leptodora*,* Chaoborus* and *Bosmina longirostris*. A species considered benthic, *Drepanothrix dentata*, was also found in the limnetic sample, but not in the littoral.

### 3.2 Comparison of fixation methods and primers used

**Figure 11:**
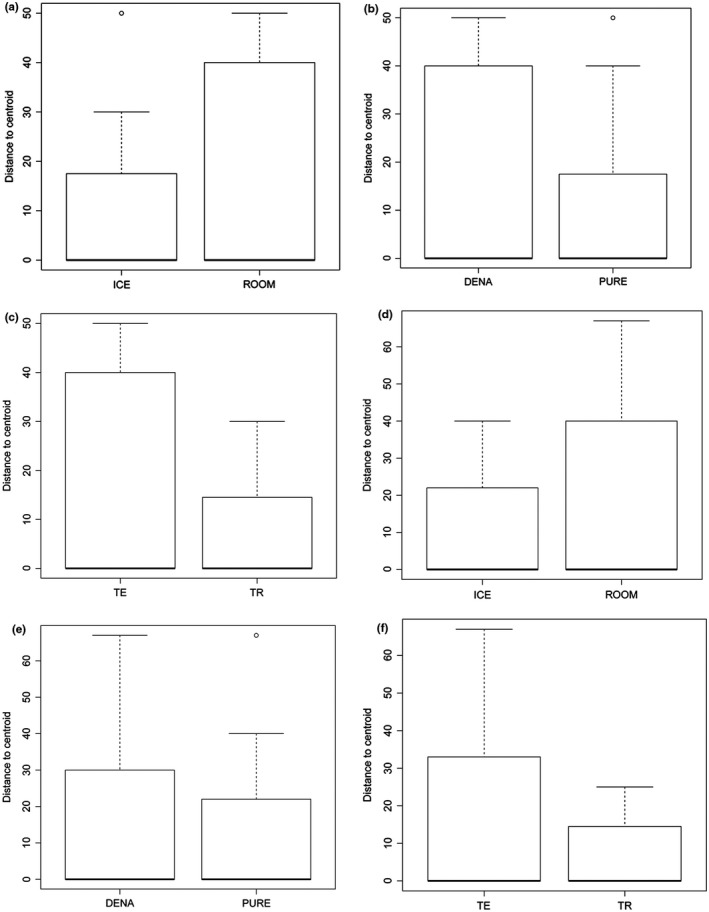
Boxplot of distance to centroid for different treatments and sequencing success. (a–c) Arachnida (d–f) Insecta. ICE, Preservation with ice; ROOM, preservation to room temperature; DENA, denatured ethanol; PURE, nondenatured ethanol; TE, temperate region; TR, tropical region

**Figure 12:**
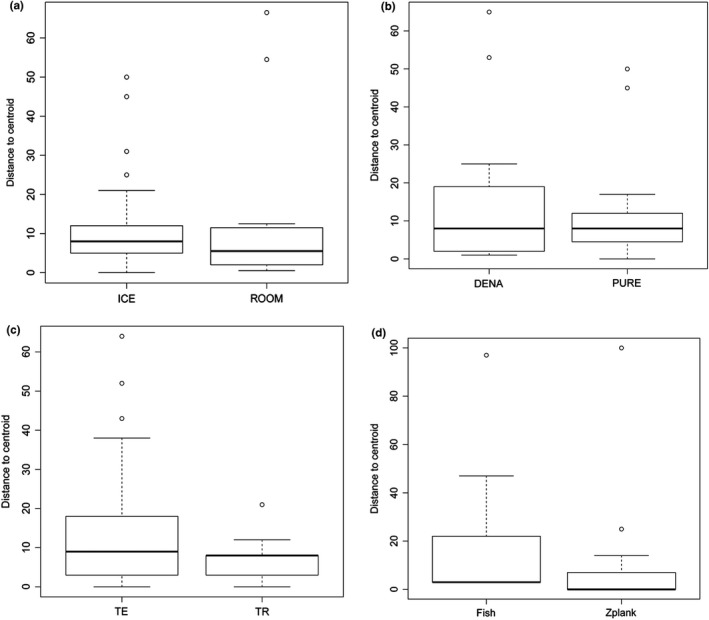
Boxplot of distance to centroid for different treatments and sequencing success. (a–c) Crustacea (d) Actinopterygii. ICE, Preservation with ice; ROOM, preservation to room temperature; DENA, denatured ethanol; PURE, nondenatured ethanol; TE, temperate region; TR, tropical region; Fish, C‐Fish primers (Ivanova et al., 2007); Zplank, Zooplankton primers (Prosser et al., 2013)

Significant differences in sequencing success rate were detected within the interaction of Region/Preservation in Arachnida (Table 3, Figures 11 and 12), showing a positive interaction, indicating that the use of ice has differing effects on sequencing success in both regions. In crustaceans, all interactions were significant (Table 3), but the betadisper analyses were also significant for region (*p* = .03), indicating either poor sampling representation in both regions or differences in the handling of samples between regions.

**Table 3:** Results of the NPMANOVA and *betadisper* analyses. In all cases, the number of permutations was 999

Taxon	Arachnida		Insecta		Crustacea		Actinopterygii	
Interaction	*F* value	*p* Value	*F* value	*p* Value	*F* value	*p* Value	*F* value	*p* Value
Preservation/Alcohol	1.092	.342	1.5403	.181	3.315	.035*	0.342	.76
Region/Alcohol	0.795	.476	1.253	.321	6.210	.007*	0.523	.718
Region/Preservation	3.193	.041*	2.210	.094	4.164	.017*	0.343	.796
Primer/Preservation		NA		NA		NA	0.345	.759
Primer/Alcohol		NA		NA		NA	0.613	.636
Primer/Region		NA		NA		NA	0.256	.839
*Betadisper*								
Alcohol	0.970	.333	0.541	.467	1.914	.173	0.994	.329
Region	1.529	.226	2.530	.120	5.075	.029*	0.121	.731
Preservation	2.198	.149	2.556	.119	0.547	.463	0.579	.454
Primer		NA		NA		NA	0.522	.477

Significant *p* values are marked with “*”.

In case of Actinopterygii, the only group where two types of primers were compared, there was no significant difference between the use of C‐Fish cocktail (Ivanova et al., 2007) and Zplank (Prosser et al., 2013) primers (Table 3, Figure 12d). The Zplank primers generated an average success of 89.5% for fishes, just slightly less than the C_Fish primers (91.8%) (Figure S1).

## 4 DISCUSSION

### 4.1 Overall sequencing success

This study represents the most extensive assessment of freshwater zooplankton using DNA barcodes to date. In total, representatives from 27 orders belonging to four phyla were analyzed using DNA barcodes (Table 2). In all cases, sequence recovery was high (≥82%), with just 24 contaminations detected. Fourteen of these involved mites from Bacalar Lake, which could represent unintentional gut content amplification (see Support file 1) revealing the prey of these mites. However, the perfect rate of sequence amplification seen in rotifers and molluscs (both 100%) should be considered with care as sample sizes were limited in these groups (Table 2).

Because of their high success rate observed over a broad range of taxa (Rotifera, Mollusca, Arthropoda, and Chordata), the Zplank primers may be useful for COI amplification in many other taxa. For example, no significant differences in sequencing success were detected in comparison with the C‐Fish primer cocktail (Ivanova et al., 2007) when used for COI amplification of fish (Figure 12d, Table 3, Figure S1). In addition, we were able to successfully amplify COI DNA barcodes from all taxa sampled, even groups which are difficult to amplify using traditional primer sets (like Crustacea). One possible reason for their high success rate is that they are partially degenerate as noted in Prosser et al. (2013), with one degenerate base “R” (“A” or “G”) in the forward ZplankF1_t1 primer and similarly one degenerate base “Y” (“C” or “T”) in the reverse Zplank R1_T1 primer. However, further research is need to assess the true extent of the true universal application of the Zplank primer set.

### 4.2 Species richness and distribution

Species richness ranged from a high of 161 BINs in the Crustacea (Figure 6) to a low of three BINs in Mollusca (Figure 3). The aquatic mites (Arachnida, BINs = 59) (Figure 4) and insects (BINs = 75; Figure 5) were also well represented, and although only 16 fish BINs were detected, they were present in relatively high abundance (196 specimens). BIN overlap between Mexican and Canadian samples was minimal. Only two cladoceran BINs and two copepod BINs were shared between Mexico and Canada. However, one of the two shared copepod BINs were represented by a single sequence, and further sampling is required to confirm their broad distribution.

Species richness was high in certain temperate locations. Specifically, the two light trap samples taken from Eel Lake revealed 86 BINs. Similar sampling from Crawford Lake near Guelph, Ontario, resulted in 46 BINs, but this system is impoverished by ancestral human impacts (Ekdahl et al., 2007). Besides, many of the BINs from Crawford Lake were singletons, suggesting either low sample coverage or perhaps a high proportion of rare species due to its nature.

This study represents a first step in exploring the diversity of water mites in the tropics. They are poorly known despite their value as bioindicators in freshwater ecosystems, even at the generic level (Goldschmidt, 2016). For example, members of the genus *Limnesia* (Limnesiidae) are very sensitive to poor water quality (Goldschmidt Helson, & Williams, 2016). As such, the abundance of Limnesiidae in Lake Bacalar suggests a relatively pristine freshwater habitat.

Of the molluscs, all three BINs were found only in Mexico. One of these BINs (ABW8014), identified as *Mytilopsis*, is interesting as only a single species is known from Lake Bacalar (*M. sallei*, recorded by Marelli & Berrend, 1978). However, BIN ABW8014 shows >5.8% divergence from specimens of this species from Darwin, Australia, where the species was introduced through ballast water discharge. The sequence divergence may indicate the misidentification of specimens from Bacalar or Darwin, but the analysis of *M. sallei* from its type locality (Lake Izabal, Guatemala) (Marelli & Berrend, 1978) should clarify the situation.

Among the crustacea, the cladocera were rare in Bacalar Lake and in general all samples from Yucatan, despite intensive sampling efforts, confirming the earlier conclusion (Smirnov & Elías‐Gutiérrez, 2011) of low cladoceran diversity and abundance in the Yucatan Peninsula. As expected, calanoid fauna (Crusacea: Copepoda: Calanoidea) was also depauperate in each system, as the detection of more than three calanoid species in any freshwater system is rare (Elías‐Gutiérrez, Suárez‐Morales, et al., 2008). The three singleton calanoid BINs from Bacalar should be analyzed with care, because they form part of the *Arctodiaptomus dorsalis* complex that still is unresolved and was detected in earlier studies from the tropical southeast of Mexico and Guatemala (Elías‐Gutiérrez, Martínez‐Jerónimo, et al., 2008). As in cladocera, members of particular genus such as *Macrocyclops* (Crusacea: Copepoda: Cyclopoida) found in both, Bacalar Lake and two Canadian localities, clearly fell into two distinct clusters (Figure 6), confirming previous conclusions about the prevalence of cryptic species in this genus (Karanovic & Krajicek, 2012).

Although the insect fauna of Canada has been heavily surveyed using DNA barcodes (i.e., Steinke, Breton, Berzitis, & Hebert, 2017), the DNA barcode‐based identification of chironomids, especially larvae, remains extremely difficult and in progress in the Great Lakes (e.g., Failla, Vasquez, Hudson, Fujimoto, & Ram, 2016). Because of this, only eight of the 35 BINs detected from 79 specimens could be identified to the species level. In general, most of the species found for all groups still lack lower level taxonomic identifications. From all other insect orders represented by 26 BINs, 13 were identified to species level, all of them from Canada. Lack of knowledge is worst in the tropics. None of the five BINs found in Mexico could be identified to species, and just one was identified to genus, all others just to the family level.

Of the 16 fish BINs detected, two abundant BINs could only be identified to a genus (*Atherinella*,* Gobiosoma*) and one more to a family (Engraulidae). These species seem to be common and could be new records or new taxa requiring careful revision. The collection of larval *Cyprinodon artifrons* in the Cocalitos sinkhole within Bacalar Lake and some juveniles in a nearby wetland was surprising because adults have been found in the reef lagoon in Xcalak, Chetumal Bay (Valdez‐Moreno, Vázquez‐Yeomans, Elías‐Gutiérrez, Ivanova, & Hebert, 2010), which is a marine environment. Although this species is considered brackish, their larval stages were unknown previously. This is the first documented evidence for the use of Lake Bacalar as a refugee for the juvenile stages of a fish species whose adults have been collected in marine waters. We consider that its high carbonate salinity allows it to serve as a refugee for some marine or brackish species. Nevertheless, there is no direct connection between Bacalar Lake and the sea (Perry, Velazquez‐Oliman, & Marin, 2002), and since migratory routes are unknown it is possible that these larvae disperse underground or through the complex system of wetlands associated with the nearby Hondo River.

We consider that the levels of diversity we detected in tropical lowlands are not typical, as Bacalar Lake, the most heavily sampled system in our study, is an extreme environment (Perry et al., 2002), oligotrophic, with strong development of giant stromatolites (Gischler, Gibson, & Oschmann, 2008). Its waters are supersaturated with calcite due to the drainage system passing thru an area of gypsum‐bearing rocks (Perry et al., 2002), making this environment quite unique and extreme. This effect is clearly seen in the resident fauna, because most of the freshwater groups found here are saline tolerant, such as the *Ceriodaphnia*, a cladoceran (Lazareva, Gusakov, Zinchenko, & Golovatyuk, 2013), the mysids, amphipods, palaemonids, sesarmids, and isopods. In addition, species richness values of 84 putative species (BINs) and a potential of 120 in this lake, with 31 of them being crustaceans, are high when compared with any other neotropical lake. For example, Aranguren‐Riaño, Guisande, and Ospina (2011) found the same number of crustacean species in 15 lakes from Colombia, with a total of 33 sampling points distributed across the Amazon, Norandean, and Peri‐Caribbean regions using plankton tow nets. Yet one collecting event (using light traps) detected nearly half of the total BINs accumulated after 18 different sampling events from various locations in Bacalar Lake.

Accumulation curves for light traps in Bacalar in comparison with Eel Lake (Figure 9a,b) reveal that twice as many BINs were obtained in the temperate region after only two nights of sampling. The low abundance of many species in conjunction with high diversity indices (Shannon) detected in tropical systems indicates that additional sampling effort in Bacalar is required for thorough assessment of the zooplankton community. On top of this, many species were rare (47.6% doubletons and singletons), particularly in the chironomids, mites, chydorids, and copepods. Similarly, many singleton and doubleton BINs were detected in Eel Lake (36%), but sampling effort was not comparable with Bacalar (321 vs. 800 sequences). However, the expected species richness estimate of 120 BINs for Bacalar Lake remains comparable with the estimate of 126 BINs for Eel Lake, further supporting the observation of high diversity and low abundance of many taxa in the tropical light trap samples. It is evident the need for more ample effort in sampling this tropical lake.

### 4.3 Comparison of trapping methods

Light traps have traditionally been used to collect marine zooplankton (Chan et al., 2016), although they have occasionally been used to survey metazoan communities in freshwater ecosystems (Kehayias, 2006; Nikolaeva, 2008). Most studies target‐specific fauna such as beetles (Klecka & Boukal, 2011), mosquitoes (Graber, 1996), trichopterans (Collier, Smith, & Baillie, 1997), and chironomids (Goretti, Coletti, Di Veroli, Di Giulio, & Gaino, 2011). However, Davies (1976) report of a wide range of taxa attracted to light traps (at least 12 major groups besides chironomids) has gone largely unnoticed. As such, no detailed reports on the taxa caught by light traps in freshwater lentic systems have been published.

Consequently, our study is the first to broadly explore metazoan biodiversity of freshwater systems using light traps. We found that all taxa sampled through plankton netting were also detected with light traps, except the mussel larvae *Mytilopsis*. In addition to this, light traps comprised higher species richness and taxonomic diversity than the plankton net samples. The majority of the species collected by light traps represent new records of unknown species, especially in the Bacalar Lake region with BINs new to BOLD including many crustaceans with marine affinities, such as the mysids. A similar combination of methods has been previously suggested for a nearby marine locality (Bacalar Chico), but this study only considered fish larvae (Vásquez‐Yeomans et al., 2011). We can say that light traps collect more species richness and broader taxonomic diversity (four phyla, 11 classes, 34 orders) than plankton net samples, with nearly complete species coverage of the plankton net samples from similar regions. Their utility is evident as an efficient sampling method for most freshwater metazoans when they are deployed in both the littoral and limnetic regions.

### 4.4 Comparison of fixation methods

Sample preservation following chilled ethanol protocols on average resulted in higher sequencing success (88.9%) and barcode compliance (81.8%) rates in comparison with nonchilled sample preservation (79.9% and 71.3% respectively). These results confirm previous observations by Prosser et al. (2013) for crustaceans and Post, Flook, and Millest (1993) for insects, who demonstrated improved sequence recovery when chilled ethanol was used to preserve specimens. This method should be seriously considered when attempting to work with groups that historically show low amplification success, such as Crustacea (Elías‐Gutiérrez, Martínez‐Jerónimo, et al., 2008; Hirai et al., 2013; Jeffery et al., 2011; Prosser et al., 2013). However, when controlling for geographic region (temperate vs. tropical) *betadisper* analyses suggested some variability with decreased sequencing success in the temperate samples. Another factor that could affect sequencing success rates is the presence of green algae blooms in sample sites. During the course of this study, we noticed that sequencing success from specimens taken from the same location (Guelph Lake) varied depending on blooms of green algae. If green algae were abundant and the samples were not immediately placed on ice after fixation, sequencing success dropped to only 30% (Appendix S2), perhaps due to PCR inhibitors released from the algae. This could explain the variable sequencing success in barcode recovery found in temperate samples.

The use of chilled ethanol for specimen preservation is important for improving sequence recovery rates. For example, previous studies on Macrothricidae cladocerans saw an improvement of sequencing success to 80% from 50% by utilizing the same cold storage preservation method described in Prosser et al. (2013). More specifically, *Holopedium* (Cladocera) preserved in ethanol at ambient temperature completely failed to amplify COI (Jeffery et al., 2011), but sequence recovery was high when liquid nitrogen was used as a preservative (Rowe, Adamowicz, & Hebert, 2007). Furthermore, samples from Guelph Lake initially preserved in chilled ethanol remained darker after fixation in comparison with the sample kept at room temperature, which appeared clearer after fixation (Appendix S3a,b). We detected a similar contrast in color of samples from Bacalar Lake despite the near absence of green algae from this habitat (Appendix S3c,d); however, no apparent effect on sequence recovery was observed. Additional sampling would be required to further investigate this observation.

It was also noted that for some calanoids, the dorsal muscles kept their fibrous consistency when preserved in chilled ethanol but were often fragmented when fixed in room temperature ethanol (Appendix S3e,f). As muscle tissue is the primary source for mitochondria rich tissue, its proper preservation may significantly impact COI sequence recovery rates.

To a lesser extent, the use of nondenatured ethanol can also improve sequence recovery rates for freshwater zooplankton, especially the crustaceans (Table 3) could be affected by the presence of methanol and isopropanol in denatured ethanol. Previously, Post et al. (1993) demonstrated that methanol and propanol result in highly degraded DNA. However, we recognize that pure ethanol can be difficult to procure in several countries.

### 4.5 General conclusions

The main impediment to barcode recovery from most groups of zooplankton is variation in the initial fixation of samples rather than failures in primer binding. It is likely that standard preservation methods which involve storage at ambient temperatures lead to rapid DNA degradation in either the specimens or in algae that are in the environment or the gut tract. Apparently, a short period of cold storage immediately following fixation in alcohol halts this degradation, by allowing the fixative to penetrate deeper tissues. In some groups like rotifers and molluscs, we reached 100% success but our taxonomic coverage was limited. Although the importance of preservation at low temperatures has been suggested (Prosser et al., 2013), most laboratories still report difficulties in DNA barcode recovery from zooplankton, while failing to adopt low temperature preservation methods. It is for this reason that we reemphasize the critical importance of low temperature preservation and a removal of all water from the sample immediately after collection.

Although the taxonomic impediment remains a barrier, as many of the BINs detected in this study remain unidentified, it is important to connect taxonomic knowledge with DNA barcodes to build the library of reference sequences held in BOLD, and integrate current knowledge of the biology and ecology of the organisms.

We are currently working with a multidisciplinary group to obtain morphological identifications for the majority of the taxa in this study. We expect many of the BINs to be new species for science, increasing the value and need of conservation of these fragile aquatic systems. However, there is a high need for more effort to build DNA barcode reference libraries for all Mexican fauna. Mexico is considered the fourth most biodiversity‐rich region in the world (CONABIO, 2008), so this attempt should not be limited just to zooplankton but all other aquatic taxa as well.

This holistic view will make it possible to better understand the response of freshwater ecosystems to human activity, global warming, or the introduction of alien species. In the past, most aquatic biomonitoring studies assessed the incidence of indicator species (e.g., just chironomids, as Odume, Palmer, Arimoro, & Mensah, 2016 suggested), but not the whole community. Chironomids, like mites, are good indicators of water quality (Odume et al., 2016), and DNA barcoding has greatly improved their identification in the Baltic Sea (Brodin, Ejdung, Strandberg, & Lyrholm, 2013). However, the use of DNA barcodes in combination with light traps and improved specimen preservation makes it possible also to assess the entire zooplankton community.

Finally, we consider that construction of a reliable baseline is a critical first step leading to biomonitoring based on more accurate methods. For example, the results after the use of the methodologies proposed here in conjunction with taxonomic expertise will accelerate the construction of the reference libraries and baseline data, allowing a near future rapid development of well‐supported strategies for conservation and management based on new technologies like next‐generation sequencing.

## CONFLICT OF INTEREST

None declared.

## AUTHOR CONTRIBUTIONS

M.E.G., M.V.M., and J.A.C.C. designed the field methods and samplings. M.E.G., M.V.M., J.T., and M.R.Y. wrote the manuscript.

## DATA ACCESSIBILITY

BOLD Dataset: Freshwater zooplankton from light traps (DS‐LTZPL).

GenBank Accessions: MG448608 ‐ MG450325.
