# Supplementary material for: Improved protocols to accelerate the assembly of DNA barcode reference libraries for freshwater zooplankton
Source: Ecol Evol. 2018 Feb 15;8(5):3002–18. doi: 10.1002/ece3.3742 (PMC5838060; doi:10.1002/ece3.3742)
Supplement: Supplementary file 1 [file ECE3-8-3002-s001.pdf]

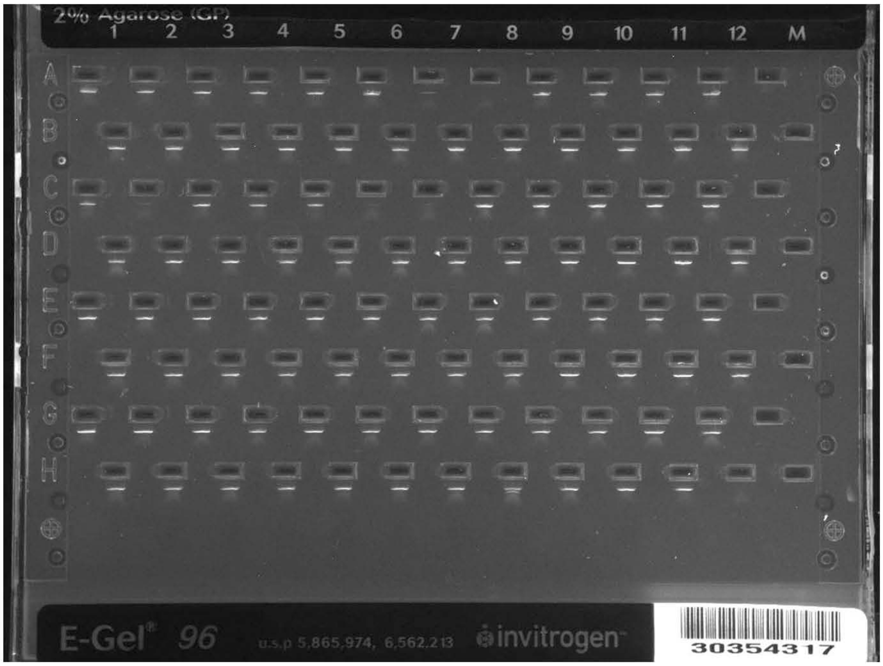

A

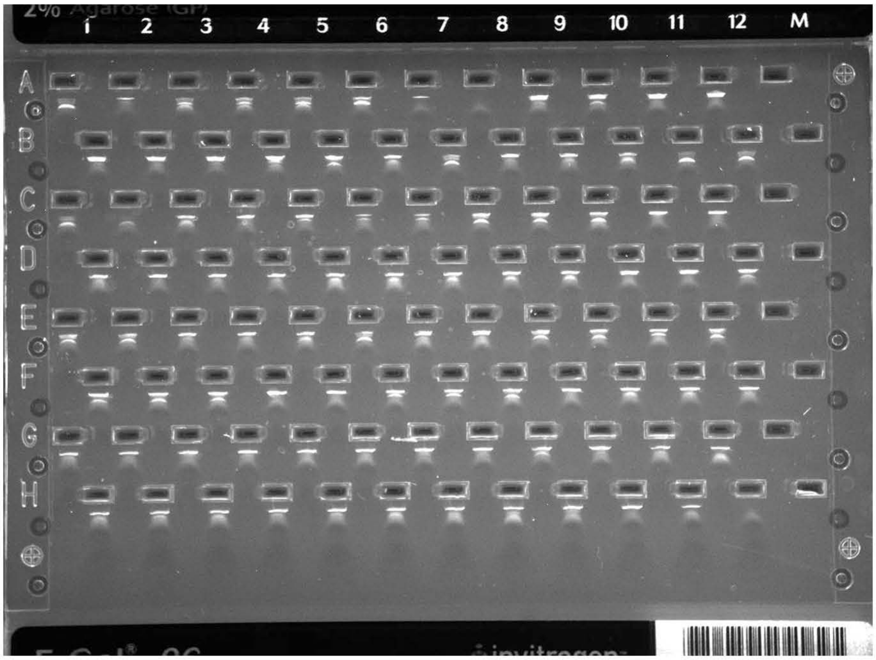

B

Appendix S1.- A) PCR products of fish from Bacalar Lake using C_Fish cocktail.
B.- PCR products using the Zplank primers with the same plate
