# Supplementary material for: Improved protocols to accelerate the assembly of DNA barcode reference libraries for freshwater zooplankton
Source: Ecol Evol. 2018 Feb 15;8(5):3002–18. doi: 10.1002/ece3.3742 (PMC5838060; doi:10.1002/ece3.3742)
Supplement: Supplementary file 3 [file ECE3-8-3002-s003.pdf]

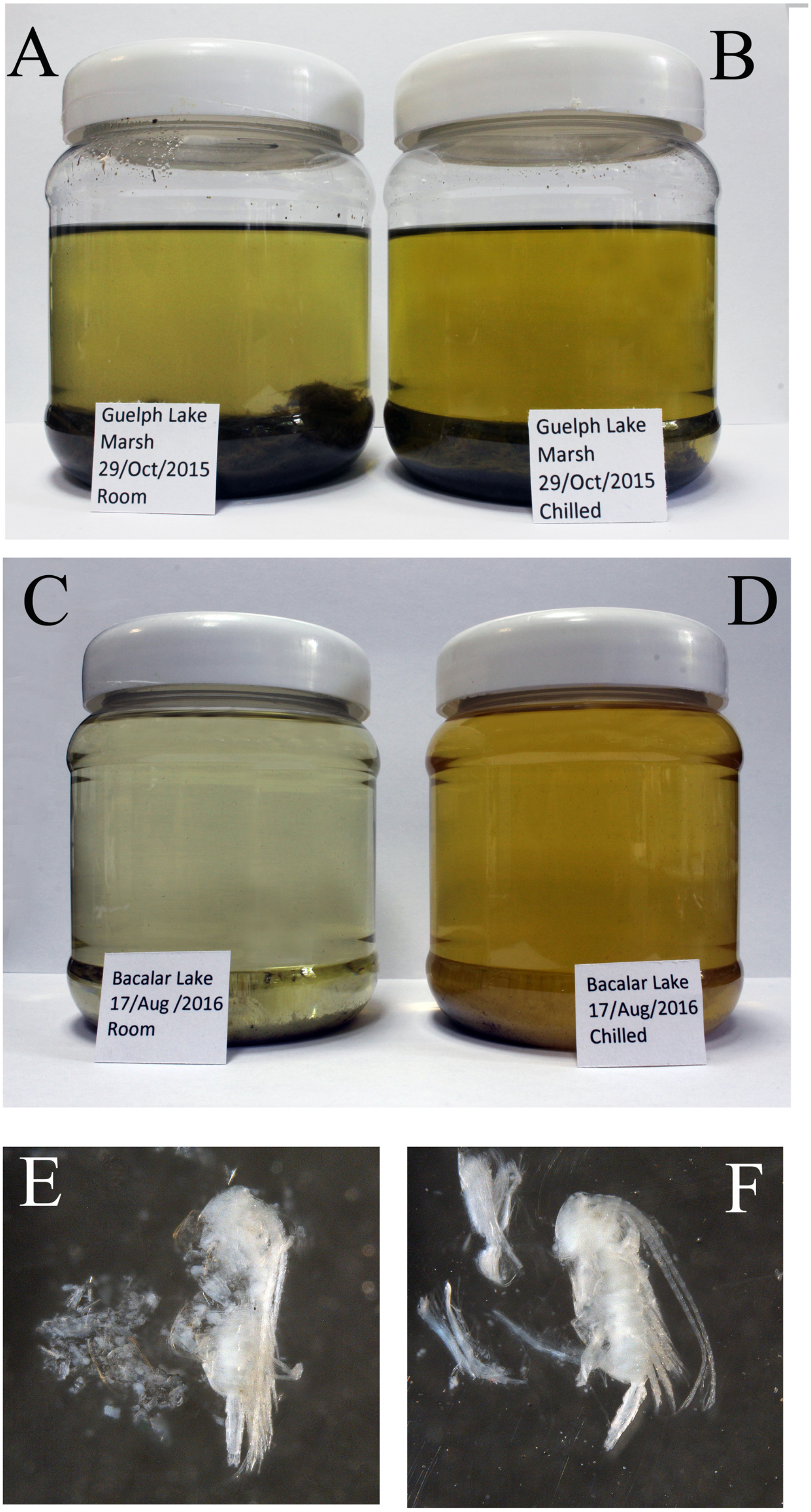

Appendix S3.- A) Sample from Guelph Lake fixed with non-chilled ethanol, collected in October 29, 2015; B) Same sample, fixed with chilled ethanol. Photo taken after 18 months of storage at room temperature; C) Sample from Bacalar Lake collected in August 17, 2016 fixed with non-chilled ethanol; D) Same sample fixed with chilled ethanol; E) Dissected calanoid with dorsal muscles from cephalothorax, fixed with non-chilled ethanol; F) Specimen from the same sample, fixed with chilled ethanol.
