# Supplementary material for: Improved protocols to accelerate the assembly of DNA barcode reference libraries for freshwater zooplankton
Source: Ecol Evol. 2018 Feb 15;8(5):3002–18. doi: 10.1002/ece3.3742 (PMC5838060; doi:10.1002/ece3.3742)
Supplement: Supplementary file 4 [file ECE3-8-3002-s004.pdf]

BOLD TaxonID Tree

Rotifera

Title	:	Tree Result - Search (27 records)
Date	:	10-Mar-2017
Data Type	:	Nucleotide
Distance Model	:	Kimura 2 Parameter
Marker	:	COI-5P
Colourization	:	[blue]=Stop Codons [red]=Contamination or misidentification

Label	:	Process ID
Label	:	Taxon
Label	:	Exact Site
Label	:	Barcode Cluster (BIN)

Sequence Count	:	27
Species count	:	3
Genus count	:	5
Family count	:	4
Unidentified	:	20

BIN Count	:	11

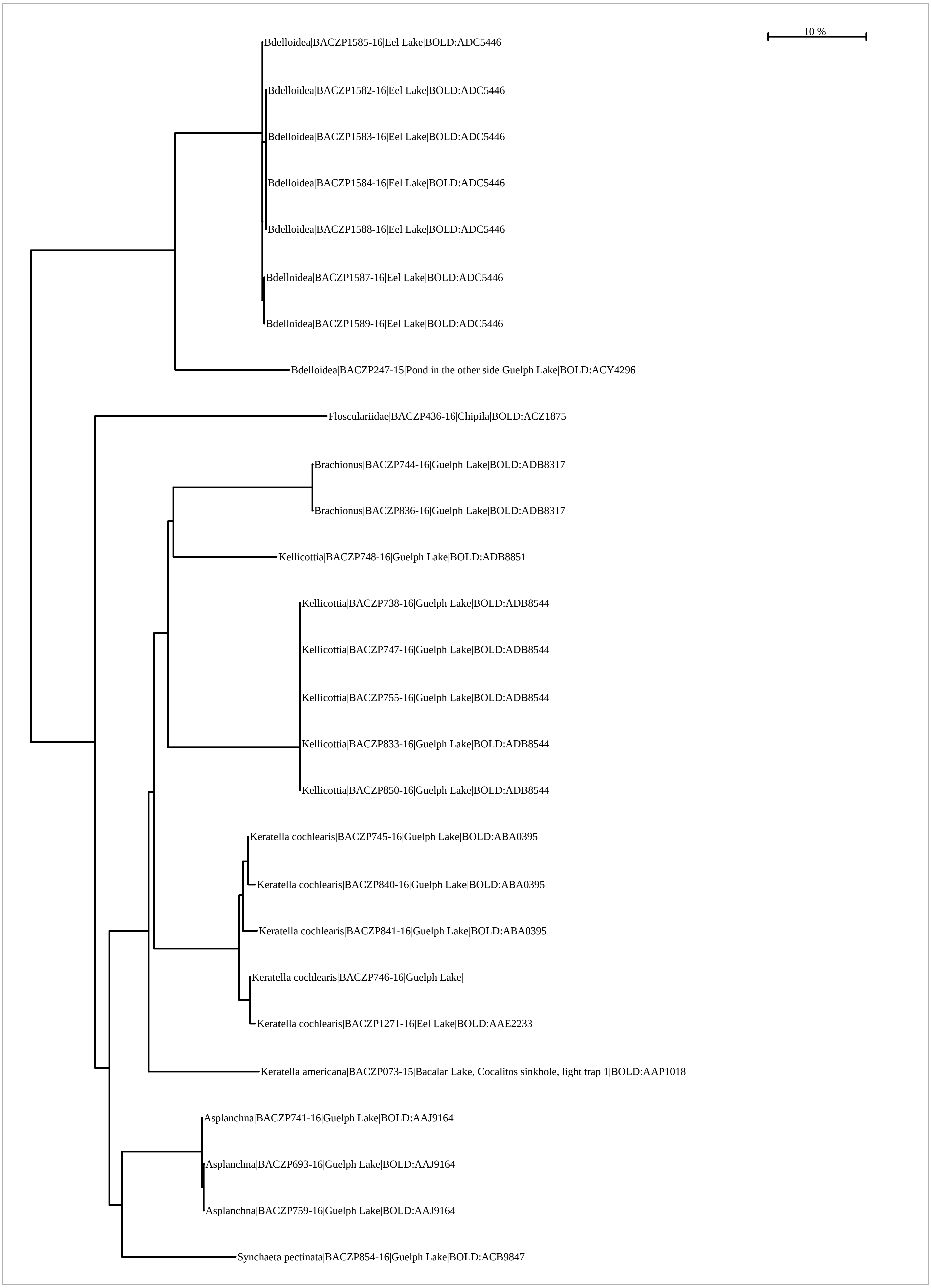
10 %
Bdelloidea|BACZP1585-16|Eel Lake|BOLD:ADC5446
Bdelloidea|BACZP1582-16|Eel Lake|BOLD:ADC5446
Bdelloidea|BACZP1583-16|Eel Lake|BOLD:ADC5446
Bdelloidea|BACZP1584-16|Eel Lake|BOLD:ADC5446
Bdelloidea|BACZP1588-16|Eel Lake|BOLD:ADC5446
Bdelloidea|BACZP1587-16|Eel Lake|BOLD:ADC5446
Bdelloidea|BACZP1589-16|Eel Lake|BOLD:ADC5446
Bdelloidea|BACZP247-15|Pond in the other side Guelph Lake|BOLD:ACY4296
Floscularidae|BACZP436-16|Chipila|BOLD:ACZ1875
Brachionus|BACZP744-16|Guelph Lake|BOLD:ADB8317
Brachionus|BACZP836-16|Guelph Lake|BOLD:ADB8317
Kellicottia|BACZP748-16|Guelph Lake|BOLD:ADB8851
Kellicottia|BACZP738-16|Guelph Lake|BOLD:ADB8544
Kellicottia|BACZP747-16|Guelph Lake|BOLD:ADB8544
Kellicottia|BACZP755-16|Guelph Lake|BOLD:ADB8544
Kellicottia|BACZP833-16|Guelph Lake|BOLD:ADB8544
Kellicottia|BACZP850-16|Guelph Lake|BOLD:ADB8544
Keratella cochlearis|BACZP745-16|Guelph Lake|BOLD:ABA0395
Keratella cochlearis|BACZP840-16|Guelph Lake|BOLD:ABA0395
Keratella cochlearis|BACZP841-16|Guelph Lake|BOLD:ABA0395
Keratella cochlearis|BACZP746-16|Guelph Lake|
Keratella cochlearis|BACZP1271-16|Eel Lake|BOLD:AAE2233
Keratella americana|BACZP073-15|Bacalar Lake, Cocalitos sinkhole, light trap 1|BOLD:AAP1018
Asplanchna|BACZP741-16|Guelph Lake|BOLD:AAJ9164
Asplanchna|BACZP693-16|Guelph Lake|BOLD:AAJ9164
Asplanchna|BACZP759-16|Guelph Lake|BOLD:AAJ9164
Synchaeta pectinata|BACZP854-16|Guelph Lake|BOLD:ACB9847

BOLD TaxonID Tree

Mollusca

Title : Tree Result - Search (9 records)

Date : 10-Mar-2017

Data Type : Nucleotide

Distance Model : Kimura 2 Parameter

Marker : COI-5P

Colourization : [blue]=Stop Codons [red]=Contamination or misidentification

Label : Process ID

Label : Taxon

Label : Exact Site

Label : Barcode Cluster (BIN)

Sequence Count : 9

Species count : 0

Genus count : 1

Family count : 2

Unidentified : 9

BIN Count : 3

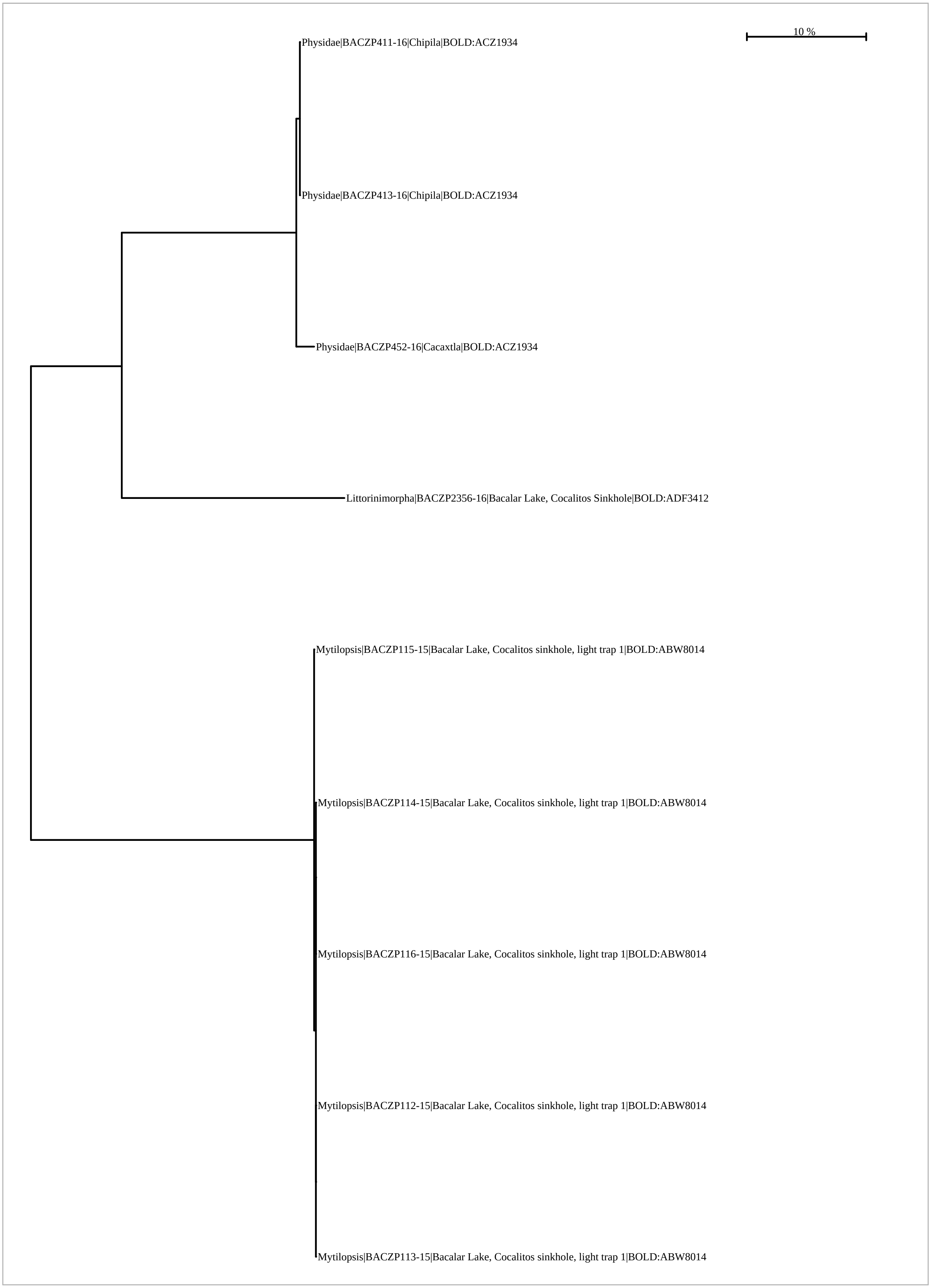
10 %
Physidae|BACZP411-16|Chipila|BOLD:ACZ1934
Physidae|BACZP413-16|Chipila|BOLD:ACZ1934
Physidae|BACZP452-16|Cacaxtla|BOLD:ACZ1934
Littorinimorpha|BACZP2356-16|Bacalar Lake, Cocalitos Sinkhole|BOLD:ADF3412
Mytilopsis|BACZP115-15|Bacalar Lake, Cocalitos sinkhole, light trap 1|BOLD:ABW8014
Mytilopsis|BACZP114-15|Bacalar Lake, Cocalitos sinkhole, light trap 1|BOLD:ABW8014
Mytilopsis|BACZP116-15|Bacalar Lake, Cocalitos sinkhole, light trap 1|BOLD:ABW8014
Mytilopsis|BACZP112-15|Bacalar Lake, Cocalitos sinkhole, light trap 1|BOLD:ABW8014
Mytilopsis|BACZP113-15|Bacalar Lake, Cocalitos sinkhole, light trap 1|BOLD:ABW8014

BOLD TaxonID Tree

Arachnida

Title	:	SEARCH: Sample ids(407 ids) [SEARCH3]
Date	:	10-March-2017
Data Type	:	Nucleotide
Distance Model	:	Kimura 2 Parameter
Marker	:	COI-5P
Codon Positions	:	1st, 2nd, 3rd
Labels	:	Site, ProcessID, BIN uri
Filters	:	Length > 200
Colorization	:	[blue]=Stop Codons [red]=Contamination or misidentification

Sequence Count	:	366
Species count	:	2
Genus count	:	9
Family count	:	10
Unidentified	:	364

BIN Count	:	59

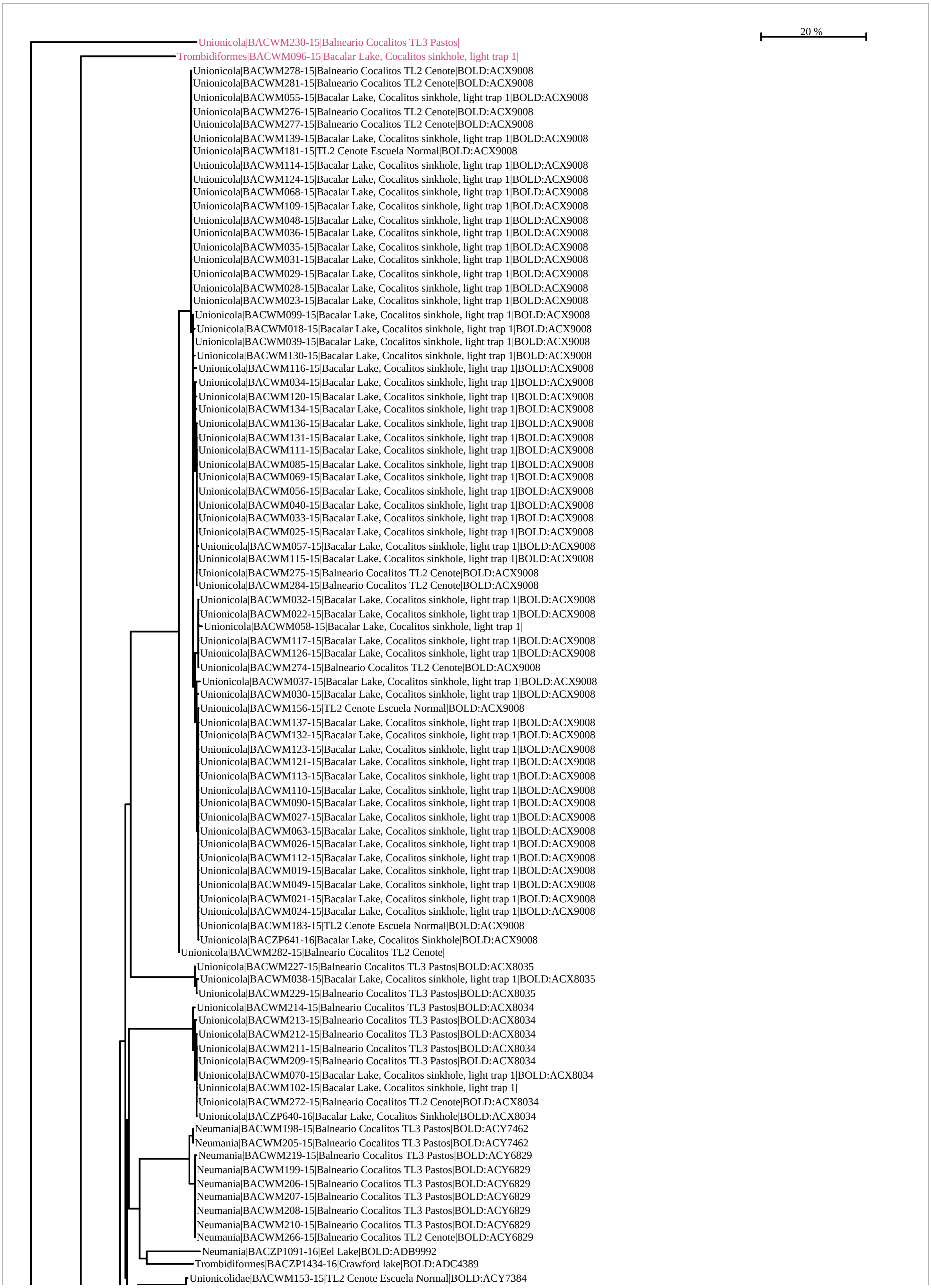
20 %
Unionicola|BACWM230-15|Balneario Cocalitos TL3 Pastos|
Trombidiformes|BACWM096-15|Bacalar Lake, Cocalitos sinkhole, light trap 1|
Unionicola|BACWM278-15|Balneario Cocalitos TL2 Cenote|BOLD:ACX9008
Unionicola|BACWM281-15|Balneario Cocalitos TL2 Cenote|BOLD:ACX9008
Unionicola|BACWM055-15|Bacalar Lake, Cocalitos sinkhole, light trap 1|BOLD:ACX9008
Unionicola|BACWM276-15|Balneario Cocalitos TL2 Cenote|BOLD:ACX9008
Unionicola|BACWM277-15|Balneario Cocalitos TL2 Cenote|BOLD:ACX9008
Unionicola|BACWM139-15|Bacalar Lake, Cocalitos sinkhole, light trap 1|BOLD:ACX9008
Unionicola|BACWM181-15|TL2 Cenote Escuela Normal|BOLD:ACX9008
Unionicola|BACWM114-15|Bacalar Lake, Cocalitos sinkhole, light trap 1|BOLD:ACX9008
Unionicola|BACWM124-15|Bacalar Lake, Cocalitos sinkhole, light trap 1|BOLD:ACX9008
Unionicola|BACWM068-15|Bacalar Lake, Cocalitos sinkhole, light trap 1|BOLD:ACX9008
Unionicola|BACWM109-15|Bacalar Lake, Cocalitos sinkhole, light trap 1|BOLD:ACX9008
Unionicola|BACWM048-15|Bacalar Lake, Cocalitos sinkhole, light trap 1|BOLD:ACX9008
Unionicola|BACWM036-15|Bacalar Lake, Cocalitos sinkhole, light trap 1|BOLD:ACX9008
Unionicola|BACWM035-15|Bacalar Lake, Cocalitos sinkhole, light trap 1|BOLD:ACX9008
Unionicola|BACWM031-15|Bacalar Lake, Cocalitos sinkhole, light trap 1|BOLD:ACX9008
Unionicola|BACWM029-15|Bacalar Lake, Cocalitos sinkhole, light trap 1|BOLD:ACX9008
Unionicola|BACWM028-15|Bacalar Lake, Cocalitos sinkhole, light trap 1|BOLD:ACX9008
Unionicola|BACWM023-15|Bacalar Lake, Cocalitos sinkhole, light trap 1|BOLD:ACX9008
Unionicola|BACWM099-15|Bacalar Lake, Cocalitos sinkhole, light trap 1|BOLD:ACX9008
Unionicola|BACWM018-15|Bacalar Lake, Cocalitos sinkhole, light trap 1|BOLD:ACX9008
Unionicola|BACWM039-15|Bacalar Lake, Cocalitos sinkhole, light trap 1|BOLD:ACX9008
Unionicola|BACWM130-15|Bacalar Lake, Cocalitos sinkhole, light trap 1|BOLD:ACX9008
Unionicola|BACWM116-15|Bacalar Lake, Cocalitos sinkhole, light trap 1|BOLD:ACX9008
Unionicola|BACWM034-15|Bacalar Lake, Cocalitos sinkhole, light trap 1|BOLD:ACX9008
Unionicola|BACWM120-15|Bacalar Lake, Cocalitos sinkhole, light trap 1|BOLD:ACX9008
Unionicola|BACWM134-15|Bacalar Lake, Cocalitos sinkhole, light trap 1|BOLD:ACX9008
Unionicola|BACWM136-15|Bacalar Lake, Cocalitos sinkhole, light trap 1|BOLD:ACX9008
Unionicola|BACWM131-15|Bacalar Lake, Cocalitos sinkhole, light trap 1|BOLD:ACX9008
Unionicola|BACWM111-15|Bacalar Lake, Cocalitos sinkhole, light trap 1|BOLD:ACX9008
Unionicola|BACWM085-15|Bacalar Lake, Cocalitos sinkhole, light trap 1|BOLD:ACX9008
Unionicola|BACWM069-15|Bacalar Lake, Cocalitos sinkhole, light trap 1|BOLD:ACX9008
Unionicola|BACWM056-15|Bacalar Lake, Cocalitos sinkhole, light trap 1|BOLD:ACX9008
Unionicola|BACWM040-15|Bacalar Lake, Cocalitos sinkhole, light trap 1|BOLD:ACX9008
Unionicola|BACWM033-15|Bacalar Lake, Cocalitos sinkhole, light trap 1|BOLD:ACX9008
Unionicola|BACWM025-15|Bacalar Lake, Cocalitos sinkhole, light trap 1|BOLD:ACX9008
Unionicola|BACWM057-15|Bacalar Lake, Cocalitos sinkhole, light trap 1|BOLD:ACX9008
Unionicola|BACWM115-15|Bacalar Lake, Cocalitos sinkhole, light trap 1|BOLD:ACX9008
Unionicola|BACWM275-15|Balneario Cocalitos TL2 Cenote|BOLD:ACX9008
Unionicola|BACWM284-15|Balneario Cocalitos TL2 Cenote|BOLD:ACX9008
Unionicola|BACWM032-15|Bacalar Lake, Cocalitos sinkhole, light trap 1|BOLD:ACX9008
Unionicola|BACWM022-15|Bacalar Lake, Cocalitos sinkhole, light trap 1|BOLD:ACX9008
Unionicola|BACWM058-15|Bacalar Lake, Cocalitos sinkhole, light trap 1|
Unionicola|BACWM117-15|Bacalar Lake, Cocalitos sinkhole, light trap 1|BOLD:ACX9008
Unionicola|BACWM126-15|Bacalar Lake, Cocalitos sinkhole, light trap 1|BOLD:ACX9008
Unionicola|BACWM274-15|Balneario Cocalitos TL2 Cenote|BOLD:ACX9008
Unionicola|BACWM037-15|Bacalar Lake, Cocalitos sinkhole, light trap 1|BOLD:ACX9008
Unionicola|BACWM030-15|Bacalar Lake, Cocalitos sinkhole, light trap 1|BOLD:ACX9008
Unionicola|BACWM156-15|TL2 Cenote Escuela Normal|BOLD:ACX9008
Unionicola|BACWM137-15|Bacalar Lake, Cocalitos sinkhole, light trap 1|BOLD:ACX9008
Unionicola|BACWM132-15|Bacalar Lake, Cocalitos sinkhole, light trap 1|BOLD:ACX9008
Unionicola|BACWM123-15|Bacalar Lake, Cocalitos sinkhole, light trap 1|BOLD:ACX9008
Unionicola|BACWM121-15|Bacalar Lake, Cocalitos sinkhole, light trap 1|BOLD:ACX9008
Unionicola|BACWM113-15|Bacalar Lake, Cocalitos sinkhole, light trap 1|BOLD:ACX9008
Unionicola|BACWM110-15|Bacalar Lake, Cocalitos sinkhole, light trap 1|BOLD:ACX9008
Unionicola|BACWM090-15|Bacalar Lake, Cocalitos sinkhole, light trap 1|BOLD:ACX9008
Unionicola|BACWM027-15|Bacalar Lake, Cocalitos sinkhole, light trap 1|BOLD:ACX9008
Unionicola|BACWM063-15|Bacalar Lake, Cocalitos sinkhole, light trap 1|BOLD:ACX9008
Unionicola|BACWM026-15|Bacalar Lake, Cocalitos sinkhole, light trap 1|BOLD:ACX9008
Unionicola|BACWM112-15|Bacalar Lake, Cocalitos sinkhole, light trap 1|BOLD:ACX9008
Unionicola|BACWM019-15|Bacalar Lake, Cocalitos sinkhole, light trap 1|BOLD:ACX9008
Unionicola|BACWM049-15|Bacalar Lake, Cocalitos sinkhole, light trap 1|BOLD:ACX9008
Unionicola|BACWM021-15|Bacalar Lake, Cocalitos sinkhole, light trap 1|BOLD:ACX9008
Unionicola|BACWM024-15|Bacalar Lake, Cocalitos sinkhole, light trap 1|BOLD:ACX9008
Unionicola|BACWM183-15|TL2 Cenote Escuela Normal|BOLD:ACX9008
Unionicola|BACZP641-16|Bacalar Lake, Cocalitos Sinkhole|BOLD:ACX9008
Unionicola|BACWM282-15|Balneario Cocalitos TL2 Cenote|
Unionicola|BACWM227-15|Balneario Cocalitos TL3 Pastos|BOLD:ACX8035
Unionicola|BACWM038-15|Bacalar Lake, Cocalitos sinkhole, light trap 1|BOLD:ACX8035
Unionicola|BACWM229-15|Balneario Cocalitos TL3 Pastos|BOLD:ACX8035
Unionicola|BACWM214-15|Balneario Cocalitos TL3 Pastos|BOLD:ACX8034
Unionicola|BACWM213-15|Balneario Cocalitos TL3 Pastos|BOLD:ACX8034
Unionicola|BACWM212-15|Balneario Cocalitos TL3 Pastos|BOLD:ACX8034
Unionicola|BACWM211-15|Balneario Cocalitos TL3 Pastos|BOLD:ACX8034
Unionicola|BACWM209-15|Balneario Cocalitos TL3 Pastos|BOLD:ACX8034
Unionicola|BACWM070-15|Bacalar Lake, Cocalitos sinkhole, light trap 1|BOLD:ACX8034
Unionicola|BACWM102-15|Bacalar Lake, Cocalitos sinkhole, light trap 1|
Unionicola|BACWM272-15|Balneario Cocalitos TL2 Cenote|BOLD:ACX8034
Unionicola|BACZP640-16|Bacalar Lake, Cocalitos Sinkhole|BOLD:ACX8034
Neumania|BACWM198-15|Balneario Cocalitos TL3 Pastos|BOLD:ACY7462
Neumania|BACWM205-15|Balneario Cocalitos TL3 Pastos|BOLD:ACY7462
Neumania|BACWM219-15|Balneario Cocalitos TL3 Pastos|BOLD:ACY6829
Neumania|BACWM199-15|Balneario Cocalitos TL3 Pastos|BOLD:ACY6829
Neumania|BACWM206-15|Balneario Cocalitos TL3 Pastos|BOLD:ACY6829
Neumania|BACWM207-15|Balneario Cocalitos TL3 Pastos|BOLD:ACY6829
Neumania|BACWM208-15|Balneario Cocalitos TL3 Pastos|BOLD:ACY6829
Neumania|BACWM210-15|Balneario Cocalitos TL3 Pastos|BOLD:ACY6829
Neumania|BACWM266-15|Balneario Cocalitos TL2 Cenote|BOLD:ACY6829
Neumania|BACZP1091-16|Eel Lake|BOLD:ADB9992
Trombidiformes|BACZP1434-16|Crawford lake|BOLD:ADC4389
Unionicolidae|BACWM153-15|TL2 Cenote Escuela Normal|BOLD:ACY7384

Neumania|BACZP1091-16|Eel Lake|BOLD:ADB9992
Trombidiformes|BACZP1434-16|Crawford lake|BOLD:ADC4389
Unionicolidae|BACWM153-15|TL2 Cenote Escuela Normal|BOLD:ACY7384
Unionicolidae|BACWM258-15|Balneario Cocalitos TL2 Cenote|BOLD:ACY7384
Unionicolidae|BACWM253-15|Balneario Cocalitos TL2 Cenote|BOLD:ACY7381
Unionicolidae|BACWM201-15|Balneario Cocalitos TL3 Pastos|BOLD:ACY7381
Unionicolidae|BACWM249-15|Balneario Cocalitos TL2 Cenote|BOLD:ACY7381
Unionicolidae|BACWM254-15|Balneario Cocalitos TL2 Cenote|BOLD:ACY7381
Unionicolidae|BACWM261-15|Balneario Cocalitos TL2 Cenote|BOLD:ACY7381
Unionicolidae|BACWM200-15|Balneario Cocalitos TL3 Pastos|BOLD:ACY7381
Unionicolidae|BACWM204-15|Balneario Cocalitos TL3 Pastos|BOLD:ACY7381
Unionicolidae|BACWM203-15|Balneario Cocalitos TL3 Pastos|BOLD:ACY7381
Unionicolidae|BACWM202-15|Balneario Cocalitos TL3 Pastos|BOLD:ACY7381
Unionicolidae|BACWM248-15|Balneario Cocalitos TL2 Cenote|BOLD:ACY7381
Unionicolidae|BACWM250-15|Balneario Cocalitos TL2 Cenote|BOLD:ACY7381
Unionicolidae|BACWM251-15|Balneario Cocalitos TL2 Cenote|BOLD:ACY7381
Unionicolidae|BACWM256-15|Balneario Cocalitos TL2 Cenote|BOLD:ACY7381
Unionicolidae|BACWM260-15|Balneario Cocalitos TL2 Cenote|BOLD:ACY7381
Unionicolidae|BACWM264-15|Balneario Cocalitos TL2 Cenote|BOLD:ACY7381
Trombidiformes|BACZP1467-16|Crawford lake|BOLD:ACL6104
Trombidiformes|BACZP1488-16|Crawford lake, vegetation|BOLD:ACI6935
Trombidiformes|BACZP1479-16|Crawford lake|BOLD:ACJ8639
Trombidiformes|BACZP1503-16|Crawford lake, vegetation|BOLD:ACJ8639
Unionicolidae|BACZP1522-16|Eel Lake|BOLD:ADC5530
Unionicolidae|BACZP1401-16|Crawford lake|BOLD:ADB9993
Unionicolidae|BACZP1286-16|Eel Lake|BOLD:ADB9993
Unionicolidae|BACZP1287-16|Eel Lake|BOLD:ADB9993
Unionicolidae|BACZP1525-16|Eel Lake|BOLD:ADB9993
Unionicolidae|BACZP1402-16|Crawford lake|BOLD:ADC1781
Unionicolidae|BACZP1529-16|Eel Lake|BOLD:ADC3982
Unionicola|BACWM087-15|Bacalar Lake, Cocalitos sinkhole, light trap 1|BOLD:ACX9009
Unionicola|BACWM064-15|Bacalar Lake, Cocalitos sinkhole, light trap 1|BOLD:ACX9009
Unionicola|BACWM084-15|Bacalar Lake, Cocalitos sinkhole, light trap 1|BOLD:ACX9009
Unionicola|BACWM091-15|Bacalar Lake, Cocalitos sinkhole, light trap 1|
Unionicola|BACZP2198-16|Bacalar Lake, Cocalitos Sinkhole|BOLD:ACX9009
Unionicola|BACZP2386-16|Bacalar Lake, Cocalitos Sinkhole|BOLD:ACX9009
Unionicola|BACZP2199-16|Bacalar Lake, Cocalitos Sinkhole|BOLD:ACX9009
Unionicola|BACZP2379-16|Bacalar Lake, Cocalitos Sinkhole|BOLD:ACX9009
Unionicola|BACWM118-15|Bacalar Lake, Cocalitos sinkhole, light trap 1|BOLD:ACX9009
Unionicola|BACWM180-15|TL2 Cenote Escuela Normal|BOLD:ACX9009
Unionicola|BACZP638-16|Bacalar Lake, Cocalitos Sinkhole|BOLD:ACX9009
Unionicola|BACWM095-15|Bacalar Lake, Cocalitos sinkhole, light trap 1|BOLD:ACX9009
Unionicola|BACWM050-15|Bacalar Lake, Cocalitos sinkhole, light trap 1|BOLD:ACX9009
Unionicola|BACWM094-15|Bacalar Lake, Cocalitos sinkhole, light trap 1|BOLD:ACX9009
Unionicola|BACWM174-15|TL2 Cenote Escuela Normal|BOLD:ACX9009
Unionicola|BACZP2392-16|Bacalar Lake, Cocalitos Sinkhole|BOLD:ACX9009
Unionicola|BACZP2394-16|Bacalar Lake, Cocalitos Sinkhole|BOLD:ACX9009
Unionicola|BACZP2196-16|Bacalar Lake, Cocalitos Sinkhole|BOLD:ACX9009
Unionicola|BACZP2334-16|Bacalar Lake, Cocalitos Sinkhole|BOLD:ACX9009
Unionicola|BACWM178-15|TL2 Cenote Escuela Normal|BOLD:ACX9009
Unionicola|BACZP2200-16|Bacalar Lake, Cocalitos Sinkhole|BOLD:ACX9009
Unionicola|BACWM176-15|TL2 Cenote Escuela Normal|BOLD:ACX9009
Trombidiformes|BACZP2327-16|Bacalar Lake, Cocalitos Sinkhole|BOLD:ACX9009
Unionicola|BACWM170-15|TL2 Cenote Escuela Normal|BOLD:ACX9009
Unionicola|BACWM171-15|TL2 Cenote Escuela Normal|BOLD:ACX9009
Unionicola|BACWM172-15|TL2 Cenote Escuela Normal|BOLD:ACX9009
Unionicola|BACWM177-15|TL2 Cenote Escuela Normal|BOLD:ACX9009
Unionicola|BACWM179-15|TL2 Cenote Escuela Normal|BOLD:ACX9009
Unionicola|BACWM182-15|TL2 Cenote Escuela Normal|BOLD:ACX9009
Unionicola|BACWM184-15|TL2 Cenote Escuela Normal|BOLD:ACX9009
Unionicola|BACZP901-16|Bacalar Lake, Cocalitos Sinkhole|BOLD:ACX9009
Unionicola|BACZP2395-16|Bacalar Lake, Cocalitos Sinkhole|BOLD:ACX9009
Trombidiformes|BACZP2329-16|Bacalar Lake, Cocalitos Sinkhole|BOLD:ACX9009
Unionicola|BACWM280-15|Balneario Cocalitos TL2 Cenote|BOLD:ACX9009
Unionicola|BACWM089-15|Bacalar Lake, Cocalitos sinkhole, light trap 1|BOLD:ACX9009
Unionicola|BACWM285-15|Balneario Cocalitos TL2 Cenote|BOLD:ACX9009
Unionicola|BACWM175-15|TL2 Cenote Escuela Normal|BOLD:ACX9009
Unionicola|BACWM093-15|Bacalar Lake, Cocalitos sinkhole, light trap 1|BOLD:ACX9009
Unionicola|BACWM086-15|Bacalar Lake, Cocalitos sinkhole, light trap 1|BOLD:ACX9009
Unionicola|BACWM097-15|Bacalar Lake, Cocalitos sinkhole, light trap 1|BOLD:ACX9009
Unionicola|BACWM061-15|Bacalar Lake, Cocalitos sinkhole, light trap 1|BOLD:ACX9009
Unionicola|BACWM119-15|Bacalar Lake, Cocalitos sinkhole, light trap 1|BOLD:ACX9009
Unionicola|BACWM279-15|Balneario Cocalitos TL2 Cenote|BOLD:ACX9009
Unionicola|BACZP637-16|Bacalar Lake, Cocalitos Sinkhole|BOLD:ACX9009
Unionicola|BACZP639-16|Bacalar Lake, Cocalitos Sinkhole|BOLD:ACX9009
Unionicola|BACZP900-16|Bacalar Lake, Cocalitos Sinkhole|BOLD:ACX9009
Unionicola|BACZP2366-16|Bacalar Lake, Cocalitos Sinkhole|BOLD:ACX9009
Unionicola|BACZP2221-16|Bacalar Lake, Cocalitos Sinkhole|BOLD:ACX9009
Arrenurus|BACWM186-15|Balneario Cocalitos TL3 Pastos|BOLD:ACY6809
Arrenurus|BACWM185-15|Balneario Cocalitos TL3 Pastos|BOLD:ACY6809
Arrenurus|BACWM231-15|Balneario Cocalitos TL3 Pastos|BOLD:ACY6809
Arrenurus|BACWM013-15|Bacalar Lake, Cocalitos sinkhole, light trap 1|BOLD:ACX8462
Arrenurus|BACWM187-15|Balneario Cocalitos TL3 Pastos|BOLD:ACX8462
Arrenurus|BACWM072-15|Bacalar Lake, Cocalitos sinkhole, light trap 1|BOLD:ACX8788
Arrenurus|BACWM150-15|TL2 Cenote Escuela Normal|BOLD:ACX8788
Arrenurus apetiolatus|BACZP1279-16|Eel Lake|BOLD:AAJ3154
Arrenurus|BACZP864-16|Bacalar Lake, Cocalitos Sinkhole|BOLD:ACX8789
Arrenurus|BACWM041-15|Bacalar Lake, Cocalitos sinkhole, light trap 1|BOLD:ACX8789
Arrenurus|BACZP2233-16|Bacalar Lake, Cocalitos Sinkhole|BOLD:ACX8789
Arrenurus|BACWM192-15|Balneario Cocalitos TL3 Pastos|BOLD:ACL2418
Arrenurus|BACWM008-15|Bacalar Lake, Cocalitos sinkhole, light trap 1|BOLD:ACL2418
Arrenurus|BACWM194-15|Balneario Cocalitos TL3 Pastos|BOLD:ACL2418
Arrenurus|BACWM196-15|Balneario Cocalitos TL3 Pastos|BOLD:ACL2418
Arrenurus|BACWM216-15|Balneario Cocalitos TL3 Pastos|BOLD:ACL2418
Arrenurus|BACZP865-16|Bacalar Lake, Cocalitos Sinkhole|BOLD:ACL2418

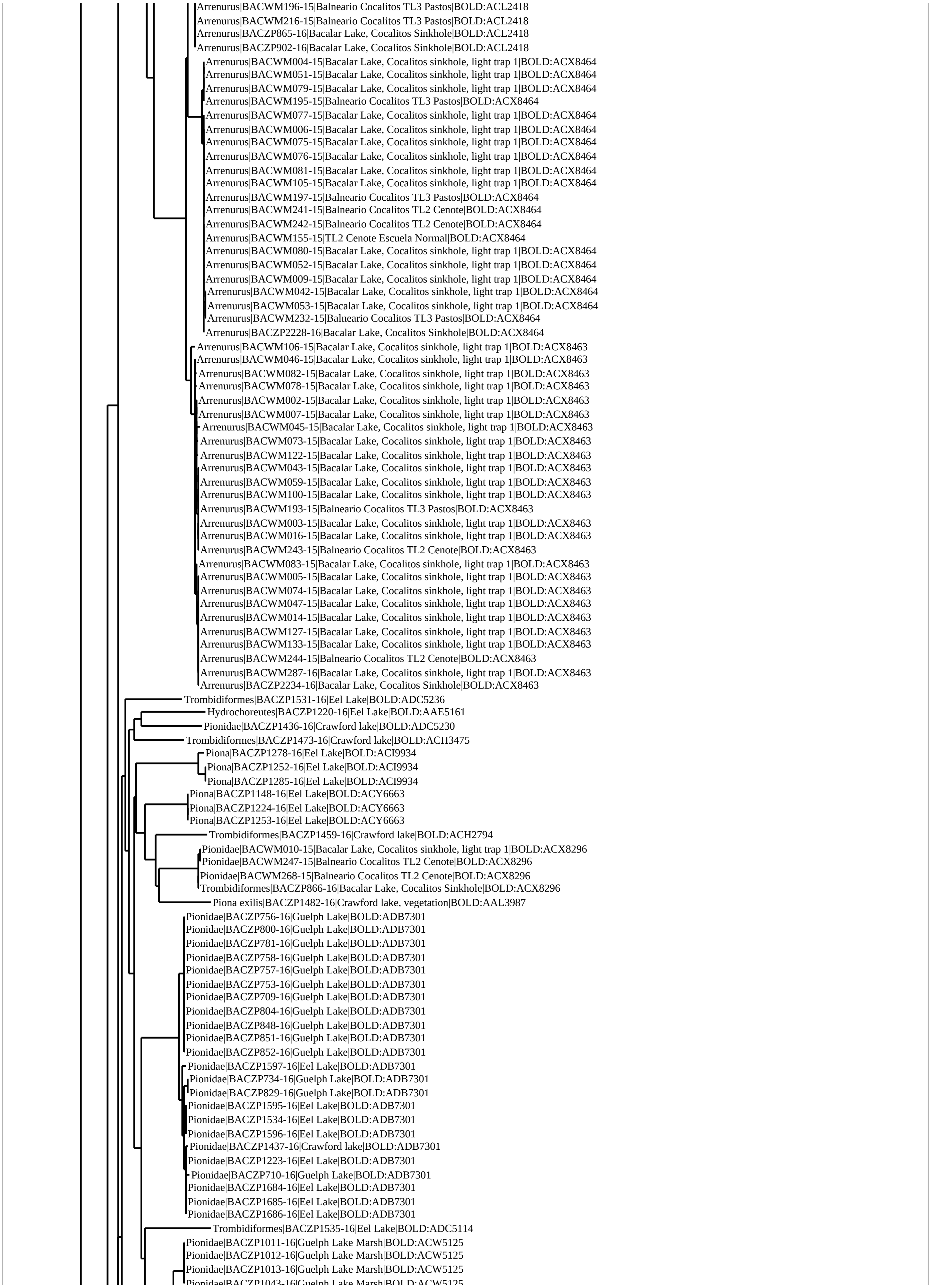
Arrenurus|BACWM196-15|Balneario Cocalitos TL3 Pastos|BOLD:ACL2418
Arrenurus|BACWM216-15|Balneario Cocalitos TL3 Pastos|BOLD:ACL2418
Arrenurus|BACZP865-16|Bacalar Lake, Cocalitos Sinkhole|BOLD:ACL2418
Arrenurus|BACZP902-16|Bacalar Lake, Cocalitos Sinkhole|BOLD:ACL2418
Arrenurus|BACWM004-15|Bacalar Lake, Cocalitos sinkhole, light trap 1|BOLD:ACX8464
Arrenurus|BACWM051-15|Bacalar Lake, Cocalitos sinkhole, light trap 1|BOLD:ACX8464
Arrenurus|BACWM079-15|Bacalar Lake, Cocalitos sinkhole, light trap 1|BOLD:ACX8464
Arrenurus|BACWM195-15|Balneario Cocalitos TL3 Pastos|BOLD:ACX8464
Arrenurus|BACWM077-15|Bacalar Lake, Cocalitos sinkhole, light trap 1|BOLD:ACX8464
Arrenurus|BACWM006-15|Bacalar Lake, Cocalitos sinkhole, light trap 1|BOLD:ACX8464
Arrenurus|BACWM075-15|Bacalar Lake, Cocalitos sinkhole, light trap 1|BOLD:ACX8464
Arrenurus|BACWM076-15|Bacalar Lake, Cocalitos sinkhole, light trap 1|BOLD:ACX8464
Arrenurus|BACWM081-15|Bacalar Lake, Cocalitos sinkhole, light trap 1|BOLD:ACX8464
Arrenurus|BACWM105-15|Bacalar Lake, Cocalitos sinkhole, light trap 1|BOLD:ACX8464
Arrenurus|BACWM197-15|Balneario Cocalitos TL3 Pastos|BOLD:ACX8464
Arrenurus|BACWM241-15|Balneario Cocalitos TL2 Cenote|BOLD:ACX8464
Arrenurus|BACWM242-15|Balneario Cocalitos TL2 Cenote|BOLD:ACX8464
Arrenurus|BACWM155-15|TL2 Cenote Escuela Normal|BOLD:ACX8464
Arrenurus|BACWM080-15|Bacalar Lake, Cocalitos sinkhole, light trap 1|BOLD:ACX8464
Arrenurus|BACWM052-15|Bacalar Lake, Cocalitos sinkhole, light trap 1|BOLD:ACX8464
Arrenurus|BACWM009-15|Bacalar Lake, Cocalitos sinkhole, light trap 1|BOLD:ACX8464
Arrenurus|BACWM042-15|Bacalar Lake, Cocalitos sinkhole, light trap 1|BOLD:ACX8464
Arrenurus|BACWM053-15|Bacalar Lake, Cocalitos sinkhole, light trap 1|BOLD:ACX8464
Arrenurus|BACWM232-15|Balneario Cocalitos TL3 Pastos|BOLD:ACX8464
Arrenurus|BACZP2228-16|Bacalar Lake, Cocalitos Sinkhole|BOLD:ACX8464
Arrenurus|BACWM106-15|Bacalar Lake, Cocalitos sinkhole, light trap 1|BOLD:ACX8463
Arrenurus|BACWM046-15|Bacalar Lake, Cocalitos sinkhole, light trap 1|BOLD:ACX8463
Arrenurus|BACWM082-15|Bacalar Lake, Cocalitos sinkhole, light trap 1|BOLD:ACX8463
Arrenurus|BACWM078-15|Bacalar Lake, Cocalitos sinkhole, light trap 1|BOLD:ACX8463
Arrenurus|BACWM002-15|Bacalar Lake, Cocalitos sinkhole, light trap 1|BOLD:ACX8463
Arrenurus|BACWM007-15|Bacalar Lake, Cocalitos sinkhole, light trap 1|BOLD:ACX8463
Arrenurus|BACWM045-15|Bacalar Lake, Cocalitos sinkhole, light trap 1|BOLD:ACX8463
Arrenurus|BACWM073-15|Bacalar Lake, Cocalitos sinkhole, light trap 1|BOLD:ACX8463
Arrenurus|BACWM122-15|Bacalar Lake, Cocalitos sinkhole, light trap 1|BOLD:ACX8463
Arrenurus|BACWM043-15|Bacalar Lake, Cocalitos sinkhole, light trap 1|BOLD:ACX8463
Arrenurus|BACWM059-15|Bacalar Lake, Cocalitos sinkhole, light trap 1|BOLD:ACX8463
Arrenurus|BACWM100-15|Bacalar Lake, Cocalitos sinkhole, light trap 1|BOLD:ACX8463
Arrenurus|BACWM193-15|Balneario Cocalitos TL3 Pastos|BOLD:ACX8463
Arrenurus|BACWM003-15|Bacalar Lake, Cocalitos sinkhole, light trap 1|BOLD:ACX8463
Arrenurus|BACWM016-15|Bacalar Lake, Cocalitos sinkhole, light trap 1|BOLD:ACX8463
Arrenurus|BACWM243-15|Balneario Cocalitos TL2 Cenote|BOLD:ACX8463
Arrenurus|BACWM083-15|Bacalar Lake, Cocalitos sinkhole, light trap 1|BOLD:ACX8463
Arrenurus|BACWM005-15|Bacalar Lake, Cocalitos sinkhole, light trap 1|BOLD:ACX8463
Arrenurus|BACWM074-15|Bacalar Lake, Cocalitos sinkhole, light trap 1|BOLD:ACX8463
Arrenurus|BACWM047-15|Bacalar Lake, Cocalitos sinkhole, light trap 1|BOLD:ACX8463
Arrenurus|BACWM014-15|Bacalar Lake, Cocalitos sinkhole, light trap 1|BOLD:ACX8463
Arrenurus|BACWM127-15|Bacalar Lake, Cocalitos sinkhole, light trap 1|BOLD:ACX8463
Arrenurus|BACWM133-15|Bacalar Lake, Cocalitos sinkhole, light trap 1|BOLD:ACX8463
Arrenurus|BACWM244-15|Balneario Cocalitos TL2 Cenote|BOLD:ACX8463
Arrenurus|BACWM287-16|Bacalar Lake, Cocalitos sinkhole, light trap 1|BOLD:ACX8463
Arrenurus|BACZP2234-16|Bacalar Lake, Cocalitos Sinkhole|BOLD:ACX8463
Trombidiformes|BACZP1531-16|Eel Lake|BOLD:ADC5236
Hydrochoreutes|BACZP1220-16|Eel Lake|BOLD:AAE5161
Pionidae|BACZP1436-16|Crawford lake|BOLD:ADC5230
Trombidiformes|BACZP1473-16|Crawford lake|BOLD:ACH3475
Piona|BACZP1278-16|Eel Lake|BOLD:ACI9934
Piona|BACZP1252-16|Eel Lake|BOLD:ACI9934
Piona|BACZP1285-16|Eel Lake|BOLD:ACI9934
Piona|BACZP1148-16|Eel Lake|BOLD:ACY6663
Piona|BACZP1224-16|Eel Lake|BOLD:ACY6663
Piona|BACZP1253-16|Eel Lake|BOLD:ACY6663
Trombidiformes|BACZP1459-16|Crawford lake|BOLD:ACH2794
Pionidae|BACWM010-15|Bacalar Lake, Cocalitos sinkhole, light trap 1|BOLD:ACX8296
Pionidae|BACWM247-15|Balneario Cocalitos TL2 Cenote|BOLD:ACX8296
Pionidae|BACWM268-15|Balneario Cocalitos TL2 Cenote|BOLD:ACX8296
Trombidiformes|BACZP866-16|Bacalar Lake, Cocalitos Sinkhole|BOLD:ACX8296
Piona exilis|BACZP1482-16|Crawford lake, vegetation|BOLD:AAL3987
Pionidae|BACZP756-16|Guelph Lake|BOLD:ADB7301
Pionidae|BACZP800-16|Guelph Lake|BOLD:ADB7301
Pionidae|BACZP781-16|Guelph Lake|BOLD:ADB7301
Pionidae|BACZP758-16|Guelph Lake|BOLD:ADB7301
Pionidae|BACZP757-16|Guelph Lake|BOLD:ADB7301
Pionidae|BACZP753-16|Guelph Lake|BOLD:ADB7301
Pionidae|BACZP709-16|Guelph Lake|BOLD:ADB7301
Pionidae|BACZP804-16|Guelph Lake|BOLD:ADB7301
Pionidae|BACZP848-16|Guelph Lake|BOLD:ADB7301
Pionidae|BACZP851-16|Guelph Lake|BOLD:ADB7301
Pionidae|BACZP852-16|Guelph Lake|BOLD:ADB7301
Pionidae|BACZP1597-16|Eel Lake|BOLD:ADB7301
Pionidae|BACZP734-16|Guelph Lake|BOLD:ADB7301
Pionidae|BACZP829-16|Guelph Lake|BOLD:ADB7301
Pionidae|BACZP1595-16|Eel Lake|BOLD:ADB7301
Pionidae|BACZP1534-16|Eel Lake|BOLD:ADB7301
Pionidae|BACZP1596-16|Eel Lake|BOLD:ADB7301
Pionidae|BACZP1437-16|Crawford lake|BOLD:ADB7301
Pionidae|BACZP1223-16|Eel Lake|BOLD:ADB7301
Pionidae|BACZP710-16|Guelph Lake|BOLD:ADB7301
Pionidae|BACZP1684-16|Eel Lake|BOLD:ADB7301
Pionidae|BACZP1685-16|Eel Lake|BOLD:ADB7301
Pionidae|BACZP1686-16|Eel Lake|BOLD:ADB7301
Trombidiformes|BACZP1535-16|Eel Lake|BOLD:ADC5114
Pionidae|BACZP1011-16|Guelph Lake Marsh|BOLD:ACW5125
Pionidae|BACZP1012-16|Guelph Lake Marsh|BOLD:ACW5125
Pionidae|BACZP1013-16|Guelph Lake Marsh|BOLD:ACW5125
Pionidae|BACZP1043-16|Guelph Lake Marsh|BOLD:ACW5125

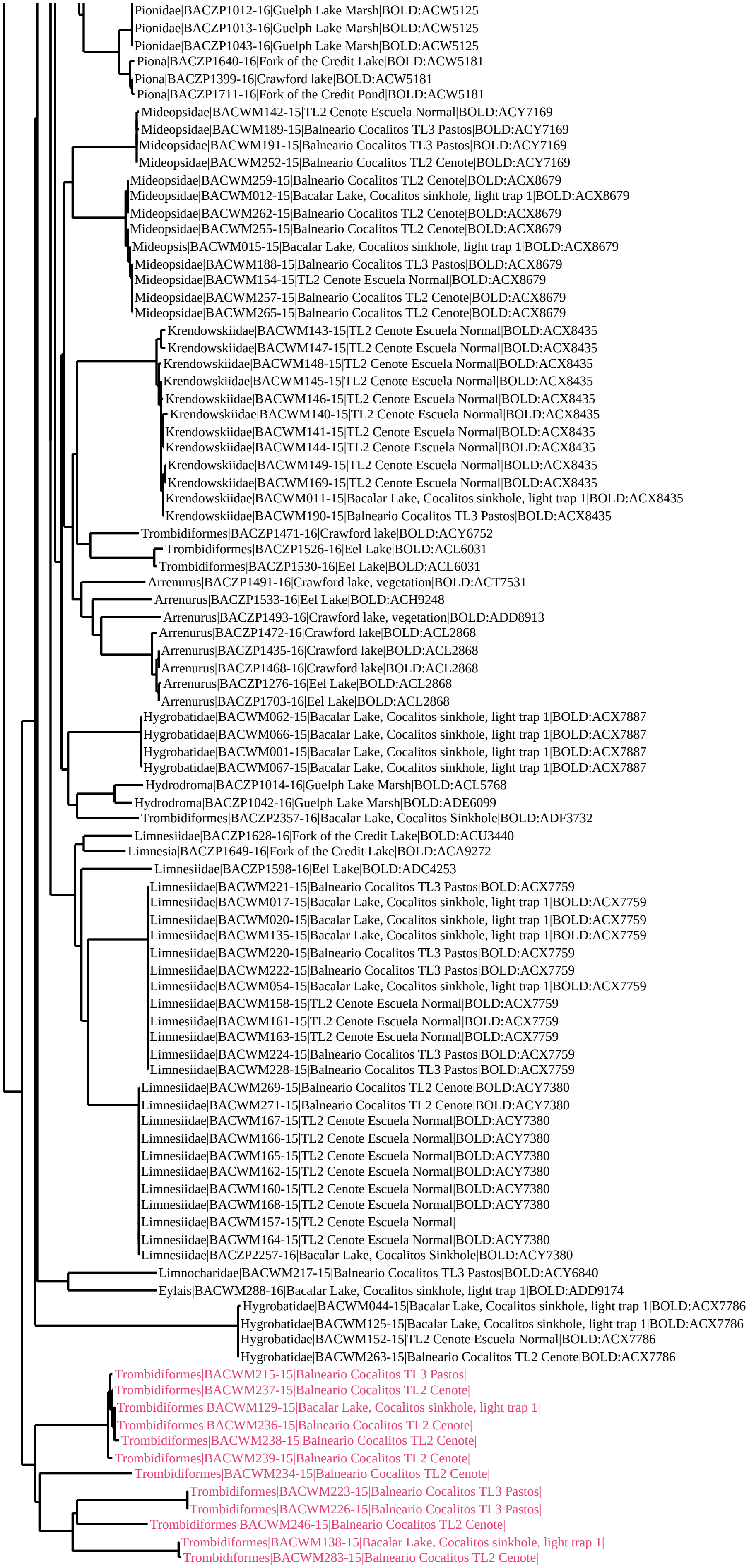
Pionidae|BACZP1012-16|Guelph Lake Marsh|BOLD:ACW5125
Pionidae|BACZP1013-16|Guelph Lake Marsh|BOLD:ACW5125
Pionidae|BACZP1043-16|Guelph Lake Marsh|BOLD:ACW5125
Piona|BACZP1640-16|Fork of the Credit Lake|BOLD:ACW5181
Piona|BACZP1399-16|Crawford lake|BOLD:ACW5181
Piona|BACZP1711-16|Fork of the Credit Pond|BOLD:ACW5181
Mideopsidae|BACWM142-15|TL2 Cenote Escuela Normal|BOLD:ACY7169
Mideopsidae|BACWM189-15|Balneario Cocalitos TL3 Pastos|BOLD:ACY7169
Mideopsidae|BACWM191-15|Balneario Cocalitos TL3 Pastos|BOLD:ACY7169
Mideopsidae|BACWM252-15|Balneario Cocalitos TL2 Cenote|BOLD:ACY7169
Mideopsidae|BACWM259-15|Balneario Cocalitos TL2 Cenote|BOLD:ACX8679
Mideopsidae|BACWM012-15|Bacalar Lake, Cocalitos sinkhole, light trap 1|BOLD:ACX8679
Mideopsidae|BACWM262-15|Balneario Cocalitos TL2 Cenote|BOLD:ACX8679
Mideopsidae|BACWM255-15|Balneario Cocalitos TL2 Cenote|BOLD:ACX8679
Mideopsis|BACWM015-15|Bacalar Lake, Cocalitos sinkhole, light trap 1|BOLD:ACX8679
Mideopsidae|BACWM188-15|Balneario Cocalitos TL3 Pastos|BOLD:ACX8679
Mideopsidae|BACWM154-15|TL2 Cenote Escuela Normal|BOLD:ACX8679
Mideopsidae|BACWM257-15|Balneario Cocalitos TL2 Cenote|BOLD:ACX8679
Mideopsidae|BACWM265-15|Balneario Cocalitos TL2 Cenote|BOLD:ACX8679
Krendowskiidae|BACWM143-15|TL2 Cenote Escuela Normal|BOLD:ACX8435
Krendowskiidae|BACWM147-15|TL2 Cenote Escuela Normal|BOLD:ACX8435
Krendowskiidae|BACWM148-15|TL2 Cenote Escuela Normal|BOLD:ACX8435
Krendowskiidae|BACWM145-15|TL2 Cenote Escuela Normal|BOLD:ACX8435
Krendowskiidae|BACWM146-15|TL2 Cenote Escuela Normal|BOLD:ACX8435
Krendowskiidae|BACWM140-15|TL2 Cenote Escuela Normal|BOLD:ACX8435
Krendowskiidae|BACWM141-15|TL2 Cenote Escuela Normal|BOLD:ACX8435
Krendowskiidae|BACWM144-15|TL2 Cenote Escuela Normal|BOLD:ACX8435
Krendowskiidae|BACWM149-15|TL2 Cenote Escuela Normal|BOLD:ACX8435
Krendowskiidae|BACWM169-15|TL2 Cenote Escuela Normal|BOLD:ACX8435
Krendowskiidae|BACWM011-15|Bacalar Lake, Cocalitos sinkhole, light trap 1|BOLD:ACX8435
Krendowskiidae|BACWM190-15|Balneario Cocalitos TL3 Pastos|BOLD:ACX8435
Trombidiformes|BACZP1471-16|Crawford lake|BOLD:ACY6752
Trombidiformes|BACZP1526-16|Eel Lake|BOLD:ACL6031
Trombidiformes|BACZP1530-16|Eel Lake|BOLD:ACL6031
Arrenurus|BACZP1491-16|Crawford lake, vegetation|BOLD:ACT7531
Arrenurus|BACZP1533-16|Eel Lake|BOLD:ACH9248
Arrenurus|BACZP1493-16|Crawford lake, vegetation|BOLD:ADD8913
Arrenurus|BACZP1472-16|Crawford lake|BOLD:ACL2868
Arrenurus|BACZP1435-16|Crawford lake|BOLD:ACL2868
Arrenurus|BACZP1468-16|Crawford lake|BOLD:ACL2868
Arrenurus|BACZP1276-16|Eel Lake|BOLD:ACL2868
Arrenurus|BACZP1703-16|Eel Lake|BOLD:ACL2868
Hygrobatidae|BACWM062-15|Bacalar Lake, Cocalitos sinkhole, light trap 1|BOLD:ACX7887
Hygrobatidae|BACWM066-15|Bacalar Lake, Cocalitos sinkhole, light trap 1|BOLD:ACX7887
Hygrobatidae|BACWM001-15|Bacalar Lake, Cocalitos sinkhole, light trap 1|BOLD:ACX7887
Hygrobatidae|BACWM067-15|Bacalar Lake, Cocalitos sinkhole, light trap 1|BOLD:ACX7887
Hydrodroma|BACZP1014-16|Guelph Lake Marsh|BOLD:ACL5768
Hydrodroma|BACZP1042-16|Guelph Lake Marsh|BOLD:ADE6099
Trombidiformes|BACZP2357-16|Bacalar Lake, Cocalitos Sinkhole|BOLD:ADF3732
Limnesiidae|BACZP1628-16|Fork of the Credit Lake|BOLD:ACU3440
Limnesia|BACZP1649-16|Fork of the Credit Lake|BOLD:ACA9272
Limnesiidae|BACZP1598-16|Eel Lake|BOLD:ADC4253
Limnesiidae|BACWM221-15|Balneario Cocalitos TL3 Pastos|BOLD:ACX7759
Limnesiidae|BACWM017-15|Bacalar Lake, Cocalitos sinkhole, light trap 1|BOLD:ACX7759
Limnesiidae|BACWM020-15|Bacalar Lake, Cocalitos sinkhole, light trap 1|BOLD:ACX7759
Limnesiidae|BACWM135-15|Bacalar Lake, Cocalitos sinkhole, light trap 1|BOLD:ACX7759
Limnesiidae|BACWM220-15|Balneario Cocalitos TL3 Pastos|BOLD:ACX7759
Limnesiidae|BACWM222-15|Balneario Cocalitos TL3 Pastos|BOLD:ACX7759
Limnesiidae|BACWM054-15|Bacalar Lake, Cocalitos sinkhole, light trap 1|BOLD:ACX7759
Limnesiidae|BACWM158-15|TL2 Cenote Escuela Normal|BOLD:ACX7759
Limnesiidae|BACWM161-15|TL2 Cenote Escuela Normal|BOLD:ACX7759
Limnesiidae|BACWM163-15|TL2 Cenote Escuela Normal|BOLD:ACX7759
Limnesiidae|BACWM224-15|Balneario Cocalitos TL3 Pastos|BOLD:ACX7759
Limnesiidae|BACWM228-15|Balneario Cocalitos TL3 Pastos|BOLD:ACX7759
Limnesiidae|BACWM269-15|Balneario Cocalitos TL2 Cenote|BOLD:ACY7380
Limnesiidae|BACWM271-15|Balneario Cocalitos TL2 Cenote|BOLD:ACY7380
Limnesiidae|BACWM167-15|TL2 Cenote Escuela Normal|BOLD:ACY7380
Limnesiidae|BACWM166-15|TL2 Cenote Escuela Normal|BOLD:ACY7380
Limnesiidae|BACWM165-15|TL2 Cenote Escuela Normal|BOLD:ACY7380
Limnesiidae|BACWM162-15|TL2 Cenote Escuela Normal|BOLD:ACY7380
Limnesiidae|BACWM160-15|TL2 Cenote Escuela Normal|BOLD:ACY7380
Limnesiidae|BACWM168-15|TL2 Cenote Escuela Normal|BOLD:ACY7380
Limnesiidae|BACWM157-15|TL2 Cenote Escuela Normal|
Limnesiidae|BACWM164-15|TL2 Cenote Escuela Normal|BOLD:ACY7380
Limnesiidae|BACZP2257-16|Bacalar Lake, Cocalitos Sinkhole|BOLD:ACY7380
Limnocharidae|BACWM217-15|Balneario Cocalitos TL3 Pastos|BOLD:ACY6840
Eylais|BACWM288-16|Bacalar Lake, Cocalitos sinkhole, light trap 1|BOLD:ADD9174
Hygrobatidae|BACWM044-15|Bacalar Lake, Cocalitos sinkhole, light trap 1|BOLD:ACX7786
Hygrobatidae|BACWM125-15|Bacalar Lake, Cocalitos sinkhole, light trap 1|BOLD:ACX7786
Hygrobatidae|BACWM152-15|TL2 Cenote Escuela Normal|BOLD:ACX7786
Hygrobatidae|BACWM263-15|Balneario Cocalitos TL2 Cenote|BOLD:ACX7786
Trombidiformes|BACWM215-15|Balneario Cocalitos TL3 Pastos|
Trombidiformes|BACWM237-15|Balneario Cocalitos TL2 Cenote|
Trombidiformes|BACWM129-15|Bacalar Lake, Cocalitos sinkhole, light trap 1|
Trombidiformes|BACWM236-15|Balneario Cocalitos TL2 Cenote|
Trombidiformes|BACWM238-15|Balneario Cocalitos TL2 Cenote|
Trombidiformes|BACWM239-15|Balneario Cocalitos TL2 Cenote|
Trombidiformes|BACWM234-15|Balneario Cocalitos TL2 Cenote|
Trombidiformes|BACWM223-15|Balneario Cocalitos TL3 Pastos|
Trombidiformes|BACWM226-15|Balneario Cocalitos TL3 Pastos|
Trombidiformes|BACWM246-15|Balneario Cocalitos TL2 Cenote|
Trombidiformes|BACWM138-15|Bacalar Lake, Cocalitos sinkhole, light trap 1|
Trombidiformes|BACWM283-15|Balneario Cocalitos TL2 Cenote|

BOLD TaxonID Tree

Insecta

```
Title           : SEARCH: Sample ids(213 ids) [SEARCH1]
Date            : 10-March-2017
Data Type       : Nucleotide
Distance Model  : Kimura 2 Parameter
Marker          : COI-5P
Codon Positions : 1st, 2nd, 3rd
Labels          : Site, ProcessID, BIN uri
Filters         : Length > 200
Colorization    : [blue]=Stop Codons [red]=Contamination or misidentification

Sequence Count  : 181
Species count   : 22
Genus count     : 27
Family count    : 16
Unidentified    : 101

BIN Count       : 75
```

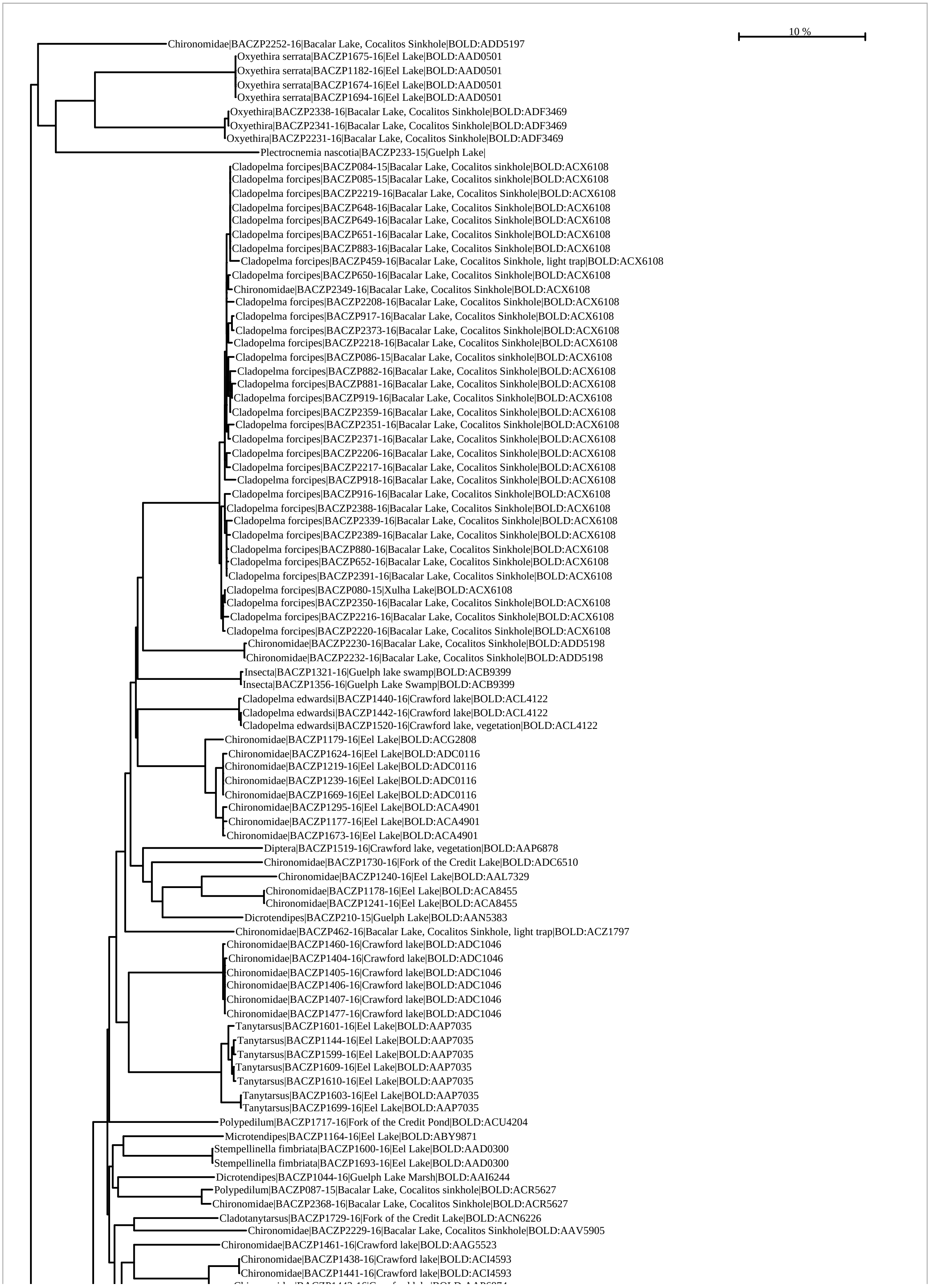
10 %
Chironomidae|BACZP2252-16|Bacalar Lake, Cocalitos Sinkhole|BOLD:ADD5197
Oxyethira serrata|BACZP1675-16|Eel Lake|BOLD:AAD0501
Oxyethira serrata|BACZP1182-16|Eel Lake|BOLD:AAD0501
Oxyethira serrata|BACZP1674-16|Eel Lake|BOLD:AAD0501
Oxyethira serrata|BACZP1694-16|Eel Lake|BOLD:AAD0501
Oxyethira|BACZP2338-16|Bacalar Lake, Cocalitos Sinkhole|BOLD:ADF3469
Oxyethira|BACZP2341-16|Bacalar Lake, Cocalitos Sinkhole|BOLD:ADF3469
Oxyethira|BACZP2231-16|Bacalar Lake, Cocalitos Sinkhole|BOLD:ADF3469
Plectrocnemia nascotia|BACZP233-15|Guelph Lake|
Cladopelma forcipes|BACZP084-15|Bacalar Lake, Cocalitos sinkhole|BOLD:ACX6108
Cladopelma forcipes|BACZP085-15|Bacalar Lake, Cocalitos sinkhole|BOLD:ACX6108
Cladopelma forcipes|BACZP2219-16|Bacalar Lake, Cocalitos Sinkhole|BOLD:ACX6108
Cladopelma forcipes|BACZP648-16|Bacalar Lake, Cocalitos Sinkhole|BOLD:ACX6108
Cladopelma forcipes|BACZP649-16|Bacalar Lake, Cocalitos Sinkhole|BOLD:ACX6108
Cladopelma forcipes|BACZP651-16|Bacalar Lake, Cocalitos Sinkhole|BOLD:ACX6108
Cladopelma forcipes|BACZP883-16|Bacalar Lake, Cocalitos Sinkhole|BOLD:ACX6108
Cladopelma forcipes|BACZP459-16|Bacalar Lake, Cocalitos Sinkhole, light trap|BOLD:ACX6108
Cladopelma forcipes|BACZP650-16|Bacalar Lake, Cocalitos Sinkhole|BOLD:ACX6108
Chironomidae|BACZP2349-16|Bacalar Lake, Cocalitos Sinkhole|BOLD:ACX6108
Cladopelma forcipes|BACZP2208-16|Bacalar Lake, Cocalitos Sinkhole|BOLD:ACX6108
Cladopelma forcipes|BACZP917-16|Bacalar Lake, Cocalitos Sinkhole|BOLD:ACX6108
Cladopelma forcipes|BACZP2373-16|Bacalar Lake, Cocalitos Sinkhole|BOLD:ACX6108
Cladopelma forcipes|BACZP2218-16|Bacalar Lake, Cocalitos Sinkhole|BOLD:ACX6108
Cladopelma forcipes|BACZP086-15|Bacalar Lake, Cocalitos sinkhole|BOLD:ACX6108
Cladopelma forcipes|BACZP882-16|Bacalar Lake, Cocalitos Sinkhole|BOLD:ACX6108
Cladopelma forcipes|BACZP881-16|Bacalar Lake, Cocalitos Sinkhole|BOLD:ACX6108
Cladopelma forcipes|BACZP919-16|Bacalar Lake, Cocalitos Sinkhole|BOLD:ACX6108
Cladopelma forcipes|BACZP2359-16|Bacalar Lake, Cocalitos Sinkhole|BOLD:ACX6108
Cladopelma forcipes|BACZP2351-16|Bacalar Lake, Cocalitos Sinkhole|BOLD:ACX6108
Cladopelma forcipes|BACZP2371-16|Bacalar Lake, Cocalitos Sinkhole|BOLD:ACX6108
Cladopelma forcipes|BACZP2206-16|Bacalar Lake, Cocalitos Sinkhole|BOLD:ACX6108
Cladopelma forcipes|BACZP2217-16|Bacalar Lake, Cocalitos Sinkhole|BOLD:ACX6108
Cladopelma forcipes|BACZP918-16|Bacalar Lake, Cocalitos Sinkhole|BOLD:ACX6108
Cladopelma forcipes|BACZP916-16|Bacalar Lake, Cocalitos Sinkhole|BOLD:ACX6108
Cladopelma forcipes|BACZP2388-16|Bacalar Lake, Cocalitos Sinkhole|BOLD:ACX6108
Cladopelma forcipes|BACZP2339-16|Bacalar Lake, Cocalitos Sinkhole|BOLD:ACX6108
Cladopelma forcipes|BACZP2389-16|Bacalar Lake, Cocalitos Sinkhole|BOLD:ACX6108
Cladopelma forcipes|BACZP880-16|Bacalar Lake, Cocalitos Sinkhole|BOLD:ACX6108
Cladopelma forcipes|BACZP652-16|Bacalar Lake, Cocalitos Sinkhole|BOLD:ACX6108
Cladopelma forcipes|BACZP2391-16|Bacalar Lake, Cocalitos Sinkhole|BOLD:ACX6108
Cladopelma forcipes|BACZP080-15|Xulha Lake|BOLD:ACX6108
Cladopelma forcipes|BACZP2350-16|Bacalar Lake, Cocalitos Sinkhole|BOLD:ACX6108
Cladopelma forcipes|BACZP2216-16|Bacalar Lake, Cocalitos Sinkhole|BOLD:ACX6108
Cladopelma forcipes|BACZP2220-16|Bacalar Lake, Cocalitos Sinkhole|BOLD:ACX6108
Chironomidae|BACZP2230-16|Bacalar Lake, Cocalitos Sinkhole|BOLD:ADD5198
Chironomidae|BACZP2232-16|Bacalar Lake, Cocalitos Sinkhole|BOLD:ADD5198
Insecta|BACZP1321-16|Guelph lake swamp|BOLD:ACB9399
Insecta|BACZP1356-16|Guelph Lake Swamp|BOLD:ACB9399
Cladopelma edwardsi|BACZP1440-16|Crawford lake|BOLD:ACL4122
Cladopelma edwardsi|BACZP1442-16|Crawford lake|BOLD:ACL4122
Cladopelma edwardsi|BACZP1520-16|Crawford lake, vegetation|BOLD:ACL4122
Chironomidae|BACZP1179-16|Eel Lake|BOLD:ACG2808
Chironomidae|BACZP1624-16|Eel Lake|BOLD:ADC0116
Chironomidae|BACZP1219-16|Eel Lake|BOLD:ADC0116
Chironomidae|BACZP1239-16|Eel Lake|BOLD:ADC0116
Chironomidae|BACZP1669-16|Eel Lake|BOLD:ADC0116
Chironomidae|BACZP1295-16|Eel Lake|BOLD:ACA4901
Chironomidae|BACZP1177-16|Eel Lake|BOLD:ACA4901
Chironomidae|BACZP1673-16|Eel Lake|BOLD:ACA4901
Diptera|BACZP1519-16|Crawford lake, vegetation|BOLD:AAP6878
Chironomidae|BACZP1730-16|Fork of the Credit Lake|BOLD:ADC6510
Chironomidae|BACZP1240-16|Eel Lake|BOLD:AAL7329
Chironomidae|BACZP1178-16|Eel Lake|BOLD:ACA8455
Chironomidae|BACZP1241-16|Eel Lake|BOLD:ACA8455
Dicrotendipes|BACZP210-15|Guelph Lake|BOLD:AAN5383
Chironomidae|BACZP462-16|Bacalar Lake, Cocalitos Sinkhole, light trap|BOLD:ACZ1797
Chironomidae|BACZP1460-16|Crawford lake|BOLD:ADC1046
Chironomidae|BACZP1404-16|Crawford lake|BOLD:ADC1046
Chironomidae|BACZP1405-16|Crawford lake|BOLD:ADC1046
Chironomidae|BACZP1406-16|Crawford lake|BOLD:ADC1046
Chironomidae|BACZP1407-16|Crawford lake|BOLD:ADC1046
Chironomidae|BACZP1477-16|Crawford lake|BOLD:ADC1046
Tanytarsus|BACZP1601-16|Eel Lake|BOLD:AAP7035
Tanytarsus|BACZP1144-16|Eel Lake|BOLD:AAP7035
Tanytarsus|BACZP1599-16|Eel Lake|BOLD:AAP7035
Tanytarsus|BACZP1609-16|Eel Lake|BOLD:AAP7035
Tanytarsus|BACZP1610-16|Eel Lake|BOLD:AAP7035
Tanytarsus|BACZP1603-16|Eel Lake|BOLD:AAP7035
Tanytarsus|BACZP1699-16|Eel Lake|BOLD:AAP7035
Polypedilum|BACZP1717-16|Fork of the Credit Pond|BOLD:ACU4204
Microtendipes|BACZP1164-16|Eel Lake|BOLD:ABY9871
Stempellinella fimbriata|BACZP1600-16|Eel Lake|BOLD:AAD0300
Stempellinella fimbriata|BACZP1693-16|Eel Lake|BOLD:AAD0300
Dicrotendipes|BACZP1044-16|Guelph Lake Marsh|BOLD:AAI6244
Polypedilum|BACZP087-15|Bacalar Lake, Cocalitos sinkhole|BOLD:ACR5627
Chironomidae|BACZP2368-16|Bacalar Lake, Cocalitos Sinkhole|BOLD:ACR5627
Cladotanytarsus|BACZP1729-16|Fork of the Credit Lake|BOLD:ACN6226
Chironomidae|BACZP2229-16|Bacalar Lake, Cocalitos Sinkhole|BOLD:AAV5905
Chironomidae|BACZP1461-16|Crawford lake|BOLD:AAG5523
Chironomidae|BACZP1438-16|Crawford lake|BOLD:ACI4593
Chironomidae|BACZP1441-16|Crawford lake|BOLD:ACI4593

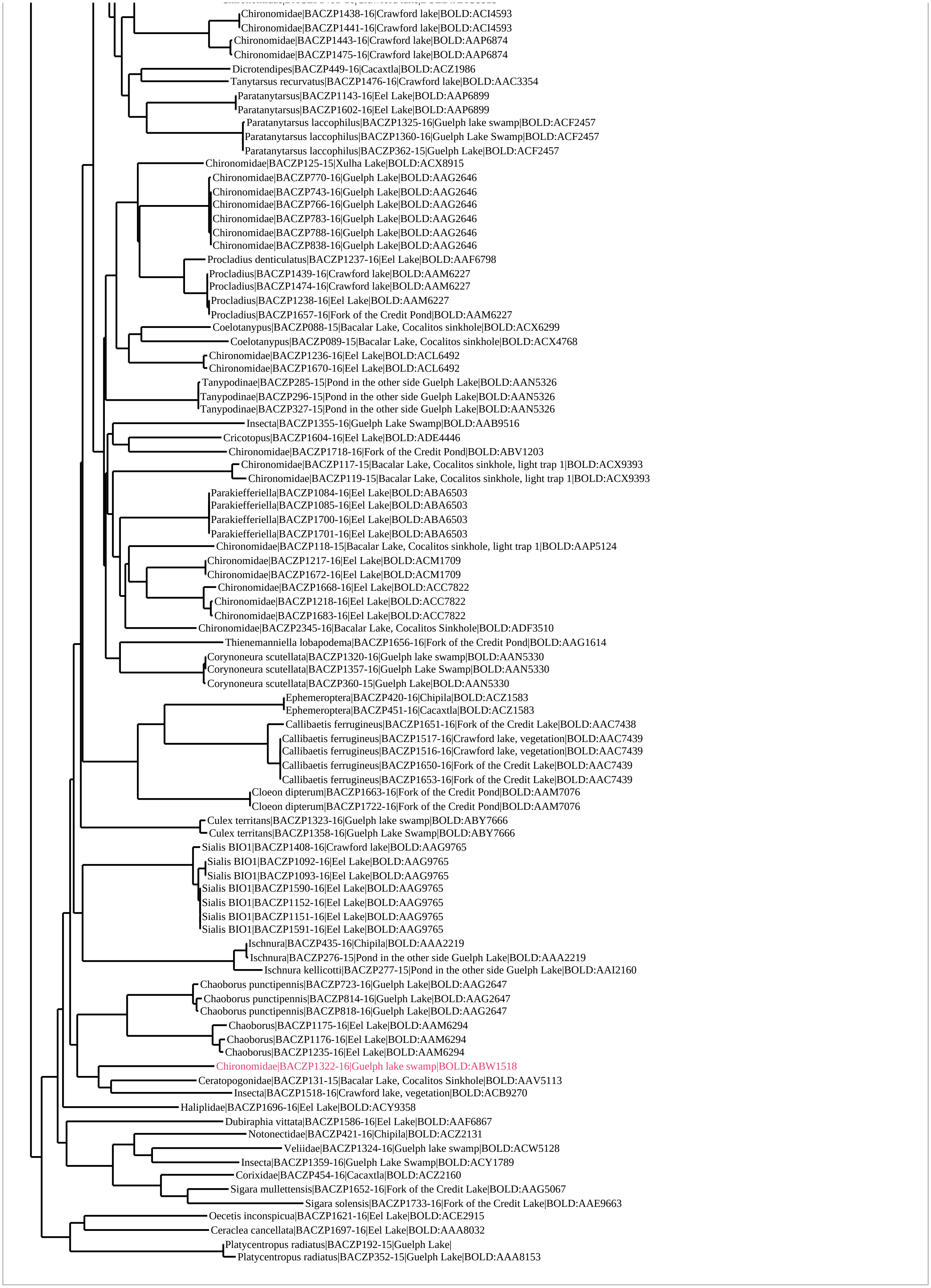
Chironomidae|BACZP1438-16|Crawford lake|BOLD:ACI4593
Chironomidae|BACZP1441-16|Crawford lake|BOLD:ACI4593
Chironomidae|BACZP1443-16|Crawford lake|BOLD:AAP6874
Chironomidae|BACZP1475-16|Crawford lake|BOLD:AAP6874
Dicrotendipes|BACZP449-16|Cacaxtla|BOLD:ACZ1986
Tanytarsus recurvatus|BACZP1476-16|Crawford lake|BOLD:AAC3354
Paratanytarsus|BACZP1143-16|Eel Lake|BOLD:AAP6899
Paratanytarsus|BACZP1602-16|Eel Lake|BOLD:AAP6899
Paratanytarsus laccophilus|BACZP1325-16|Guelph lake swamp|BOLD:ACF2457
Paratanytarsus laccophilus|BACZP1360-16|Guelph Lake Swamp|BOLD:ACF2457
Paratanytarsus laccophilus|BACZP362-15|Guelph Lake|BOLD:ACF2457
Chironomidae|BACZP125-15|Xulha Lake|BOLD:ACX8915
Chironomidae|BACZP770-16|Guelph Lake|BOLD:AAG2646
Chironomidae|BACZP743-16|Guelph Lake|BOLD:AAG2646
Chironomidae|BACZP766-16|Guelph Lake|BOLD:AAG2646
Chironomidae|BACZP783-16|Guelph Lake|BOLD:AAG2646
Chironomidae|BACZP788-16|Guelph Lake|BOLD:AAG2646
Chironomidae|BACZP838-16|Guelph Lake|BOLD:AAG2646
Procladius denticulatus|BACZP1237-16|Eel Lake|BOLD:AAF6798
Procladius|BACZP1439-16|Crawford lake|BOLD:AAM6227
Procladius|BACZP1474-16|Crawford lake|BOLD:AAM6227
Procladius|BACZP1238-16|Eel Lake|BOLD:AAM6227
Procladius|BACZP1657-16|Fork of the Credit Pond|BOLD:AAM6227
Coelotanypus|BACZP088-15|Bacalar Lake, Cocalitos sinkhole|BOLD:ACX6299
Coelotanypus|BACZP089-15|Bacalar Lake, Cocalitos sinkhole|BOLD:ACX4768
Chironomidae|BACZP1236-16|Eel Lake|BOLD:ACL6492
Chironomidae|BACZP1670-16|Eel Lake|BOLD:ACL6492
Tanypodinae|BACZP285-15|Pond in the other side Guelph Lake|BOLD:AAN5326
Tanypodinae|BACZP296-15|Pond in the other side Guelph Lake|BOLD:AAN5326
Tanypodinae|BACZP327-15|Pond in the other side Guelph Lake|BOLD:AAN5326
Insecta|BACZP1355-16|Guelph Lake Swamp|BOLD:AAB9516
Cricotopus|BACZP1604-16|Eel Lake|BOLD:ADE4446
Chironomidae|BACZP1718-16|Fork of the Credit Pond|BOLD:ABV1203
Chironomidae|BACZP117-15|Bacalar Lake, Cocalitos sinkhole, light trap 1|BOLD:ACX9393
Chironomidae|BACZP119-15|Bacalar Lake, Cocalitos sinkhole, light trap 1|BOLD:ACX9393
Parakiefferiella|BACZP1084-16|Eel Lake|BOLD:ABA6503
Parakiefferiella|BACZP1085-16|Eel Lake|BOLD:ABA6503
Parakiefferiella|BACZP1700-16|Eel Lake|BOLD:ABA6503
Parakiefferiella|BACZP1701-16|Eel Lake|BOLD:ABA6503
Chironomidae|BACZP118-15|Bacalar Lake, Cocalitos sinkhole, light trap 1|BOLD:AAP5124
Chironomidae|BACZP1217-16|Eel Lake|BOLD:ACM1709
Chironomidae|BACZP1672-16|Eel Lake|BOLD:ACM1709
Chironomidae|BACZP1668-16|Eel Lake|BOLD:ACC7822
Chironomidae|BACZP1218-16|Eel Lake|BOLD:ACC7822
Chironomidae|BACZP1683-16|Eel Lake|BOLD:ACC7822
Chironomidae|BACZP2345-16|Bacalar Lake, Cocalitos Sinkhole|BOLD:ADF3510
Thienemanniella lobapodema|BACZP1656-16|Fork of the Credit Pond|BOLD:AAG1614
Corynoneura scutellata|BACZP1320-16|Guelph lake swamp|BOLD:AAN5330
Corynoneura scutellata|BACZP1357-16|Guelph Lake Swamp|BOLD:AAN5330
Corynoneura scutellata|BACZP360-15|Guelph Lake|BOLD:AAN5330
Ephemeroptera|BACZP420-16|Chipila|BOLD:ACZ1583
Ephemeroptera|BACZP451-16|Cacaxtla|BOLD:ACZ1583
Callibaetis ferrugineus|BACZP1651-16|Fork of the Credit Lake|BOLD:AAC7438
Callibaetis ferrugineus|BACZP1517-16|Crawford lake, vegetation|BOLD:AAC7439
Callibaetis ferrugineus|BACZP1516-16|Crawford lake, vegetation|BOLD:AAC7439
Callibaetis ferrugineus|BACZP1650-16|Fork of the Credit Lake|BOLD:AAC7439
Callibaetis ferrugineus|BACZP1653-16|Fork of the Credit Lake|BOLD:AAC7439
Cloeon dipterum|BACZP1663-16|Fork of the Credit Pond|BOLD:AAM7076
Cloeon dipterum|BACZP1722-16|Fork of the Credit Pond|BOLD:AAM7076
Culex territans|BACZP1323-16|Guelph lake swamp|BOLD:ABY7666
Culex territans|BACZP1358-16|Guelph Lake Swamp|BOLD:ABY7666
Sialis BIO1|BACZP1408-16|Crawford lake|BOLD:AAG9765
Sialis BIO1|BACZP1092-16|Eel Lake|BOLD:AAG9765
Sialis BIO1|BACZP1093-16|Eel Lake|BOLD:AAG9765
Sialis BIO1|BACZP1590-16|Eel Lake|BOLD:AAG9765
Sialis BIO1|BACZP1152-16|Eel Lake|BOLD:AAG9765
Sialis BIO1|BACZP1151-16|Eel Lake|BOLD:AAG9765
Sialis BIO1|BACZP1591-16|Eel Lake|BOLD:AAG9765
Ischnura|BACZP435-16|Chipila|BOLD:AAA2219
Ischnura|BACZP276-15|Pond in the other side Guelph Lake|BOLD:AAA2219
Ischnura kellicotti|BACZP277-15|Pond in the other side Guelph Lake|BOLD:AAI2160
Chaoborus punctipennis|BACZP723-16|Guelph Lake|BOLD:AAG2647
Chaoborus punctipennis|BACZP814-16|Guelph Lake|BOLD:AAG2647
Chaoborus punctipennis|BACZP818-16|Guelph Lake|BOLD:AAG2647
Chaoborus|BACZP1175-16|Eel Lake|BOLD:AAM6294
Chaoborus|BACZP1176-16|Eel Lake|BOLD:AAM6294
Chaoborus|BACZP1235-16|Eel Lake|BOLD:AAM6294
Chironomidae|BACZP1322-16|Guelph lake swamp|BOLD:ABW1518
Ceratopogonidae|BACZP131-15|Bacalar Lake, Cocalitos Sinkhole|BOLD:AAV5113
Insecta|BACZP1518-16|Crawford lake, vegetation|BOLD:ACB9270
Haliplidae|BACZP1696-16|Eel Lake|BOLD:ACY9358
Dubiraphia vittata|BACZP1586-16|Eel Lake|BOLD:AAF6867
Notonectidae|BACZP421-16|Chipila|BOLD:ACZ2131
Veliidae|BACZP1324-16|Guelph lake swamp|BOLD:ACW5128
Insecta|BACZP1359-16|Guelph Lake Swamp|BOLD:ACY1789
Corixidae|BACZP454-16|Cacaxtla|BOLD:ACZ2160
Sigara mullettensis|BACZP1652-16|Fork of the Credit Lake|BOLD:AAG5067
Sigara solensis|BACZP1733-16|Fork of the Credit Lake|BOLD:AAE9663
Oecetis inconspicua|BACZP1621-16|Eel Lake|BOLD:ACE2915
Ceraclea cancellata|BACZP1697-16|Eel Lake|BOLD:AAA8032
Platycentropus radiatus|BACZP192-15|Guelph Lake|
Platycentropus radiatus|BACZP352-15|Guelph Lake|BOLD:AAA8153

BOLD TaxonID Tree

Crustacea

Title	:	SEARCH: Sample ids(1278 ids) [SEARCH2]
Date	:	10-March-2017
Data Type	:	Nucleotide
Distance Model	:	Kimura 2 Parameter
Marker	:	COI-5P
Codon Positions	:	1st, 2nd, 3rd
Labels	:	Site, ProcessID, BIN uri
Filters	:	Length > 200
Colorization	:	[blue]=Stop Codons [red]=Contamination or misidentification

Sequence Count	:	1080
Species count	:	46
Genus count	:	41
Family count	:	22
Unidentified	:	602

BIN Count	:	161

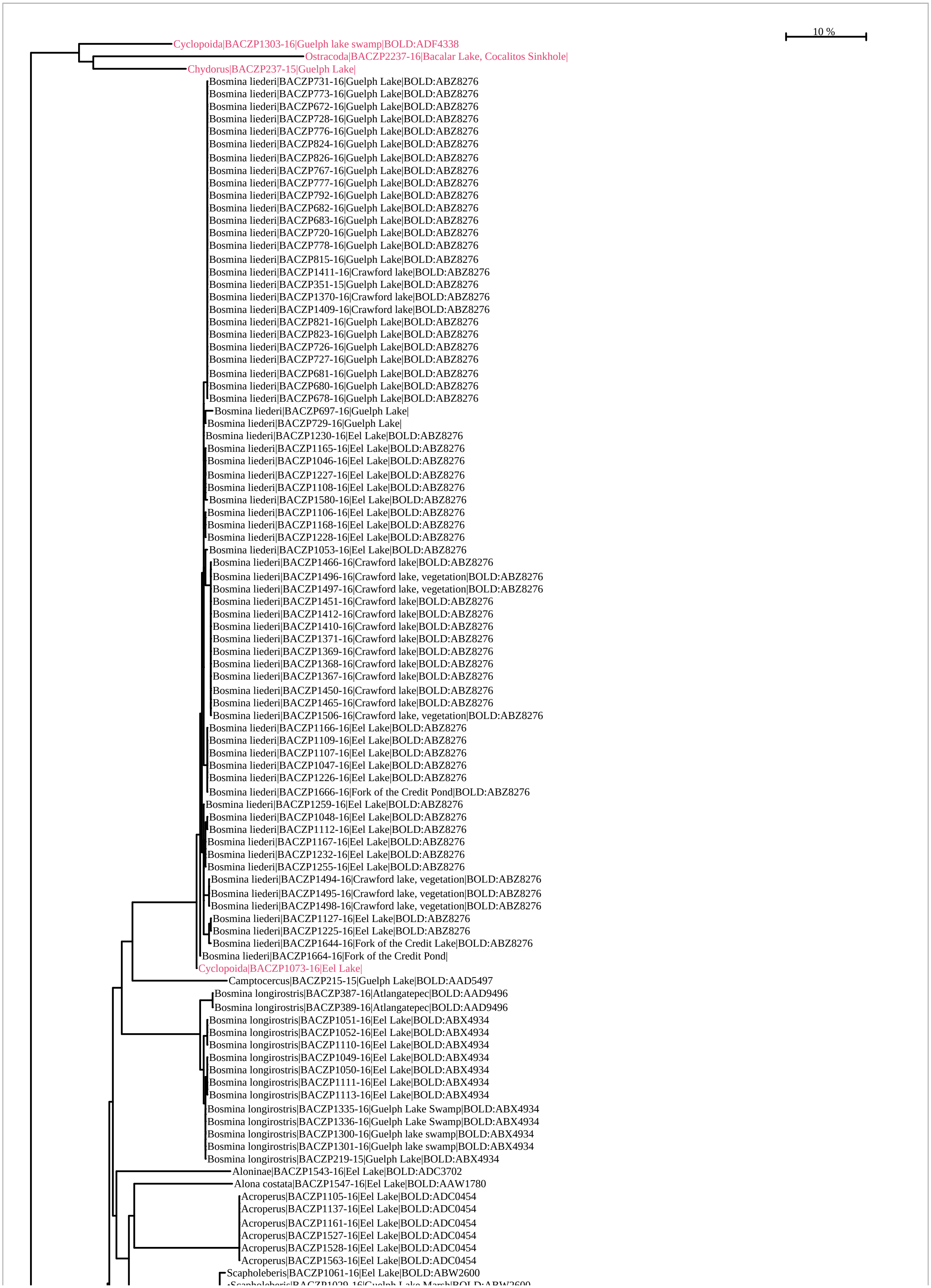
10 %
Cyclopoida|BACZP1303-16|Guelph lake swamp|BOLD:ADF4338
Ostracoda|BACZP2237-16|Bacalar Lake, Cocalitos Sinkhole|
Chydorus|BACZP237-15|Guelph Lake|
Bosmina liederi|BACZP731-16|Guelph Lake|BOLD:ABZ8276
Bosmina liederi|BACZP773-16|Guelph Lake|BOLD:ABZ8276
Bosmina liederi|BACZP672-16|Guelph Lake|BOLD:ABZ8276
Bosmina liederi|BACZP728-16|Guelph Lake|BOLD:ABZ8276
Bosmina liederi|BACZP776-16|Guelph Lake|BOLD:ABZ8276
Bosmina liederi|BACZP824-16|Guelph Lake|BOLD:ABZ8276
Bosmina liederi|BACZP826-16|Guelph Lake|BOLD:ABZ8276
Bosmina liederi|BACZP767-16|Guelph Lake|BOLD:ABZ8276
Bosmina liederi|BACZP777-16|Guelph Lake|BOLD:ABZ8276
Bosmina liederi|BACZP792-16|Guelph Lake|BOLD:ABZ8276
Bosmina liederi|BACZP682-16|Guelph Lake|BOLD:ABZ8276
Bosmina liederi|BACZP683-16|Guelph Lake|BOLD:ABZ8276
Bosmina liederi|BACZP720-16|Guelph Lake|BOLD:ABZ8276
Bosmina liederi|BACZP778-16|Guelph Lake|BOLD:ABZ8276
Bosmina liederi|BACZP815-16|Guelph Lake|BOLD:ABZ8276
Bosmina liederi|BACZP1411-16|Crawford lake|BOLD:ABZ8276
Bosmina liederi|BACZP351-15|Guelph Lake|BOLD:ABZ8276
Bosmina liederi|BACZP1370-16|Crawford lake|BOLD:ABZ8276
Bosmina liederi|BACZP1409-16|Crawford lake|BOLD:ABZ8276
Bosmina liederi|BACZP821-16|Guelph Lake|BOLD:ABZ8276
Bosmina liederi|BACZP823-16|Guelph Lake|BOLD:ABZ8276
Bosmina liederi|BACZP726-16|Guelph Lake|BOLD:ABZ8276
Bosmina liederi|BACZP727-16|Guelph Lake|BOLD:ABZ8276
Bosmina liederi|BACZP681-16|Guelph Lake|BOLD:ABZ8276
Bosmina liederi|BACZP680-16|Guelph Lake|BOLD:ABZ8276
Bosmina liederi|BACZP678-16|Guelph Lake|BOLD:ABZ8276
Bosmina liederi|BACZP697-16|Guelph Lake|
Bosmina liederi|BACZP729-16|Guelph Lake|
Bosmina liederi|BACZP1230-16|Eel Lake|BOLD:ABZ8276
Bosmina liederi|BACZP1165-16|Eel Lake|BOLD:ABZ8276
Bosmina liederi|BACZP1046-16|Eel Lake|BOLD:ABZ8276
Bosmina liederi|BACZP1227-16|Eel Lake|BOLD:ABZ8276
Bosmina liederi|BACZP1108-16|Eel Lake|BOLD:ABZ8276
Bosmina liederi|BACZP1580-16|Eel Lake|BOLD:ABZ8276
Bosmina liederi|BACZP1106-16|Eel Lake|BOLD:ABZ8276
Bosmina liederi|BACZP1168-16|Eel Lake|BOLD:ABZ8276
Bosmina liederi|BACZP1228-16|Eel Lake|BOLD:ABZ8276
Bosmina liederi|BACZP1053-16|Eel Lake|BOLD:ABZ8276
Bosmina liederi|BACZP1466-16|Crawford lake|BOLD:ABZ8276
Bosmina liederi|BACZP1496-16|Crawford lake, vegetation|BOLD:ABZ8276
Bosmina liederi|BACZP1497-16|Crawford lake, vegetation|BOLD:ABZ8276
Bosmina liederi|BACZP1451-16|Crawford lake|BOLD:ABZ8276
Bosmina liederi|BACZP1412-16|Crawford lake|BOLD:ABZ8276
Bosmina liederi|BACZP1410-16|Crawford lake|BOLD:ABZ8276
Bosmina liederi|BACZP1371-16|Crawford lake|BOLD:ABZ8276
Bosmina liederi|BACZP1369-16|Crawford lake|BOLD:ABZ8276
Bosmina liederi|BACZP1368-16|Crawford lake|BOLD:ABZ8276
Bosmina liederi|BACZP1367-16|Crawford lake|BOLD:ABZ8276
Bosmina liederi|BACZP1450-16|Crawford lake|BOLD:ABZ8276
Bosmina liederi|BACZP1465-16|Crawford lake|BOLD:ABZ8276
Bosmina liederi|BACZP1506-16|Crawford lake, vegetation|BOLD:ABZ8276
Bosmina liederi|BACZP1166-16|Eel Lake|BOLD:ABZ8276
Bosmina liederi|BACZP1109-16|Eel Lake|BOLD:ABZ8276
Bosmina liederi|BACZP1107-16|Eel Lake|BOLD:ABZ8276
Bosmina liederi|BACZP1047-16|Eel Lake|BOLD:ABZ8276
Bosmina liederi|BACZP1226-16|Eel Lake|BOLD:ABZ8276
Bosmina liederi|BACZP1666-16|Fork of the Credit Pond|BOLD:ABZ8276
Bosmina liederi|BACZP1259-16|Eel Lake|BOLD:ABZ8276
Bosmina liederi|BACZP1048-16|Eel Lake|BOLD:ABZ8276
Bosmina liederi|BACZP1112-16|Eel Lake|BOLD:ABZ8276
Bosmina liederi|BACZP1167-16|Eel Lake|BOLD:ABZ8276
Bosmina liederi|BACZP1232-16|Eel Lake|BOLD:ABZ8276
Bosmina liederi|BACZP1255-16|Eel Lake|BOLD:ABZ8276
Bosmina liederi|BACZP1494-16|Crawford lake, vegetation|BOLD:ABZ8276
Bosmina liederi|BACZP1495-16|Crawford lake, vegetation|BOLD:ABZ8276
Bosmina liederi|BACZP1498-16|Crawford lake, vegetation|BOLD:ABZ8276
Bosmina liederi|BACZP1127-16|Eel Lake|BOLD:ABZ8276
Bosmina liederi|BACZP1225-16|Eel Lake|BOLD:ABZ8276
Bosmina liederi|BACZP1644-16|Fork of the Credit Lake|BOLD:ABZ8276
Bosmina liederi|BACZP1664-16|Fork of the Credit Pond|
Cyclopoida|BACZP1073-16|Eel Lake|
Camptocercus|BACZP215-15|Guelph Lake|BOLD:AAD5497
Bosmina longirostris|BACZP387-16|Atlangatepec|BOLD:AAD9496
Bosmina longirostris|BACZP389-16|Atlangatepec|BOLD:AAD9496
Bosmina longirostris|BACZP1051-16|Eel Lake|BOLD:ABX4934
Bosmina longirostris|BACZP1052-16|Eel Lake|BOLD:ABX4934
Bosmina longirostris|BACZP1110-16|Eel Lake|BOLD:ABX4934
Bosmina longirostris|BACZP1049-16|Eel Lake|BOLD:ABX4934
Bosmina longirostris|BACZP1050-16|Eel Lake|BOLD:ABX4934
Bosmina longirostris|BACZP1111-16|Eel Lake|BOLD:ABX4934
Bosmina longirostris|BACZP1113-16|Eel Lake|BOLD:ABX4934
Bosmina longirostris|BACZP1335-16|Guelph Lake Swamp|BOLD:ABX4934
Bosmina longirostris|BACZP1336-16|Guelph Lake Swamp|BOLD:ABX4934
Bosmina longirostris|BACZP1300-16|Guelph lake swamp|BOLD:ABX4934
Bosmina longirostris|BACZP1301-16|Guelph lake swamp|BOLD:ABX4934
Bosmina longirostris|BACZP219-15|Guelph Lake|BOLD:ABX4934
Aloninae|BACZP1543-16|Eel Lake|BOLD:ADC3702
Alona costata|BACZP1547-16|Eel Lake|BOLD:AAW1780
Acroperus|BACZP1105-16|Eel Lake|BOLD:ADC0454
Acroperus|BACZP1137-16|Eel Lake|BOLD:ADC0454
Acroperus|BACZP1161-16|Eel Lake|BOLD:ADC0454
Acroperus|BACZP1527-16|Eel Lake|BOLD:ADC0454
Acroperus|BACZP1528-16|Eel Lake|BOLD:ADC0454
Acroperus|BACZP1563-16|Eel Lake|BOLD:ADC0454
Scapholeberis|BACZP1061-16|Eel Lake|BOLD:ABW2600

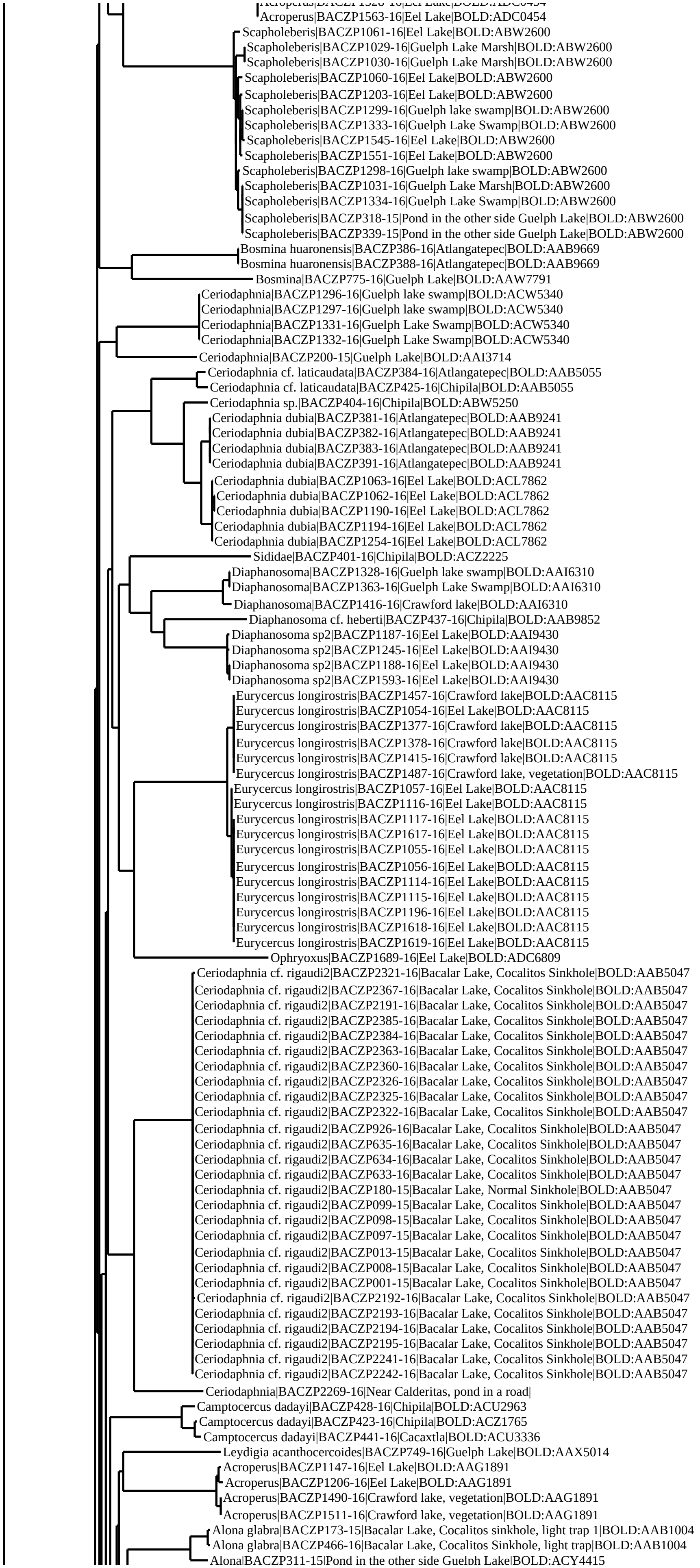
Acroperus|BACZP1563-16|Eel Lake|BOLD:ADC0454
Scapholeberis|BACZP1061-16|Eel Lake|BOLD:ABW2600
Scapholeberis|BACZP1029-16|Guelph Lake Marsh|BOLD:ABW2600
Scapholeberis|BACZP1030-16|Guelph Lake Marsh|BOLD:ABW2600
Scapholeberis|BACZP1060-16|Eel Lake|BOLD:ABW2600
Scapholeberis|BACZP1203-16|Eel Lake|BOLD:ABW2600
Scapholeberis|BACZP1299-16|Guelph lake swamp|BOLD:ABW2600
Scapholeberis|BACZP1333-16|Guelph Lake Swamp|BOLD:ABW2600
Scapholeberis|BACZP1545-16|Eel Lake|BOLD:ABW2600
Scapholeberis|BACZP1551-16|Eel Lake|BOLD:ABW2600
Scapholeberis|BACZP1298-16|Guelph lake swamp|BOLD:ABW2600
Scapholeberis|BACZP1031-16|Guelph Lake Marsh|BOLD:ABW2600
Scapholeberis|BACZP1334-16|Guelph Lake Swamp|BOLD:ABW2600
Scapholeberis|BACZP318-15|Pond in the other side Guelph Lake|BOLD:ABW2600
Scapholeberis|BACZP339-15|Pond in the other side Guelph Lake|BOLD:ABW2600
Bosmina huaronensis|BACZP386-16|Atlangatepec|BOLD:AAB9669
Bosmina huaronensis|BACZP388-16|Atlangatepec|BOLD:AAB9669
Bosmina|BACZP775-16|Guelph Lake|BOLD:AAW7791
Ceriodaphnia|BACZP1296-16|Guelph lake swamp|BOLD:ACW5340
Ceriodaphnia|BACZP1297-16|Guelph lake swamp|BOLD:ACW5340
Ceriodaphnia|BACZP1331-16|Guelph Lake Swamp|BOLD:ACW5340
Ceriodaphnia|BACZP1332-16|Guelph Lake Swamp|BOLD:ACW5340
Ceriodaphnia|BACZP200-15|Guelph Lake|BOLD:AAI3714
Ceriodaphnia cf. laticaudata|BACZP384-16|Atlangatepec|BOLD:AAB5055
Ceriodaphnia cf. laticaudata|BACZP425-16|Chipila|BOLD:AAB5055
Ceriodaphnia sp.|BACZP404-16|Chipila|BOLD:ABW5250
Ceriodaphnia dubia|BACZP381-16|Atlangatepec|BOLD:AAB9241
Ceriodaphnia dubia|BACZP382-16|Atlangatepec|BOLD:AAB9241
Ceriodaphnia dubia|BACZP383-16|Atlangatepec|BOLD:AAB9241
Ceriodaphnia dubia|BACZP391-16|Atlangatepec|BOLD:AAB9241
Ceriodaphnia dubia|BACZP1063-16|Eel Lake|BOLD:ACL7862
Ceriodaphnia dubia|BACZP1062-16|Eel Lake|BOLD:ACL7862
Ceriodaphnia dubia|BACZP1190-16|Eel Lake|BOLD:ACL7862
Ceriodaphnia dubia|BACZP1194-16|Eel Lake|BOLD:ACL7862
Ceriodaphnia dubia|BACZP1254-16|Eel Lake|BOLD:ACL7862
Sididae|BACZP401-16|Chipila|BOLD:ACZ2225
Diaphanosoma|BACZP1328-16|Guelph lake swamp|BOLD:AAI6310
Diaphanosoma|BACZP1363-16|Guelph Lake Swamp|BOLD:AAI6310
Diaphanosoma|BACZP1416-16|Crawford lake|BOLD:AAI6310
Diaphanosoma cf. heberti|BACZP437-16|Chipila|BOLD:AAB9852
Diaphanosoma sp2|BACZP1187-16|Eel Lake|BOLD:AAI9430
Diaphanosoma sp2|BACZP1245-16|Eel Lake|BOLD:AAI9430
Diaphanosoma sp2|BACZP1188-16|Eel Lake|BOLD:AAI9430
Diaphanosoma sp2|BACZP1593-16|Eel Lake|BOLD:AAI9430
Eurycercus longirostris|BACZP1457-16|Crawford lake|BOLD:AAC8115
Eurycercus longirostris|BACZP1054-16|Eel Lake|BOLD:AAC8115
Eurycercus longirostris|BACZP1377-16|Crawford lake|BOLD:AAC8115
Eurycercus longirostris|BACZP1378-16|Crawford lake|BOLD:AAC8115
Eurycercus longirostris|BACZP1415-16|Crawford lake|BOLD:AAC8115
Eurycercus longirostris|BACZP1487-16|Crawford lake, vegetation|BOLD:AAC8115
Eurycercus longirostris|BACZP1057-16|Eel Lake|BOLD:AAC8115
Eurycercus longirostris|BACZP1116-16|Eel Lake|BOLD:AAC8115
Eurycercus longirostris|BACZP1117-16|Eel Lake|BOLD:AAC8115
Eurycercus longirostris|BACZP1617-16|Eel Lake|BOLD:AAC8115
Eurycercus longirostris|BACZP1055-16|Eel Lake|BOLD:AAC8115
Eurycercus longirostris|BACZP1056-16|Eel Lake|BOLD:AAC8115
Eurycercus longirostris|BACZP1114-16|Eel Lake|BOLD:AAC8115
Eurycercus longirostris|BACZP1115-16|Eel Lake|BOLD:AAC8115
Eurycercus longirostris|BACZP1196-16|Eel Lake|BOLD:AAC8115
Eurycercus longirostris|BACZP1618-16|Eel Lake|BOLD:AAC8115
Eurycercus longirostris|BACZP1619-16|Eel Lake|BOLD:AAC8115
Ophryoxus|BACZP1689-16|Eel Lake|BOLD:ADC6809
Ceriodaphnia cf. rigaudi2|BACZP2321-16|Bacalar Lake, Cocalitos Sinkhole|BOLD:AAB5047
Ceriodaphnia cf. rigaudi2|BACZP2367-16|Bacalar Lake, Cocalitos Sinkhole|BOLD:AAB5047
Ceriodaphnia cf. rigaudi2|BACZP2191-16|Bacalar Lake, Cocalitos Sinkhole|BOLD:AAB5047
Ceriodaphnia cf. rigaudi2|BACZP2385-16|Bacalar Lake, Cocalitos Sinkhole|BOLD:AAB5047
Ceriodaphnia cf. rigaudi2|BACZP2384-16|Bacalar Lake, Cocalitos Sinkhole|BOLD:AAB5047
Ceriodaphnia cf. rigaudi2|BACZP2363-16|Bacalar Lake, Cocalitos Sinkhole|BOLD:AAB5047
Ceriodaphnia cf. rigaudi2|BACZP2360-16|Bacalar Lake, Cocalitos Sinkhole|BOLD:AAB5047
Ceriodaphnia cf. rigaudi2|BACZP2326-16|Bacalar Lake, Cocalitos Sinkhole|BOLD:AAB5047
Ceriodaphnia cf. rigaudi2|BACZP2325-16|Bacalar Lake, Cocalitos Sinkhole|BOLD:AAB5047
Ceriodaphnia cf. rigaudi2|BACZP2322-16|Bacalar Lake, Cocalitos Sinkhole|BOLD:AAB5047
Ceriodaphnia cf. rigaudi2|BACZP926-16|Bacalar Lake, Cocalitos Sinkhole|BOLD:AAB5047
Ceriodaphnia cf. rigaudi2|BACZP635-16|Bacalar Lake, Cocalitos Sinkhole|BOLD:AAB5047
Ceriodaphnia cf. rigaudi2|BACZP634-16|Bacalar Lake, Cocalitos Sinkhole|BOLD:AAB5047
Ceriodaphnia cf. rigaudi2|BACZP633-16|Bacalar Lake, Cocalitos Sinkhole|BOLD:AAB5047
Ceriodaphnia cf. rigaudi2|BACZP180-15|Bacalar Lake, Normal Sinkhole|BOLD:AAB5047
Ceriodaphnia cf. rigaudi2|BACZP099-15|Bacalar Lake, Cocalitos Sinkhole|BOLD:AAB5047
Ceriodaphnia cf. rigaudi2|BACZP098-15|Bacalar Lake, Cocalitos Sinkhole|BOLD:AAB5047
Ceriodaphnia cf. rigaudi2|BACZP097-15|Bacalar Lake, Cocalitos Sinkhole|BOLD:AAB5047
Ceriodaphnia cf. rigaudi2|BACZP013-15|Bacalar Lake, Cocalitos Sinkhole|BOLD:AAB5047
Ceriodaphnia cf. rigaudi2|BACZP008-15|Bacalar Lake, Cocalitos Sinkhole|BOLD:AAB5047
Ceriodaphnia cf. rigaudi2|BACZP001-15|Bacalar Lake, Cocalitos Sinkhole|BOLD:AAB5047
Ceriodaphnia cf. rigaudi2|BACZP2192-16|Bacalar Lake, Cocalitos Sinkhole|BOLD:AAB5047
Ceriodaphnia cf. rigaudi2|BACZP2193-16|Bacalar Lake, Cocalitos Sinkhole|BOLD:AAB5047
Ceriodaphnia cf. rigaudi2|BACZP2194-16|Bacalar Lake, Cocalitos Sinkhole|BOLD:AAB5047
Ceriodaphnia cf. rigaudi2|BACZP2195-16|Bacalar Lake, Cocalitos Sinkhole|BOLD:AAB5047
Ceriodaphnia cf. rigaudi2|BACZP2241-16|Bacalar Lake, Cocalitos Sinkhole|BOLD:AAB5047
Ceriodaphnia cf. rigaudi2|BACZP2242-16|Bacalar Lake, Cocalitos Sinkhole|BOLD:AAB5047
Ceriodaphnia|BACZP2269-16|Near Calderitas, pond in a road|
Camptocercus dadayi|BACZP428-16|Chipila|BOLD:ACU2963
Camptocercus dadayi|BACZP423-16|Chipila|BOLD:ACZ1765
Camptocercus dadayi|BACZP441-16|Cacaxtla|BOLD:ACU3336
Leydigia acanthocercoides|BACZP749-16|Guelph Lake|BOLD:AAX5014
Acroperus|BACZP1147-16|Eel Lake|BOLD:AAG1891
Acroperus|BACZP1206-16|Eel Lake|BOLD:AAG1891
Acroperus|BACZP1490-16|Crawford lake, vegetation|BOLD:AAG1891
Acroperus|BACZP1511-16|Crawford lake, vegetation|BOLD:AAG1891
Alona glabra|BACZP173-15|Bacalar Lake, Cocalitos sinkhole, light trap 1|BOLD:AAB1004
Alona glabra|BACZP466-16|Bacalar Lake, Cocalitos Sinkhole, light trap|BOLD:AAB1004
Alona|BACZP311-15|Pond in the other side Guelph Lake|BOLD:ACY4415

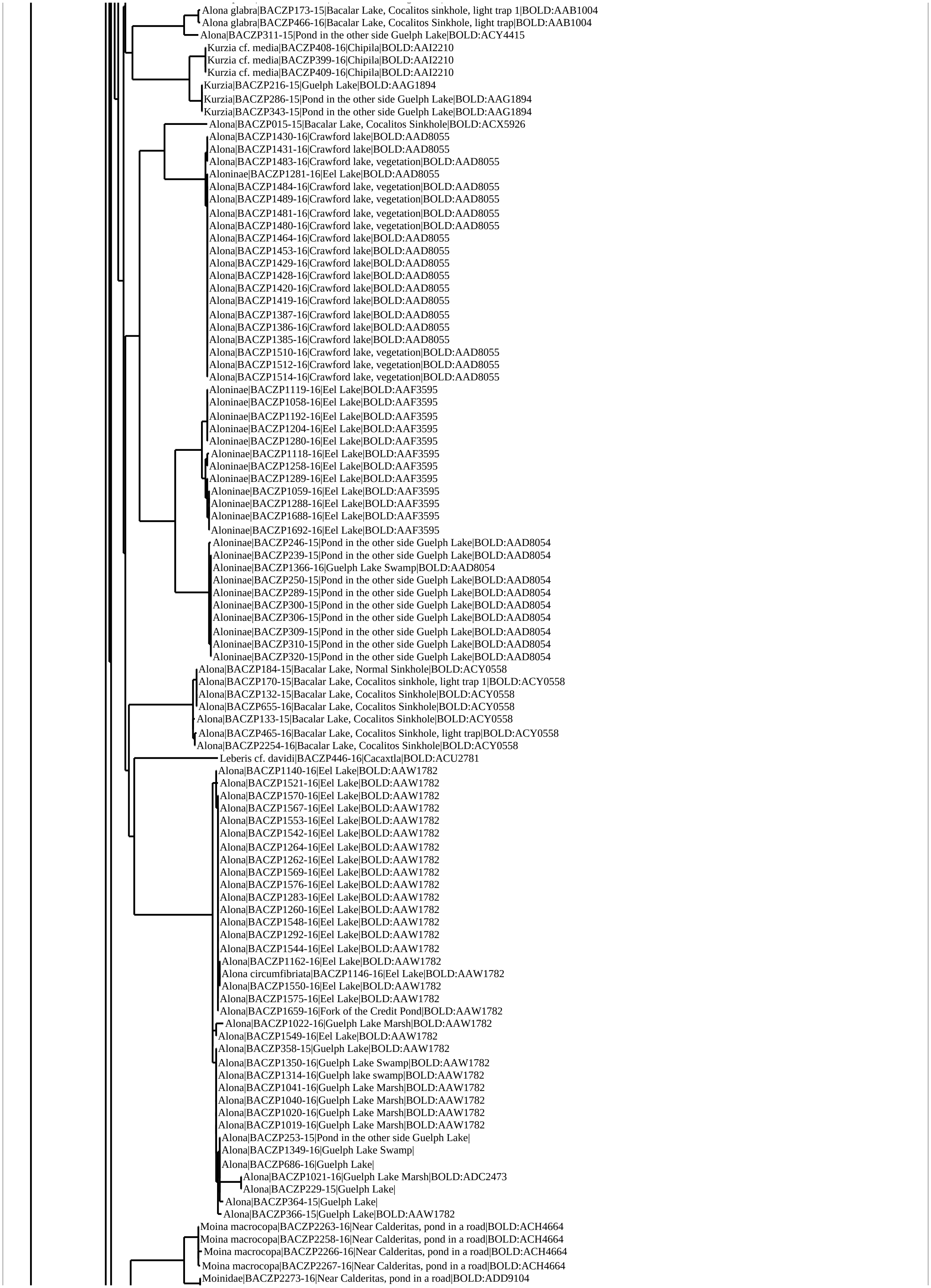
Alona glabra|BACZP173-15|Bacalar Lake, Cocalitos sinkhole, light trap 1|BOLD:AAB1004
Alona glabra|BACZP466-16|Bacalar Lake, Cocalitos Sinkhole, light trap|BOLD:AAB1004
Alona|BACZP311-15|Pond in the other side Guelph Lake|BOLD:ACY4415
Kurzia cf. media|BACZP408-16|Chipila|BOLD:AAI2210
Kurzia cf. media|BACZP399-16|Chipila|BOLD:AAI2210
Kurzia cf. media|BACZP409-16|Chipila|BOLD:AAI2210
Kurzia|BACZP216-15|Guelph Lake|BOLD:AAG1894
Kurzia|BACZP286-15|Pond in the other side Guelph Lake|BOLD:AAG1894
Kurzia|BACZP343-15|Pond in the other side Guelph Lake|BOLD:AAG1894
Alona|BACZP015-15|Bacalar Lake, Cocalitos Sinkhole|BOLD:ACX5926
Alona|BACZP1430-16|Crawford lake|BOLD:AAD8055
Alona|BACZP1431-16|Crawford lake|BOLD:AAD8055
Alona|BACZP1483-16|Crawford lake, vegetation|BOLD:AAD8055
Aloninae|BACZP1281-16|Eel Lake|BOLD:AAD8055
Alona|BACZP1484-16|Crawford lake, vegetation|BOLD:AAD8055
Alona|BACZP1489-16|Crawford lake, vegetation|BOLD:AAD8055
Alona|BACZP1481-16|Crawford lake, vegetation|BOLD:AAD8055
Alona|BACZP1480-16|Crawford lake, vegetation|BOLD:AAD8055
Alona|BACZP1464-16|Crawford lake|BOLD:AAD8055
Alona|BACZP1453-16|Crawford lake|BOLD:AAD8055
Alona|BACZP1429-16|Crawford lake|BOLD:AAD8055
Alona|BACZP1428-16|Crawford lake|BOLD:AAD8055
Alona|BACZP1420-16|Crawford lake|BOLD:AAD8055
Alona|BACZP1419-16|Crawford lake|BOLD:AAD8055
Alona|BACZP1387-16|Crawford lake|BOLD:AAD8055
Alona|BACZP1386-16|Crawford lake|BOLD:AAD8055
Alona|BACZP1385-16|Crawford lake|BOLD:AAD8055
Alona|BACZP1510-16|Crawford lake, vegetation|BOLD:AAD8055
Alona|BACZP1512-16|Crawford lake, vegetation|BOLD:AAD8055
Alona|BACZP1514-16|Crawford lake, vegetation|BOLD:AAD8055
Aloninae|BACZP1119-16|Eel Lake|BOLD:AAF3595
Aloninae|BACZP1058-16|Eel Lake|BOLD:AAF3595
Aloninae|BACZP1192-16|Eel Lake|BOLD:AAF3595
Aloninae|BACZP1204-16|Eel Lake|BOLD:AAF3595
Aloninae|BACZP1280-16|Eel Lake|BOLD:AAF3595
Aloninae|BACZP1118-16|Eel Lake|BOLD:AAF3595
Aloninae|BACZP1258-16|Eel Lake|BOLD:AAF3595
Aloninae|BACZP1289-16|Eel Lake|BOLD:AAF3595
Aloninae|BACZP1059-16|Eel Lake|BOLD:AAF3595
Aloninae|BACZP1288-16|Eel Lake|BOLD:AAF3595
Aloninae|BACZP1688-16|Eel Lake|BOLD:AAF3595
Aloninae|BACZP1692-16|Eel Lake|BOLD:AAF3595
Aloninae|BACZP246-15|Pond in the other side Guelph Lake|BOLD:AAD8054
Aloninae|BACZP239-15|Pond in the other side Guelph Lake|BOLD:AAD8054
Aloninae|BACZP1366-16|Guelph Lake Swamp|BOLD:AAD8054
Aloninae|BACZP250-15|Pond in the other side Guelph Lake|BOLD:AAD8054
Aloninae|BACZP289-15|Pond in the other side Guelph Lake|BOLD:AAD8054
Aloninae|BACZP300-15|Pond in the other side Guelph Lake|BOLD:AAD8054
Aloninae|BACZP306-15|Pond in the other side Guelph Lake|BOLD:AAD8054
Aloninae|BACZP309-15|Pond in the other side Guelph Lake|BOLD:AAD8054
Aloninae|BACZP310-15|Pond in the other side Guelph Lake|BOLD:AAD8054
Aloninae|BACZP320-15|Pond in the other side Guelph Lake|BOLD:AAD8054
Alona|BACZP184-15|Bacalar Lake, Normal Sinkhole|BOLD:ACY0558
Alona|BACZP170-15|Bacalar Lake, Cocalitos sinkhole, light trap 1|BOLD:ACY0558
Alona|BACZP132-15|Bacalar Lake, Cocalitos Sinkhole|BOLD:ACY0558
Alona|BACZP655-16|Bacalar Lake, Cocalitos Sinkhole|BOLD:ACY0558
Alona|BACZP133-15|Bacalar Lake, Cocalitos Sinkhole|BOLD:ACY0558
Alona|BACZP465-16|Bacalar Lake, Cocalitos Sinkhole, light trap|BOLD:ACY0558
Alona|BACZP2254-16|Bacalar Lake, Cocalitos Sinkhole|BOLD:ACY0558
Leberis cf. davidi|BACZP446-16|Cacaxtla|BOLD:ACU2781
Alona|BACZP1140-16|Eel Lake|BOLD:AAW1782
Alona|BACZP1521-16|Eel Lake|BOLD:AAW1782
Alona|BACZP1570-16|Eel Lake|BOLD:AAW1782
Alona|BACZP1567-16|Eel Lake|BOLD:AAW1782
Alona|BACZP1553-16|Eel Lake|BOLD:AAW1782
Alona|BACZP1542-16|Eel Lake|BOLD:AAW1782
Alona|BACZP1264-16|Eel Lake|BOLD:AAW1782
Alona|BACZP1262-16|Eel Lake|BOLD:AAW1782
Alona|BACZP1569-16|Eel Lake|BOLD:AAW1782
Alona|BACZP1576-16|Eel Lake|BOLD:AAW1782
Alona|BACZP1283-16|Eel Lake|BOLD:AAW1782
Alona|BACZP1260-16|Eel Lake|BOLD:AAW1782
Alona|BACZP1548-16|Eel Lake|BOLD:AAW1782
Alona|BACZP1292-16|Eel Lake|BOLD:AAW1782
Alona|BACZP1544-16|Eel Lake|BOLD:AAW1782
Alona|BACZP1162-16|Eel Lake|BOLD:AAW1782
Alona circumfibriata|BACZP1146-16|Eel Lake|BOLD:AAW1782
Alona|BACZP1550-16|Eel Lake|BOLD:AAW1782
Alona|BACZP1575-16|Eel Lake|BOLD:AAW1782
Alona|BACZP1659-16|Fork of the Credit Pond|BOLD:AAW1782
Alona|BACZP1022-16|Guelph Lake Marsh|BOLD:AAW1782
Alona|BACZP1549-16|Eel Lake|BOLD:AAW1782
Alona|BACZP358-15|Guelph Lake|BOLD:AAW1782
Alona|BACZP1350-16|Guelph Lake Swamp|BOLD:AAW1782
Alona|BACZP1314-16|Guelph lake swamp|BOLD:AAW1782
Alona|BACZP1041-16|Guelph Lake Marsh|BOLD:AAW1782
Alona|BACZP1040-16|Guelph Lake Marsh|BOLD:AAW1782
Alona|BACZP1020-16|Guelph Lake Marsh|BOLD:AAW1782
Alona|BACZP1019-16|Guelph Lake Marsh|BOLD:AAW1782
Alona|BACZP253-15|Pond in the other side Guelph Lake|
Alona|BACZP1349-16|Guelph Lake Swamp|
Alona|BACZP686-16|Guelph Lake|
Alona|BACZP1021-16|Guelph Lake Marsh|BOLD:ADC2473
Alona|BACZP229-15|Guelph Lake|
Alona|BACZP364-15|Guelph Lake|
Alona|BACZP366-15|Guelph Lake|BOLD:AAW1782
Moina macrocopa|BACZP2263-16|Near Calderitas, pond in a road|BOLD:ACH4664
Moina macrocopa|BACZP2258-16|Near Calderitas, pond in a road|BOLD:ACH4664
Moina macrocopa|BACZP2266-16|Near Calderitas, pond in a road|BOLD:ACH4664
Moina macrocopa|BACZP2267-16|Near Calderitas, pond in a road|BOLD:ACH4664
Moinidae|BACZP2273-16|Near Calderitas, pond in a road|BOLD:ADD9104

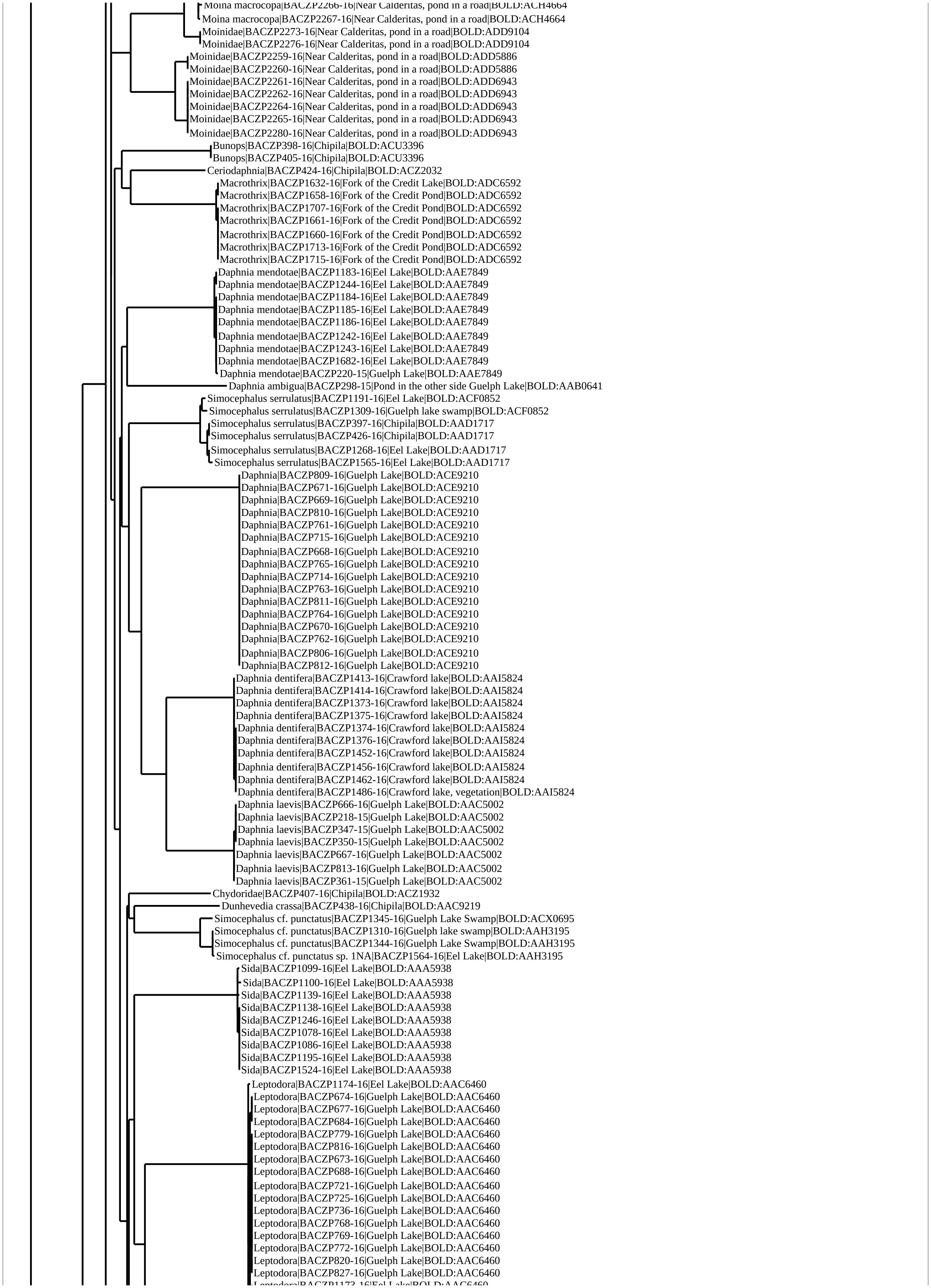
Moina macrocopa|BACZP2266-16|Near Calderitas, pond in a road|BOLD:ACH4664
Moina macrocopa|BACZP2267-16|Near Calderitas, pond in a road|BOLD:ACH4664
Moinidae|BACZP2273-16|Near Calderitas, pond in a road|BOLD:ADD9104
Moinidae|BACZP2276-16|Near Calderitas, pond in a road|BOLD:ADD9104
Moinidae|BACZP2259-16|Near Calderitas, pond in a road|BOLD:ADD5886
Moinidae|BACZP2260-16|Near Calderitas, pond in a road|BOLD:ADD5886
Moinidae|BACZP2261-16|Near Calderitas, pond in a road|BOLD:ADD6943
Moinidae|BACZP2262-16|Near Calderitas, pond in a road|BOLD:ADD6943
Moinidae|BACZP2264-16|Near Calderitas, pond in a road|BOLD:ADD6943
Moinidae|BACZP2265-16|Near Calderitas, pond in a road|BOLD:ADD6943
Moinidae|BACZP2280-16|Near Calderitas, pond in a road|BOLD:ADD6943
Bunops|BACZP398-16|Chipila|BOLD:ACU3396
Bunops|BACZP405-16|Chipila|BOLD:ACU3396
Ceriodaphnia|BACZP424-16|Chipila|BOLD:ACZ2032
Macrothrix|BACZP1632-16|Fork of the Credit Lake|BOLD:ADC6592
Macrothrix|BACZP1658-16|Fork of the Credit Pond|BOLD:ADC6592
Macrothrix|BACZP1707-16|Fork of the Credit Pond|BOLD:ADC6592
Macrothrix|BACZP1661-16|Fork of the Credit Pond|BOLD:ADC6592
Macrothrix|BACZP1660-16|Fork of the Credit Pond|BOLD:ADC6592
Macrothrix|BACZP1713-16|Fork of the Credit Pond|BOLD:ADC6592
Macrothrix|BACZP1715-16|Fork of the Credit Pond|BOLD:ADC6592
Daphnia mendotae|BACZP1183-16|Eel Lake|BOLD:AAE7849
Daphnia mendotae|BACZP1244-16|Eel Lake|BOLD:AAE7849
Daphnia mendotae|BACZP1184-16|Eel Lake|BOLD:AAE7849
Daphnia mendotae|BACZP1185-16|Eel Lake|BOLD:AAE7849
Daphnia mendotae|BACZP1186-16|Eel Lake|BOLD:AAE7849
Daphnia mendotae|BACZP1242-16|Eel Lake|BOLD:AAE7849
Daphnia mendotae|BACZP1243-16|Eel Lake|BOLD:AAE7849
Daphnia mendotae|BACZP1682-16|Eel Lake|BOLD:AAE7849
Daphnia mendotae|BACZP220-15|Guelph Lake|BOLD:AAE7849
Daphnia ambigua|BACZP298-15|Pond in the other side Guelph Lake|BOLD:AAB0641
Simocephalus serrulatus|BACZP1191-16|Eel Lake|BOLD:ACF0852
Simocephalus serrulatus|BACZP1309-16|Guelph lake swamp|BOLD:ACF0852
Simocephalus serrulatus|BACZP397-16|Chipila|BOLD:AAD1717
Simocephalus serrulatus|BACZP426-16|Chipila|BOLD:AAD1717
Simocephalus serrulatus|BACZP1268-16|Eel Lake|BOLD:AAD1717
Simocephalus serrulatus|BACZP1565-16|Eel Lake|BOLD:AAD1717
Daphnia|BACZP809-16|Guelph Lake|BOLD:ACE9210
Daphnia|BACZP671-16|Guelph Lake|BOLD:ACE9210
Daphnia|BACZP669-16|Guelph Lake|BOLD:ACE9210
Daphnia|BACZP810-16|Guelph Lake|BOLD:ACE9210
Daphnia|BACZP761-16|Guelph Lake|BOLD:ACE9210
Daphnia|BACZP715-16|Guelph Lake|BOLD:ACE9210
Daphnia|BACZP668-16|Guelph Lake|BOLD:ACE9210
Daphnia|BACZP765-16|Guelph Lake|BOLD:ACE9210
Daphnia|BACZP714-16|Guelph Lake|BOLD:ACE9210
Daphnia|BACZP763-16|Guelph Lake|BOLD:ACE9210
Daphnia|BACZP811-16|Guelph Lake|BOLD:ACE9210
Daphnia|BACZP764-16|Guelph Lake|BOLD:ACE9210
Daphnia|BACZP670-16|Guelph Lake|BOLD:ACE9210
Daphnia|BACZP762-16|Guelph Lake|BOLD:ACE9210
Daphnia|BACZP806-16|Guelph Lake|BOLD:ACE9210
Daphnia|BACZP812-16|Guelph Lake|BOLD:ACE9210
Daphnia dentifera|BACZP1413-16|Crawford lake|BOLD:AAI5824
Daphnia dentifera|BACZP1414-16|Crawford lake|BOLD:AAI5824
Daphnia dentifera|BACZP1373-16|Crawford lake|BOLD:AAI5824
Daphnia dentifera|BACZP1375-16|Crawford lake|BOLD:AAI5824
Daphnia dentifera|BACZP1374-16|Crawford lake|BOLD:AAI5824
Daphnia dentifera|BACZP1376-16|Crawford lake|BOLD:AAI5824
Daphnia dentifera|BACZP1452-16|Crawford lake|BOLD:AAI5824
Daphnia dentifera|BACZP1456-16|Crawford lake|BOLD:AAI5824
Daphnia dentifera|BACZP1462-16|Crawford lake|BOLD:AAI5824
Daphnia dentifera|BACZP1486-16|Crawford lake, vegetation|BOLD:AAI5824
Daphnia laevis|BACZP666-16|Guelph Lake|BOLD:AAC5002
Daphnia laevis|BACZP218-15|Guelph Lake|BOLD:AAC5002
Daphnia laevis|BACZP347-15|Guelph Lake|BOLD:AAC5002
Daphnia laevis|BACZP350-15|Guelph Lake|BOLD:AAC5002
Daphnia laevis|BACZP667-16|Guelph Lake|BOLD:AAC5002
Daphnia laevis|BACZP813-16|Guelph Lake|BOLD:AAC5002
Daphnia laevis|BACZP361-15|Guelph Lake|BOLD:AAC5002
Chydoridae|BACZP407-16|Chipila|BOLD:ACZ1932
Dunhevedia crassa|BACZP438-16|Chipila|BOLD:AAC9219
Simocephalus cf. punctatus|BACZP1345-16|Guelph Lake Swamp|BOLD:ACX0695
Simocephalus cf. punctatus|BACZP1310-16|Guelph lake swamp|BOLD:AAH3195
Simocephalus cf. punctatus|BACZP1344-16|Guelph Lake Swamp|BOLD:AAH3195
Simocephalus cf. punctatus sp. 1NA|BACZP1564-16|Eel Lake|BOLD:AAH3195
Sida|BACZP1099-16|Eel Lake|BOLD:AAA5938
Sida|BACZP1100-16|Eel Lake|BOLD:AAA5938
Sida|BACZP1139-16|Eel Lake|BOLD:AAA5938
Sida|BACZP1138-16|Eel Lake|BOLD:AAA5938
Sida|BACZP1246-16|Eel Lake|BOLD:AAA5938
Sida|BACZP1078-16|Eel Lake|BOLD:AAA5938
Sida|BACZP1086-16|Eel Lake|BOLD:AAA5938
Sida|BACZP1195-16|Eel Lake|BOLD:AAA5938
Sida|BACZP1524-16|Eel Lake|BOLD:AAA5938
Leptodora|BACZP1174-16|Eel Lake|BOLD:AAC6460
Leptodora|BACZP674-16|Guelph Lake|BOLD:AAC6460
Leptodora|BACZP677-16|Guelph Lake|BOLD:AAC6460
Leptodora|BACZP684-16|Guelph Lake|BOLD:AAC6460
Leptodora|BACZP779-16|Guelph Lake|BOLD:AAC6460
Leptodora|BACZP816-16|Guelph Lake|BOLD:AAC6460
Leptodora|BACZP673-16|Guelph Lake|BOLD:AAC6460
Leptodora|BACZP688-16|Guelph Lake|BOLD:AAC6460
Leptodora|BACZP721-16|Guelph Lake|BOLD:AAC6460
Leptodora|BACZP725-16|Guelph Lake|BOLD:AAC6460
Leptodora|BACZP736-16|Guelph Lake|BOLD:AAC6460
Leptodora|BACZP768-16|Guelph Lake|BOLD:AAC6460
Leptodora|BACZP769-16|Guelph Lake|BOLD:AAC6460
Leptodora|BACZP772-16|Guelph Lake|BOLD:AAC6460
Leptodora|BACZP820-16|Guelph Lake|BOLD:AAC6460
Leptodora|BACZP827-16|Guelph Lake|BOLD:AAC6460

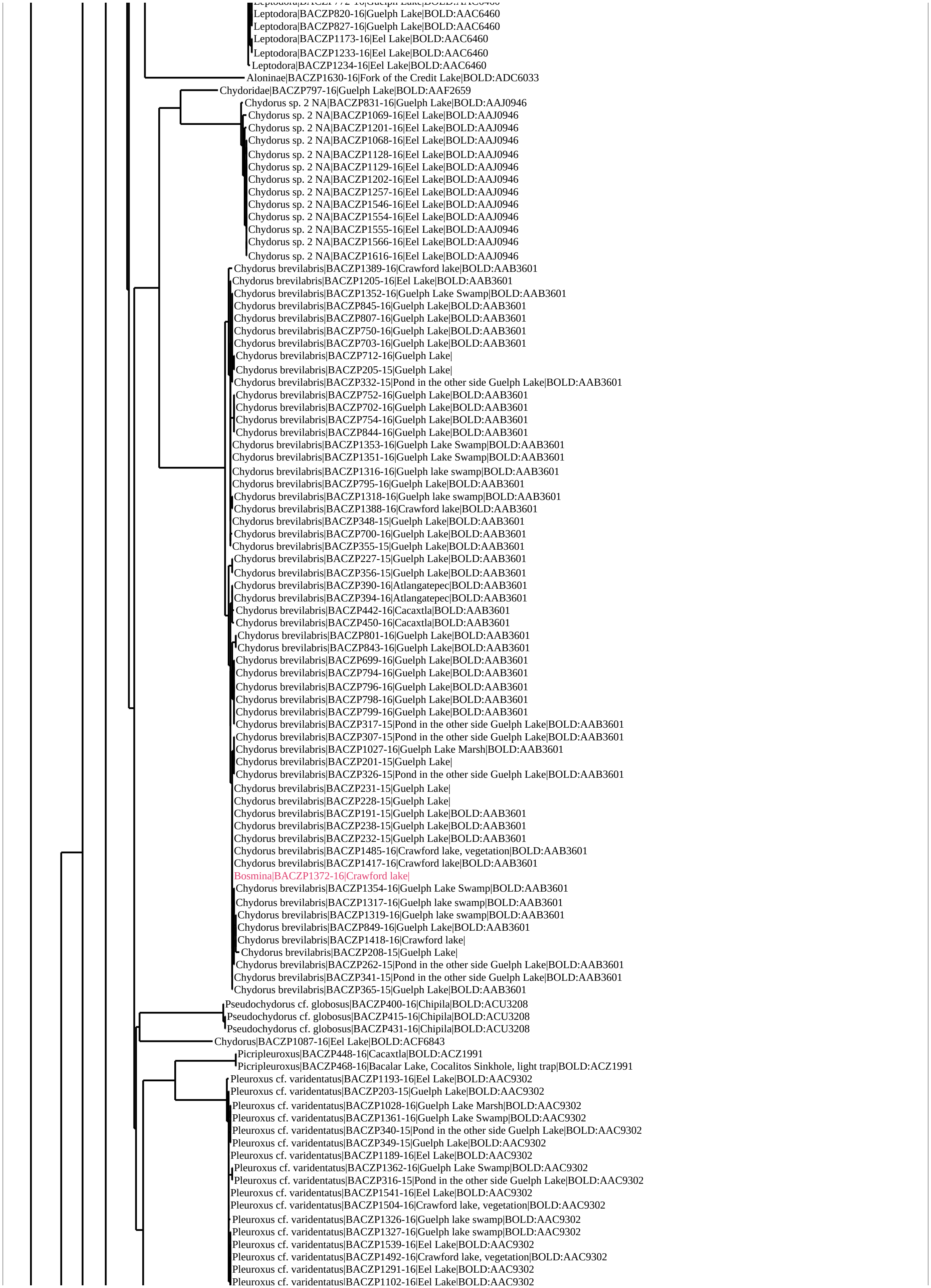

Leptodora|BACZP820-16|Guelph Lake|BOLD:AAC6460
Leptodora|BACZP827-16|Guelph Lake|BOLD:AAC6460
Leptodora|BACZP1173-16|Eel Lake|BOLD:AAC6460
Leptodora|BACZP1233-16|Eel Lake|BOLD:AAC6460
Leptodora|BACZP1234-16|Eel Lake|BOLD:AAC6460
Aloninae|BACZP1630-16|Fork of the Credit Lake|BOLD:ADC6033
Chydoridae|BACZP797-16|Guelph Lake|BOLD:AAF2659
Chydorus sp. 2 NA|BACZP831-16|Guelph Lake|BOLD:AAJ0946
Chydorus sp. 2 NA|BACZP1069-16|Eel Lake|BOLD:AAJ0946
Chydorus sp. 2 NA|BACZP1201-16|Eel Lake|BOLD:AAJ0946
Chydorus sp. 2 NA|BACZP1068-16|Eel Lake|BOLD:AAJ0946
Chydorus sp. 2 NA|BACZP1128-16|Eel Lake|BOLD:AAJ0946
Chydorus sp. 2 NA|BACZP1129-16|Eel Lake|BOLD:AAJ0946
Chydorus sp. 2 NA|BACZP1202-16|Eel Lake|BOLD:AAJ0946
Chydorus sp. 2 NA|BACZP1257-16|Eel Lake|BOLD:AAJ0946
Chydorus sp. 2 NA|BACZP1546-16|Eel Lake|BOLD:AAJ0946
Chydorus sp. 2 NA|BACZP1554-16|Eel Lake|BOLD:AAJ0946
Chydorus sp. 2 NA|BACZP1555-16|Eel Lake|BOLD:AAJ0946
Chydorus sp. 2 NA|BACZP1566-16|Eel Lake|BOLD:AAJ0946
Chydorus sp. 2 NA|BACZP1616-16|Eel Lake|BOLD:AAJ0946
Chydorus brevilabris|BACZP1389-16|Crawford lake|BOLD:AAB3601
Chydorus brevilabris|BACZP1205-16|Eel Lake|BOLD:AAB3601
Chydorus brevilabris|BACZP1352-16|Guelph Lake Swamp|BOLD:AAB3601
Chydorus brevilabris|BACZP845-16|Guelph Lake|BOLD:AAB3601
Chydorus brevilabris|BACZP807-16|Guelph Lake|BOLD:AAB3601
Chydorus brevilabris|BACZP750-16|Guelph Lake|BOLD:AAB3601
Chydorus brevilabris|BACZP703-16|Guelph Lake|BOLD:AAB3601
Chydorus brevilabris|BACZP712-16|Guelph Lake|
Chydorus brevilabris|BACZP205-15|Guelph Lake|
Chydorus brevilabris|BACZP332-15|Pond in the other side Guelph Lake|BOLD:AAB3601
Chydorus brevilabris|BACZP752-16|Guelph Lake|BOLD:AAB3601
Chydorus brevilabris|BACZP702-16|Guelph Lake|BOLD:AAB3601
Chydorus brevilabris|BACZP754-16|Guelph Lake|BOLD:AAB3601
Chydorus brevilabris|BACZP844-16|Guelph Lake|BOLD:AAB3601
Chydorus brevilabris|BACZP1353-16|Guelph Lake Swamp|BOLD:AAB3601
Chydorus brevilabris|BACZP1351-16|Guelph Lake Swamp|BOLD:AAB3601
Chydorus brevilabris|BACZP1316-16|Guelph lake swamp|BOLD:AAB3601
Chydorus brevilabris|BACZP795-16|Guelph Lake|BOLD:AAB3601
Chydorus brevilabris|BACZP1318-16|Guelph lake swamp|BOLD:AAB3601
Chydorus brevilabris|BACZP1388-16|Crawford lake|BOLD:AAB3601
Chydorus brevilabris|BACZP348-15|Guelph Lake|BOLD:AAB3601
Chydorus brevilabris|BACZP700-16|Guelph Lake|BOLD:AAB3601
Chydorus brevilabris|BACZP355-15|Guelph Lake|BOLD:AAB3601
Chydorus brevilabris|BACZP227-15|Guelph Lake|BOLD:AAB3601
Chydorus brevilabris|BACZP356-15|Guelph Lake|BOLD:AAB3601
Chydorus brevilabris|BACZP390-16|Atlangatepec|BOLD:AAB3601
Chydorus brevilabris|BACZP394-16|Atlangatepec|BOLD:AAB3601
Chydorus brevilabris|BACZP442-16|Cacaxtla|BOLD:AAB3601
Chydorus brevilabris|BACZP450-16|Cacaxtla|BOLD:AAB3601
Chydorus brevilabris|BACZP801-16|Guelph Lake|BOLD:AAB3601
Chydorus brevilabris|BACZP843-16|Guelph Lake|BOLD:AAB3601
Chydorus brevilabris|BACZP699-16|Guelph Lake|BOLD:AAB3601
Chydorus brevilabris|BACZP794-16|Guelph Lake|BOLD:AAB3601
Chydorus brevilabris|BACZP796-16|Guelph Lake|BOLD:AAB3601
Chydorus brevilabris|BACZP798-16|Guelph Lake|BOLD:AAB3601
Chydorus brevilabris|BACZP799-16|Guelph Lake|BOLD:AAB3601
Chydorus brevilabris|BACZP317-15|Pond in the other side Guelph Lake|BOLD:AAB3601
Chydorus brevilabris|BACZP307-15|Pond in the other side Guelph Lake|BOLD:AAB3601
Chydorus brevilabris|BACZP1027-16|Guelph Lake Marsh|BOLD:AAB3601
Chydorus brevilabris|BACZP201-15|Guelph Lake|
Chydorus brevilabris|BACZP326-15|Pond in the other side Guelph Lake|BOLD:AAB3601
Chydorus brevilabris|BACZP231-15|Guelph Lake|
Chydorus brevilabris|BACZP228-15|Guelph Lake|
Chydorus brevilabris|BACZP191-15|Guelph Lake|BOLD:AAB3601
Chydorus brevilabris|BACZP238-15|Guelph Lake|BOLD:AAB3601
Chydorus brevilabris|BACZP232-15|Guelph Lake|BOLD:AAB3601
Chydorus brevilabris|BACZP1485-16|Crawford lake, vegetation|BOLD:AAB3601
Chydorus brevilabris|BACZP1417-16|Crawford lake|BOLD:AAB3601
Bosmina|BACZP1372-16|Crawford lake|
Chydorus brevilabris|BACZP1354-16|Guelph Lake Swamp|BOLD:AAB3601
Chydorus brevilabris|BACZP1317-16|Guelph lake swamp|BOLD:AAB3601
Chydorus brevilabris|BACZP1319-16|Guelph lake swamp|BOLD:AAB3601
Chydorus brevilabris|BACZP849-16|Guelph Lake|BOLD:AAB3601
Chydorus brevilabris|BACZP1418-16|Crawford lake|
Chydorus brevilabris|BACZP208-15|Guelph Lake|
Chydorus brevilabris|BACZP262-15|Pond in the other side Guelph Lake|BOLD:AAB3601
Chydorus brevilabris|BACZP341-15|Pond in the other side Guelph Lake|BOLD:AAB3601
Chydorus brevilabris|BACZP365-15|Guelph Lake|BOLD:AAB3601
Pseudochydorus cf. globosus|BACZP400-16|Chipila|BOLD:ACU3208
Pseudochydorus cf. globosus|BACZP415-16|Chipila|BOLD:ACU3208
Pseudochydorus cf. globosus|BACZP431-16|Chipila|BOLD:ACU3208
Chydorus|BACZP1087-16|Eel Lake|BOLD:ACF6843
Picripleuroxus|BACZP448-16|Cacaxtla|BOLD:ACZ1991
Picripleuroxus|BACZP468-16|Bacalar Lake, Cocalitos Sinkhole, light trap|BOLD:ACZ1991
Pleuroxus cf. varidentatus|BACZP1193-16|Eel Lake|BOLD:AAC9302
Pleuroxus cf. varidentatus|BACZP203-15|Guelph Lake|BOLD:AAC9302
Pleuroxus cf. varidentatus|BACZP1028-16|Guelph Lake Marsh|BOLD:AAC9302
Pleuroxus cf. varidentatus|BACZP1361-16|Guelph Lake Swamp|BOLD:AAC9302
Pleuroxus cf. varidentatus|BACZP340-15|Pond in the other side Guelph Lake|BOLD:AAC9302
Pleuroxus cf. varidentatus|BACZP349-15|Guelph Lake|BOLD:AAC9302
Pleuroxus cf. varidentatus|BACZP1189-16|Eel Lake|BOLD:AAC9302
Pleuroxus cf. varidentatus|BACZP1362-16|Guelph Lake Swamp|BOLD:AAC9302
Pleuroxus cf. varidentatus|BACZP316-15|Pond in the other side Guelph Lake|BOLD:AAC9302
Pleuroxus cf. varidentatus|BACZP1541-16|Eel Lake|BOLD:AAC9302
Pleuroxus cf. varidentatus|BACZP1504-16|Crawford lake, vegetation|BOLD:AAC9302
Pleuroxus cf. varidentatus|BACZP1326-16|Guelph lake swamp|BOLD:AAC9302
Pleuroxus cf. varidentatus|BACZP1327-16|Guelph lake swamp|BOLD:AAC9302
Pleuroxus cf. varidentatus|BACZP1539-16|Eel Lake|BOLD:AAC9302
Pleuroxus cf. varidentatus|BACZP1492-16|Crawford lake, vegetation|BOLD:AAC9302
Pleuroxus cf. varidentatus|BACZP1291-16|Eel Lake|BOLD:AAC9302
Pleuroxus cf. varidentatus|BACZP1102-16|Eel Lake|BOLD:AAC9302

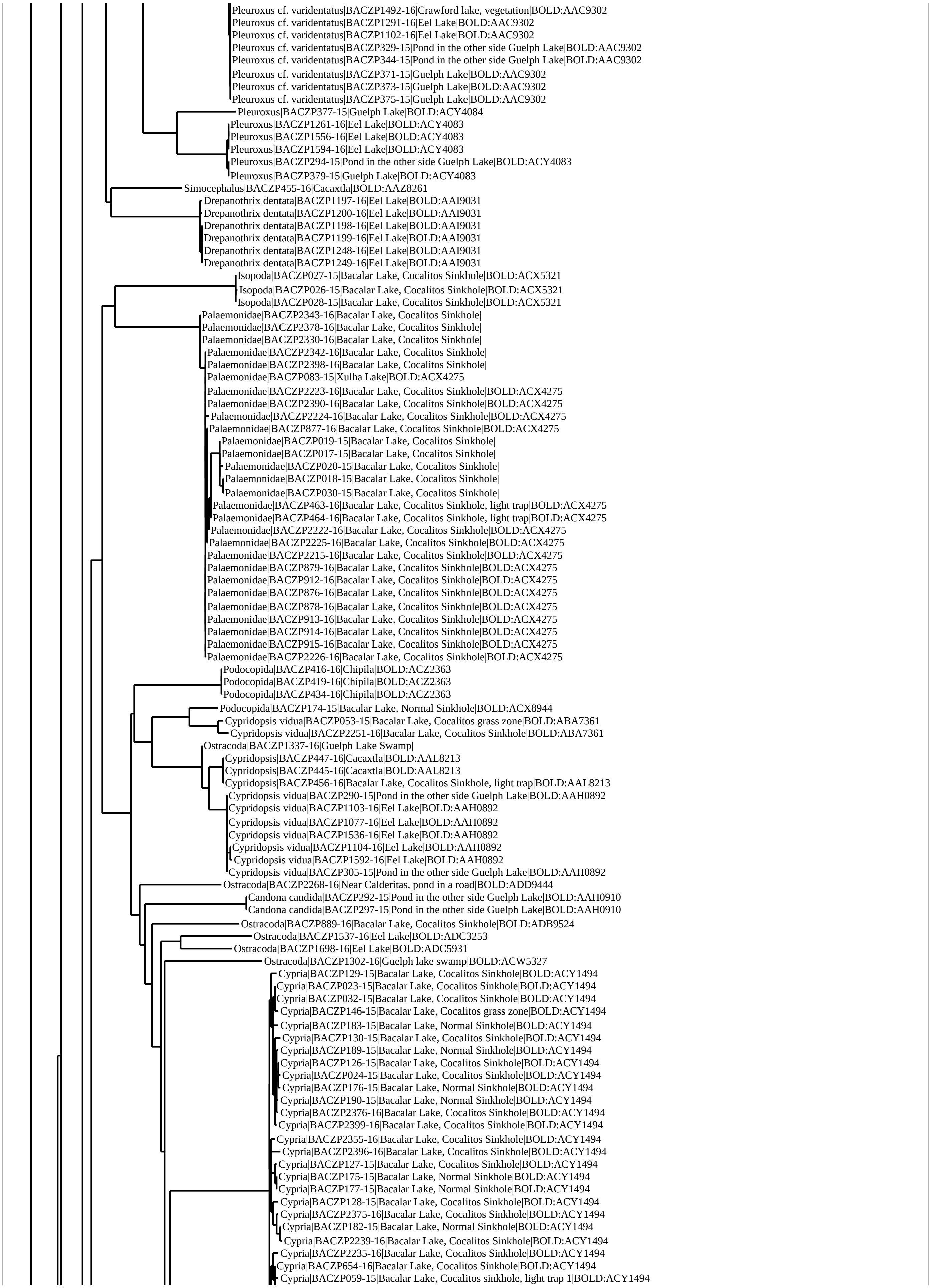
Pleuroxus cf. varidentatus|BACZP1492-16|Crawford lake, vegetation|BOLD:AAC9302
Pleuroxus cf. varidentatus|BACZP1291-16|Eel Lake|BOLD:AAC9302
Pleuroxus cf. varidentatus|BACZP1102-16|Eel Lake|BOLD:AAC9302
Pleuroxus cf. varidentatus|BACZP329-15|Pond in the other side Guelph Lake|BOLD:AAC9302
Pleuroxus cf. varidentatus|BACZP344-15|Pond in the other side Guelph Lake|BOLD:AAC9302
Pleuroxus cf. varidentatus|BACZP371-15|Guelph Lake|BOLD:AAC9302
Pleuroxus cf. varidentatus|BACZP373-15|Guelph Lake|BOLD:AAC9302
Pleuroxus cf. varidentatus|BACZP375-15|Guelph Lake|BOLD:AAC9302
Pleuroxus|BACZP377-15|Guelph Lake|BOLD:ACY4084
Pleuroxus|BACZP1261-16|Eel Lake|BOLD:ACY4083
Pleuroxus|BACZP1556-16|Eel Lake|BOLD:ACY4083
Pleuroxus|BACZP1594-16|Eel Lake|BOLD:ACY4083
Pleuroxus|BACZP294-15|Pond in the other side Guelph Lake|BOLD:ACY4083
Pleuroxus|BACZP379-15|Guelph Lake|BOLD:ACY4083
Simocephalus|BACZP455-16|Cacaxtla|BOLD:AAZ8261
Drepanothrix dentata|BACZP1197-16|Eel Lake|BOLD:AAI9031
Drepanothrix dentata|BACZP1200-16|Eel Lake|BOLD:AAI9031
Drepanothrix dentata|BACZP1198-16|Eel Lake|BOLD:AAI9031
Drepanothrix dentata|BACZP1199-16|Eel Lake|BOLD:AAI9031
Drepanothrix dentata|BACZP1248-16|Eel Lake|BOLD:AAI9031
Drepanothrix dentata|BACZP1249-16|Eel Lake|BOLD:AAI9031
Isopoda|BACZP027-15|Bacalar Lake, Cocalitos Sinkhole|BOLD:ACX5321
Isopoda|BACZP026-15|Bacalar Lake, Cocalitos Sinkhole|BOLD:ACX5321
Isopoda|BACZP028-15|Bacalar Lake, Cocalitos Sinkhole|BOLD:ACX5321
Palaemonidae|BACZP2343-16|Bacalar Lake, Cocalitos Sinkhole|
Palaemonidae|BACZP2378-16|Bacalar Lake, Cocalitos Sinkhole|
Palaemonidae|BACZP2330-16|Bacalar Lake, Cocalitos Sinkhole|
Palaemonidae|BACZP2342-16|Bacalar Lake, Cocalitos Sinkhole|
Palaemonidae|BACZP2398-16|Bacalar Lake, Cocalitos Sinkhole|
Palaemonidae|BACZP083-15|Xulha Lake|BOLD:ACX4275
Palaemonidae|BACZP2223-16|Bacalar Lake, Cocalitos Sinkhole|BOLD:ACX4275
Palaemonidae|BACZP2390-16|Bacalar Lake, Cocalitos Sinkhole|BOLD:ACX4275
Palaemonidae|BACZP2224-16|Bacalar Lake, Cocalitos Sinkhole|BOLD:ACX4275
Palaemonidae|BACZP877-16|Bacalar Lake, Cocalitos Sinkhole|BOLD:ACX4275
Palaemonidae|BACZP019-15|Bacalar Lake, Cocalitos Sinkhole|
Palaemonidae|BACZP017-15|Bacalar Lake, Cocalitos Sinkhole|
Palaemonidae|BACZP020-15|Bacalar Lake, Cocalitos Sinkhole|
Palaemonidae|BACZP018-15|Bacalar Lake, Cocalitos Sinkhole|
Palaemonidae|BACZP030-15|Bacalar Lake, Cocalitos Sinkhole|
Palaemonidae|BACZP463-16|Bacalar Lake, Cocalitos Sinkhole, light trap|BOLD:ACX4275
Palaemonidae|BACZP464-16|Bacalar Lake, Cocalitos Sinkhole, light trap|BOLD:ACX4275
Palaemonidae|BACZP2222-16|Bacalar Lake, Cocalitos Sinkhole|BOLD:ACX4275
Palaemonidae|BACZP2225-16|Bacalar Lake, Cocalitos Sinkhole|BOLD:ACX4275
Palaemonidae|BACZP2215-16|Bacalar Lake, Cocalitos Sinkhole|BOLD:ACX4275
Palaemonidae|BACZP879-16|Bacalar Lake, Cocalitos Sinkhole|BOLD:ACX4275
Palaemonidae|BACZP912-16|Bacalar Lake, Cocalitos Sinkhole|BOLD:ACX4275
Palaemonidae|BACZP876-16|Bacalar Lake, Cocalitos Sinkhole|BOLD:ACX4275
Palaemonidae|BACZP878-16|Bacalar Lake, Cocalitos Sinkhole|BOLD:ACX4275
Palaemonidae|BACZP913-16|Bacalar Lake, Cocalitos Sinkhole|BOLD:ACX4275
Palaemonidae|BACZP914-16|Bacalar Lake, Cocalitos Sinkhole|BOLD:ACX4275
Palaemonidae|BACZP915-16|Bacalar Lake, Cocalitos Sinkhole|BOLD:ACX4275
Palaemonidae|BACZP2226-16|Bacalar Lake, Cocalitos Sinkhole|BOLD:ACX4275
Podocopida|BACZP416-16|Chipila|BOLD:ACZ2363
Podocopida|BACZP419-16|Chipila|BOLD:ACZ2363
Podocopida|BACZP434-16|Chipila|BOLD:ACZ2363
Podocopida|BACZP174-15|Bacalar Lake, Normal Sinkhole|BOLD:ACX8944
Cypridopsis vidua|BACZP053-15|Bacalar Lake, Cocalitos grass zone|BOLD:ABA7361
Cypridopsis vidua|BACZP2251-16|Bacalar Lake, Cocalitos Sinkhole|BOLD:ABA7361
Ostracoda|BACZP1337-16|Guelph Lake Swamp|
Cypridopsis|BACZP447-16|Cacaxtla|BOLD:AAL8213
Cypridopsis|BACZP445-16|Cacaxtla|BOLD:AAL8213
Cypridopsis|BACZP456-16|Bacalar Lake, Cocalitos Sinkhole, light trap|BOLD:AAL8213
Cypridopsis vidua|BACZP290-15|Pond in the other side Guelph Lake|BOLD:AAH0892
Cypridopsis vidua|BACZP1103-16|Eel Lake|BOLD:AAH0892
Cypridopsis vidua|BACZP1077-16|Eel Lake|BOLD:AAH0892
Cypridopsis vidua|BACZP1536-16|Eel Lake|BOLD:AAH0892
Cypridopsis vidua|BACZP1104-16|Eel Lake|BOLD:AAH0892
Cypridopsis vidua|BACZP1592-16|Eel Lake|BOLD:AAH0892
Cypridopsis vidua|BACZP305-15|Pond in the other side Guelph Lake|BOLD:AAH0892
Ostracoda|BACZP2268-16|Near Calderitas, pond in a road|BOLD:ADD9444
Candona candida|BACZP292-15|Pond in the other side Guelph Lake|BOLD:AAH0910
Candona candida|BACZP297-15|Pond in the other side Guelph Lake|BOLD:AAH0910
Ostracoda|BACZP889-16|Bacalar Lake, Cocalitos Sinkhole|BOLD:ADB9524
Ostracoda|BACZP1537-16|Eel Lake|BOLD:ADC3253
Ostracoda|BACZP1698-16|Eel Lake|BOLD:ADC5931
Ostracoda|BACZP1302-16|Guelph lake swamp|BOLD:ACW5327
Cypria|BACZP129-15|Bacalar Lake, Cocalitos Sinkhole|BOLD:ACY1494
Cypria|BACZP023-15|Bacalar Lake, Cocalitos Sinkhole|BOLD:ACY1494
Cypria|BACZP032-15|Bacalar Lake, Cocalitos Sinkhole|BOLD:ACY1494
Cypria|BACZP146-15|Bacalar Lake, Cocalitos grass zone|BOLD:ACY1494
Cypria|BACZP183-15|Bacalar Lake, Normal Sinkhole|BOLD:ACY1494
Cypria|BACZP130-15|Bacalar Lake, Cocalitos Sinkhole|BOLD:ACY1494
Cypria|BACZP189-15|Bacalar Lake, Normal Sinkhole|BOLD:ACY1494
Cypria|BACZP126-15|Bacalar Lake, Cocalitos Sinkhole|BOLD:ACY1494
Cypria|BACZP024-15|Bacalar Lake, Cocalitos Sinkhole|BOLD:ACY1494
Cypria|BACZP176-15|Bacalar Lake, Normal Sinkhole|BOLD:ACY1494
Cypria|BACZP190-15|Bacalar Lake, Normal Sinkhole|BOLD:ACY1494
Cypria|BACZP2376-16|Bacalar Lake, Cocalitos Sinkhole|BOLD:ACY1494
Cypria|BACZP2399-16|Bacalar Lake, Cocalitos Sinkhole|BOLD:ACY1494
Cypria|BACZP2355-16|Bacalar Lake, Cocalitos Sinkhole|BOLD:ACY1494
Cypria|BACZP2396-16|Bacalar Lake, Cocalitos Sinkhole|BOLD:ACY1494
Cypria|BACZP127-15|Bacalar Lake, Cocalitos Sinkhole|BOLD:ACY1494
Cypria|BACZP175-15|Bacalar Lake, Normal Sinkhole|BOLD:ACY1494
Cypria|BACZP177-15|Bacalar Lake, Normal Sinkhole|BOLD:ACY1494
Cypria|BACZP128-15|Bacalar Lake, Cocalitos Sinkhole|BOLD:ACY1494
Cypria|BACZP2375-16|Bacalar Lake, Cocalitos Sinkhole|BOLD:ACY1494
Cypria|BACZP182-15|Bacalar Lake, Normal Sinkhole|BOLD:ACY1494
Cypria|BACZP2239-16|Bacalar Lake, Cocalitos Sinkhole|BOLD:ACY1494
Cypria|BACZP2235-16|Bacalar Lake, Cocalitos Sinkhole|BOLD:ACY1494
Cypria|BACZP654-16|Bacalar Lake, Cocalitos Sinkhole|BOLD:ACY1494
Cypria|BACZP059-15|Bacalar Lake, Cocalitos sinkhole, light trap 1|BOLD:ACY1494

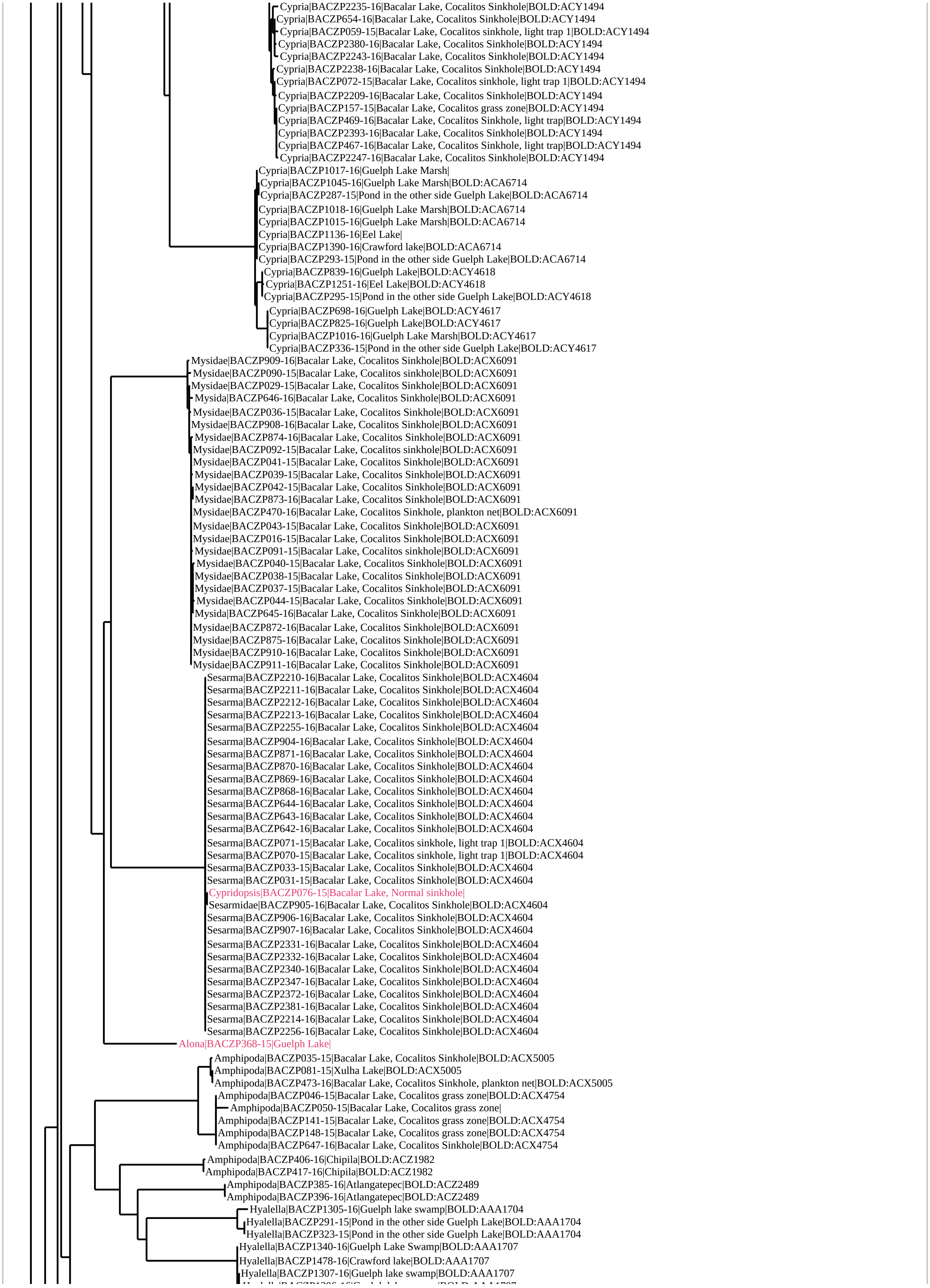
Cypria|BACZP2235-16|Bacalar Lake, Cocalitos Sinkhole|BOLD:ACY1494
Cypria|BACZP654-16|Bacalar Lake, Cocalitos Sinkhole|BOLD:ACY1494
Cypria|BACZP059-15|Bacalar Lake, Cocalitos sinkhole, light trap 1|BOLD:ACY1494
Cypria|BACZP2380-16|Bacalar Lake, Cocalitos Sinkhole|BOLD:ACY1494
Cypria|BACZP2243-16|Bacalar Lake, Cocalitos Sinkhole|BOLD:ACY1494
Cypria|BACZP2238-16|Bacalar Lake, Cocalitos Sinkhole|BOLD:ACY1494
Cypria|BACZP072-15|Bacalar Lake, Cocalitos sinkhole, light trap 1|BOLD:ACY1494
Cypria|BACZP2209-16|Bacalar Lake, Cocalitos Sinkhole|BOLD:ACY1494
Cypria|BACZP157-15|Bacalar Lake, Cocalitos grass zone|BOLD:ACY1494
Cypria|BACZP469-16|Bacalar Lake, Cocalitos Sinkhole, light trap|BOLD:ACY1494
Cypria|BACZP2393-16|Bacalar Lake, Cocalitos Sinkhole|BOLD:ACY1494
Cypria|BACZP467-16|Bacalar Lake, Cocalitos Sinkhole, light trap|BOLD:ACY1494
Cypria|BACZP2247-16|Bacalar Lake, Cocalitos Sinkhole|BOLD:ACY1494
Cypria|BACZP1017-16|Guelph Lake Marsh|
Cypria|BACZP1045-16|Guelph Lake Marsh|BOLD:ACA6714
Cypria|BACZP287-15|Pond in the other side Guelph Lake|BOLD:ACA6714
Cypria|BACZP1018-16|Guelph Lake Marsh|BOLD:ACA6714
Cypria|BACZP1015-16|Guelph Lake Marsh|BOLD:ACA6714
Cypria|BACZP1136-16|Eel Lake|
Cypria|BACZP1390-16|Crawford lake|BOLD:ACA6714
Cypria|BACZP293-15|Pond in the other side Guelph Lake|BOLD:ACA6714
Cypria|BACZP839-16|Guelph Lake|BOLD:ACY4618
Cypria|BACZP1251-16|Eel Lake|BOLD:ACY4618
Cypria|BACZP295-15|Pond in the other side Guelph Lake|BOLD:ACY4618
Cypria|BACZP698-16|Guelph Lake|BOLD:ACY4617
Cypria|BACZP825-16|Guelph Lake|BOLD:ACY4617
Cypria|BACZP1016-16|Guelph Lake Marsh|BOLD:ACY4617
Cypria|BACZP336-15|Pond in the other side Guelph Lake|BOLD:ACY4617
Mysidae|BACZP909-16|Bacalar Lake, Cocalitos Sinkhole|BOLD:ACX6091
Mysidae|BACZP090-15|Bacalar Lake, Cocalitos sinkhole|BOLD:ACX6091
Mysidae|BACZP029-15|Bacalar Lake, Cocalitos Sinkhole|BOLD:ACX6091
Mysida|BACZP646-16|Bacalar Lake, Cocalitos Sinkhole|BOLD:ACX6091
Mysidae|BACZP036-15|Bacalar Lake, Cocalitos Sinkhole|BOLD:ACX6091
Mysidae|BACZP908-16|Bacalar Lake, Cocalitos Sinkhole|BOLD:ACX6091
Mysidae|BACZP874-16|Bacalar Lake, Cocalitos Sinkhole|BOLD:ACX6091
Mysidae|BACZP092-15|Bacalar Lake, Cocalitos sinkhole|BOLD:ACX6091
Mysidae|BACZP041-15|Bacalar Lake, Cocalitos Sinkhole|BOLD:ACX6091
Mysidae|BACZP039-15|Bacalar Lake, Cocalitos Sinkhole|BOLD:ACX6091
Mysidae|BACZP042-15|Bacalar Lake, Cocalitos Sinkhole|BOLD:ACX6091
Mysidae|BACZP873-16|Bacalar Lake, Cocalitos Sinkhole|BOLD:ACX6091
Mysidae|BACZP470-16|Bacalar Lake, Cocalitos Sinkhole, plankton net|BOLD:ACX6091
Mysidae|BACZP043-15|Bacalar Lake, Cocalitos Sinkhole|BOLD:ACX6091
Mysidae|BACZP016-15|Bacalar Lake, Cocalitos Sinkhole|BOLD:ACX6091
Mysidae|BACZP091-15|Bacalar Lake, Cocalitos sinkhole|BOLD:ACX6091
Mysidae|BACZP040-15|Bacalar Lake, Cocalitos Sinkhole|BOLD:ACX6091
Mysidae|BACZP038-15|Bacalar Lake, Cocalitos Sinkhole|BOLD:ACX6091
Mysidae|BACZP037-15|Bacalar Lake, Cocalitos Sinkhole|BOLD:ACX6091
Mysidae|BACZP044-15|Bacalar Lake, Cocalitos Sinkhole|BOLD:ACX6091
Mysida|BACZP645-16|Bacalar Lake, Cocalitos Sinkhole|BOLD:ACX6091
Mysidae|BACZP872-16|Bacalar Lake, Cocalitos Sinkhole|BOLD:ACX6091
Mysidae|BACZP875-16|Bacalar Lake, Cocalitos Sinkhole|BOLD:ACX6091
Mysidae|BACZP910-16|Bacalar Lake, Cocalitos Sinkhole|BOLD:ACX6091
Mysidae|BACZP911-16|Bacalar Lake, Cocalitos Sinkhole|BOLD:ACX6091
Sesarma|BACZP2210-16|Bacalar Lake, Cocalitos Sinkhole|BOLD:ACX4604
Sesarma|BACZP2211-16|Bacalar Lake, Cocalitos Sinkhole|BOLD:ACX4604
Sesarma|BACZP2212-16|Bacalar Lake, Cocalitos Sinkhole|BOLD:ACX4604
Sesarma|BACZP2213-16|Bacalar Lake, Cocalitos Sinkhole|BOLD:ACX4604
Sesarma|BACZP2255-16|Bacalar Lake, Cocalitos Sinkhole|BOLD:ACX4604
Sesarma|BACZP904-16|Bacalar Lake, Cocalitos Sinkhole|BOLD:ACX4604
Sesarma|BACZP871-16|Bacalar Lake, Cocalitos Sinkhole|BOLD:ACX4604
Sesarma|BACZP870-16|Bacalar Lake, Cocalitos Sinkhole|BOLD:ACX4604
Sesarma|BACZP869-16|Bacalar Lake, Cocalitos Sinkhole|BOLD:ACX4604
Sesarma|BACZP868-16|Bacalar Lake, Cocalitos Sinkhole|BOLD:ACX4604
Sesarma|BACZP644-16|Bacalar Lake, Cocalitos Sinkhole|BOLD:ACX4604
Sesarma|BACZP643-16|Bacalar Lake, Cocalitos Sinkhole|BOLD:ACX4604
Sesarma|BACZP642-16|Bacalar Lake, Cocalitos Sinkhole|BOLD:ACX4604
Sesarma|BACZP071-15|Bacalar Lake, Cocalitos sinkhole, light trap 1|BOLD:ACX4604
Sesarma|BACZP070-15|Bacalar Lake, Cocalitos sinkhole, light trap 1|BOLD:ACX4604
Sesarma|BACZP033-15|Bacalar Lake, Cocalitos Sinkhole|BOLD:ACX4604
Sesarma|BACZP031-15|Bacalar Lake, Cocalitos Sinkhole|BOLD:ACX4604
Cypridopsis|BACZP076-15|Bacalar Lake, Normal sinkhole|
Sesarmidae|BACZP905-16|Bacalar Lake, Cocalitos Sinkhole|BOLD:ACX4604
Sesarma|BACZP906-16|Bacalar Lake, Cocalitos Sinkhole|BOLD:ACX4604
Sesarma|BACZP907-16|Bacalar Lake, Cocalitos Sinkhole|BOLD:ACX4604
Sesarma|BACZP2331-16|Bacalar Lake, Cocalitos Sinkhole|BOLD:ACX4604
Sesarma|BACZP2332-16|Bacalar Lake, Cocalitos Sinkhole|BOLD:ACX4604
Sesarma|BACZP2340-16|Bacalar Lake, Cocalitos Sinkhole|BOLD:ACX4604
Sesarma|BACZP2347-16|Bacalar Lake, Cocalitos Sinkhole|BOLD:ACX4604
Sesarma|BACZP2372-16|Bacalar Lake, Cocalitos Sinkhole|BOLD:ACX4604
Sesarma|BACZP2381-16|Bacalar Lake, Cocalitos Sinkhole|BOLD:ACX4604
Sesarma|BACZP2214-16|Bacalar Lake, Cocalitos Sinkhole|BOLD:ACX4604
Sesarma|BACZP2256-16|Bacalar Lake, Cocalitos Sinkhole|BOLD:ACX4604
Alona|BACZP368-15|Guelph Lake|
Amphipoda|BACZP035-15|Bacalar Lake, Cocalitos Sinkhole|BOLD:ACX5005
Amphipoda|BACZP081-15|Xulha Lake|BOLD:ACX5005
Amphipoda|BACZP473-16|Bacalar Lake, Cocalitos Sinkhole, plankton net|BOLD:ACX5005
Amphipoda|BACZP046-15|Bacalar Lake, Cocalitos grass zone|BOLD:ACX4754
Amphipoda|BACZP050-15|Bacalar Lake, Cocalitos grass zone|
Amphipoda|BACZP141-15|Bacalar Lake, Cocalitos grass zone|BOLD:ACX4754
Amphipoda|BACZP148-15|Bacalar Lake, Cocalitos grass zone|BOLD:ACX4754
Amphipoda|BACZP647-16|Bacalar Lake, Cocalitos Sinkhole|BOLD:ACX4754
Amphipoda|BACZP406-16|Chipila|BOLD:ACZ1982
Amphipoda|BACZP417-16|Chipila|BOLD:ACZ1982
Amphipoda|BACZP385-16|Atlangatepec|BOLD:ACZ2489
Amphipoda|BACZP396-16|Atlangatepec|BOLD:ACZ2489
Hyalella|BACZP1305-16|Guelph lake swamp|BOLD:AAA1704
Hyalella|BACZP291-15|Pond in the other side Guelph Lake|BOLD:AAA1704
Hyalella|BACZP323-15|Pond in the other side Guelph Lake|BOLD:AAA1704
Hyalella|BACZP1340-16|Guelph Lake Swamp|BOLD:AAA1707
Hyalella|BACZP1478-16|Crawford lake|BOLD:AAA1707
Hyalella|BACZP1307-16|Guelph lake swamp|BOLD:AAA1707

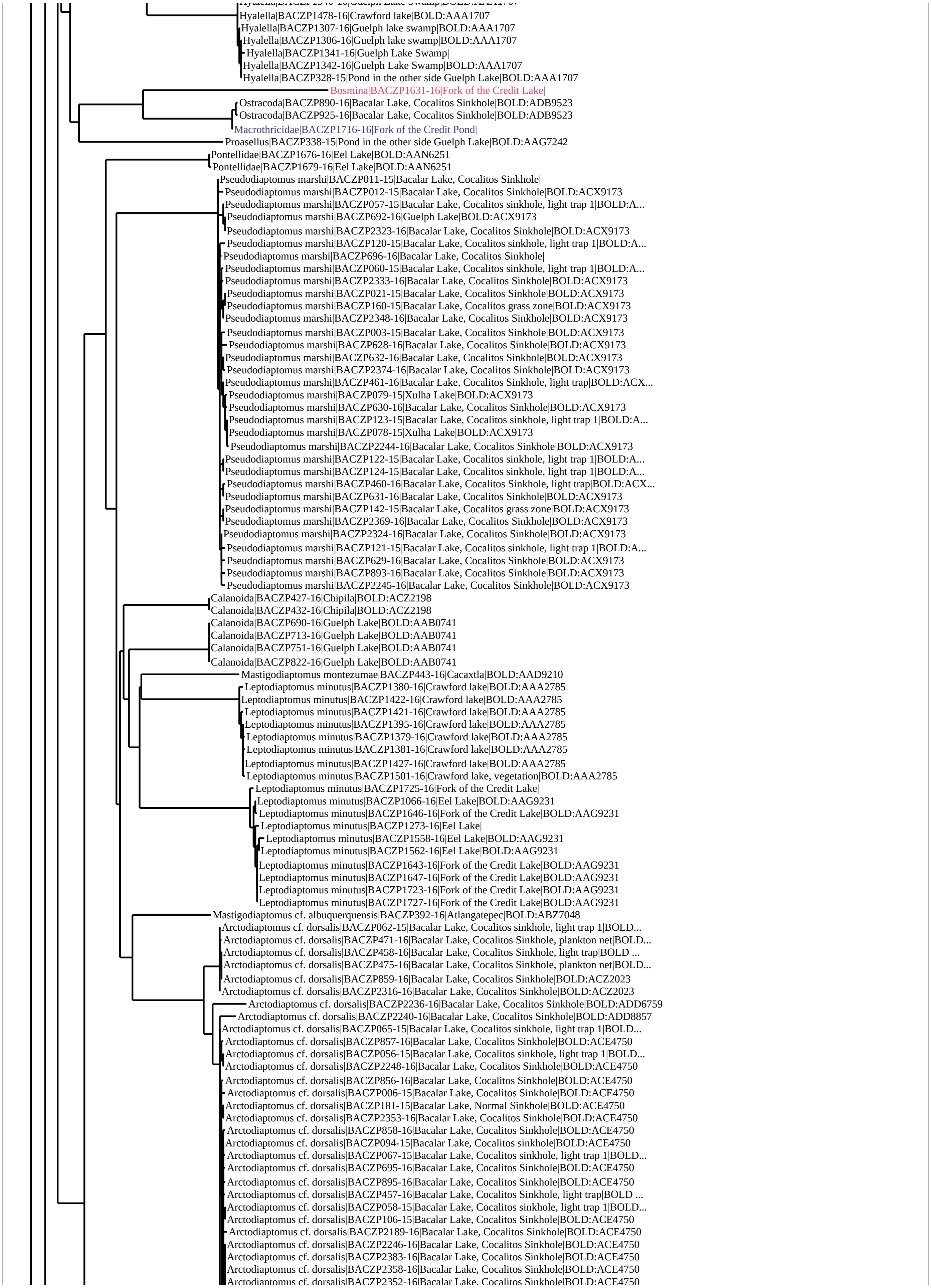
Hyalella|BACZP1478-16|Crawford lake|BOLD:AAA1707
Hyalella|BACZP1307-16|Guelph lake swamp|BOLD:AAA1707
Hyalella|BACZP1306-16|Guelph lake swamp|BOLD:AAA1707
Hyalella|BACZP1341-16|Guelph Lake Swamp|
Hyalella|BACZP1342-16|Guelph Lake Swamp|BOLD:AAA1707
Hyalella|BACZP328-15|Pond in the other side Guelph Lake|BOLD:AAA1707
Bosmina|BACZP1631-16|Fork of the Credit Lake|
Ostracoda|BACZP890-16|Bacalar Lake, Cocalitos Sinkhole|BOLD:ADB9523
Ostracoda|BACZP925-16|Bacalar Lake, Cocalitos Sinkhole|BOLD:ADB9523
Macrothricidae|BACZP1716-16|Fork of the Credit Pond|
Proasellus|BACZP338-15|Pond in the other side Guelph Lake|BOLD:AAG7242
Pontellidae|BACZP1676-16|Eel Lake|BOLD:AAN6251
Pontellidae|BACZP1679-16|Eel Lake|BOLD:AAN6251
Pseudodiaptomus marshi|BACZP011-15|Bacalar Lake, Cocalitos Sinkhole|
Pseudodiaptomus marshi|BACZP012-15|Bacalar Lake, Cocalitos Sinkhole|BOLD:ACX9173
Pseudodiaptomus marshi|BACZP057-15|Bacalar Lake, Cocalitos sinkhole, light trap 1|BOLD:A...
Pseudodiaptomus marshi|BACZP692-16|Guelph Lake|BOLD:ACX9173
Pseudodiaptomus marshi|BACZP2323-16|Bacalar Lake, Cocalitos Sinkhole|BOLD:ACX9173
Pseudodiaptomus marshi|BACZP120-15|Bacalar Lake, Cocalitos sinkhole, light trap 1|BOLD:A...
Pseudodiaptomus marshi|BACZP696-16|Bacalar Lake, Cocalitos Sinkhole|
Pseudodiaptomus marshi|BACZP060-15|Bacalar Lake, Cocalitos sinkhole, light trap 1|BOLD:A...
Pseudodiaptomus marshi|BACZP2333-16|Bacalar Lake, Cocalitos Sinkhole|BOLD:ACX9173
Pseudodiaptomus marshi|BACZP021-15|Bacalar Lake, Cocalitos Sinkhole|BOLD:ACX9173
Pseudodiaptomus marshi|BACZP160-15|Bacalar Lake, Cocalitos grass zone|BOLD:ACX9173
Pseudodiaptomus marshi|BACZP2348-16|Bacalar Lake, Cocalitos Sinkhole|BOLD:ACX9173
Pseudodiaptomus marshi|BACZP003-15|Bacalar Lake, Cocalitos Sinkhole|BOLD:ACX9173
Pseudodiaptomus marshi|BACZP628-16|Bacalar Lake, Cocalitos Sinkhole|BOLD:ACX9173
Pseudodiaptomus marshi|BACZP632-16|Bacalar Lake, Cocalitos Sinkhole|BOLD:ACX9173
Pseudodiaptomus marshi|BACZP2374-16|Bacalar Lake, Cocalitos Sinkhole|BOLD:ACX9173
Pseudodiaptomus marshi|BACZP461-16|Bacalar Lake, Cocalitos Sinkhole, light trap|BOLD:ACX...
Pseudodiaptomus marshi|BACZP079-15|Xulha Lake|BOLD:ACX9173
Pseudodiaptomus marshi|BACZP630-16|Bacalar Lake, Cocalitos Sinkhole|BOLD:ACX9173
Pseudodiaptomus marshi|BACZP123-15|Bacalar Lake, Cocalitos sinkhole, light trap 1|BOLD:A...
Pseudodiaptomus marshi|BACZP078-15|Xulha Lake|BOLD:ACX9173
Pseudodiaptomus marshi|BACZP2244-16|Bacalar Lake, Cocalitos Sinkhole|BOLD:ACX9173
Pseudodiaptomus marshi|BACZP122-15|Bacalar Lake, Cocalitos sinkhole, light trap 1|BOLD:A...
Pseudodiaptomus marshi|BACZP124-15|Bacalar Lake, Cocalitos sinkhole, light trap 1|BOLD:A...
Pseudodiaptomus marshi|BACZP460-16|Bacalar Lake, Cocalitos Sinkhole, light trap|BOLD:ACX...
Pseudodiaptomus marshi|BACZP631-16|Bacalar Lake, Cocalitos Sinkhole|BOLD:ACX9173
Pseudodiaptomus marshi|BACZP142-15|Bacalar Lake, Cocalitos grass zone|BOLD:ACX9173
Pseudodiaptomus marshi|BACZP2369-16|Bacalar Lake, Cocalitos Sinkhole|BOLD:ACX9173
Pseudodiaptomus marshi|BACZP2324-16|Bacalar Lake, Cocalitos Sinkhole|BOLD:ACX9173
Pseudodiaptomus marshi|BACZP121-15|Bacalar Lake, Cocalitos sinkhole, light trap 1|BOLD:A...
Pseudodiaptomus marshi|BACZP629-16|Bacalar Lake, Cocalitos Sinkhole|BOLD:ACX9173
Pseudodiaptomus marshi|BACZP893-16|Bacalar Lake, Cocalitos Sinkhole|BOLD:ACX9173
Pseudodiaptomus marshi|BACZP2245-16|Bacalar Lake, Cocalitos Sinkhole|BOLD:ACX9173
Calanoida|BACZP427-16|Chipila|BOLD:ACZ2198
Calanoida|BACZP432-16|Chipila|BOLD:ACZ2198
Calanoida|BACZP690-16|Guelph Lake|BOLD:AAB0741
Calanoida|BACZP713-16|Guelph Lake|BOLD:AAB0741
Calanoida|BACZP751-16|Guelph Lake|BOLD:AAB0741
Calanoida|BACZP822-16|Guelph Lake|BOLD:AAB0741
Mastigodiaptomus montezumae|BACZP443-16|Cacaxtla|BOLD:AAD9210
Leptodiaptomus minutus|BACZP1380-16|Crawford lake|BOLD:AAA2785
Leptodiaptomus minutus|BACZP1422-16|Crawford lake|BOLD:AAA2785
Leptodiaptomus minutus|BACZP1421-16|Crawford lake|BOLD:AAA2785
Leptodiaptomus minutus|BACZP1395-16|Crawford lake|BOLD:AAA2785
Leptodiaptomus minutus|BACZP1379-16|Crawford lake|BOLD:AAA2785
Leptodiaptomus minutus|BACZP1381-16|Crawford lake|BOLD:AAA2785
Leptodiaptomus minutus|BACZP1427-16|Crawford lake|BOLD:AAA2785
Leptodiaptomus minutus|BACZP1501-16|Crawford lake, vegetation|BOLD:AAA2785
Leptodiaptomus minutus|BACZP1725-16|Fork of the Credit Lake|
Leptodiaptomus minutus|BACZP1066-16|Eel Lake|BOLD:AAG9231
Leptodiaptomus minutus|BACZP1646-16|Fork of the Credit Lake|BOLD:AAG9231
Leptodiaptomus minutus|BACZP1273-16|Eel Lake|
Leptodiaptomus minutus|BACZP1558-16|Eel Lake|BOLD:AAG9231
Leptodiaptomus minutus|BACZP1562-16|Eel Lake|BOLD:AAG9231
Leptodiaptomus minutus|BACZP1643-16|Fork of the Credit Lake|BOLD:AAG9231
Leptodiaptomus minutus|BACZP1647-16|Fork of the Credit Lake|BOLD:AAG9231
Leptodiaptomus minutus|BACZP1723-16|Fork of the Credit Lake|BOLD:AAG9231
Leptodiaptomus minutus|BACZP1727-16|Fork of the Credit Lake|BOLD:AAG9231
Mastigodiaptomus cf. albuquerquensis|BACZP392-16|Atlangatepec|BOLD:ABZ7048
Arctodiaptomus cf. dorsalis|BACZP062-15|Bacalar Lake, Cocalitos sinkhole, light trap 1|BOLD...
Arctodiaptomus cf. dorsalis|BACZP471-16|Bacalar Lake, Cocalitos Sinkhole, plankton net|BOLD...
Arctodiaptomus cf. dorsalis|BACZP458-16|Bacalar Lake, Cocalitos Sinkhole, light trap|BOLD ...
Arctodiaptomus cf. dorsalis|BACZP475-16|Bacalar Lake, Cocalitos Sinkhole, plankton net|BOLD...
Arctodiaptomus cf. dorsalis|BACZP859-16|Bacalar Lake, Cocalitos Sinkhole|BOLD:ACZ2023
Arctodiaptomus cf. dorsalis|BACZP2316-16|Bacalar Lake, Cocalitos Sinkhole|BOLD:ACZ2023
Arctodiaptomus cf. dorsalis|BACZP2236-16|Bacalar Lake, Cocalitos Sinkhole|BOLD:ADD6759
Arctodiaptomus cf. dorsalis|BACZP2240-16|Bacalar Lake, Cocalitos Sinkhole|BOLD:ADD8857
Arctodiaptomus cf. dorsalis|BACZP065-15|Bacalar Lake, Cocalitos sinkhole, light trap 1|BOLD...
Arctodiaptomus cf. dorsalis|BACZP857-16|Bacalar Lake, Cocalitos Sinkhole|BOLD:ACE4750
Arctodiaptomus cf. dorsalis|BACZP056-15|Bacalar Lake, Cocalitos sinkhole, light trap 1|BOLD...
Arctodiaptomus cf. dorsalis|BACZP2248-16|Bacalar Lake, Cocalitos Sinkhole|BOLD:ACE4750
Arctodiaptomus cf. dorsalis|BACZP856-16|Bacalar Lake, Cocalitos Sinkhole|BOLD:ACE4750
Arctodiaptomus cf. dorsalis|BACZP006-15|Bacalar Lake, Cocalitos Sinkhole|BOLD:ACE4750
Arctodiaptomus cf. dorsalis|BACZP181-15|Bacalar Lake, Normal Sinkhole|BOLD:ACE4750
Arctodiaptomus cf. dorsalis|BACZP2353-16|Bacalar Lake, Cocalitos Sinkhole|BOLD:ACE4750
Arctodiaptomus cf. dorsalis|BACZP858-16|Bacalar Lake, Cocalitos Sinkhole|BOLD:ACE4750
Arctodiaptomus cf. dorsalis|BACZP094-15|Bacalar Lake, Cocalitos sinkhole|BOLD:ACE4750
Arctodiaptomus cf. dorsalis|BACZP067-15|Bacalar Lake, Cocalitos sinkhole, light trap 1|BOLD...
Arctodiaptomus cf. dorsalis|BACZP695-16|Bacalar Lake, Cocalitos Sinkhole|BOLD:ACE4750
Arctodiaptomus cf. dorsalis|BACZP895-16|Bacalar Lake, Cocalitos Sinkhole|BOLD:ACE4750
Arctodiaptomus cf. dorsalis|BACZP457-16|Bacalar Lake, Cocalitos Sinkhole, light trap|BOLD ...
Arctodiaptomus cf. dorsalis|BACZP058-15|Bacalar Lake, Cocalitos sinkhole, light trap 1|BOLD...
Arctodiaptomus cf. dorsalis|BACZP106-15|Bacalar Lake, Cocalitos Sinkhole|BOLD:ACE4750
Arctodiaptomus cf. dorsalis|BACZP2189-16|Bacalar Lake, Cocalitos Sinkhole|BOLD:ACE4750
Arctodiaptomus cf. dorsalis|BACZP2246-16|Bacalar Lake, Cocalitos Sinkhole|BOLD:ACE4750
Arctodiaptomus cf. dorsalis|BACZP2383-16|Bacalar Lake, Cocalitos Sinkhole|BOLD:ACE4750
Arctodiaptomus cf. dorsalis|BACZP2358-16|Bacalar Lake, Cocalitos Sinkhole|BOLD:ACE4750
Arctodiaptomus cf. dorsalis|BACZP2352-16|Bacalar Lake, Cocalitos Sinkhole|BOLD:ACE4750

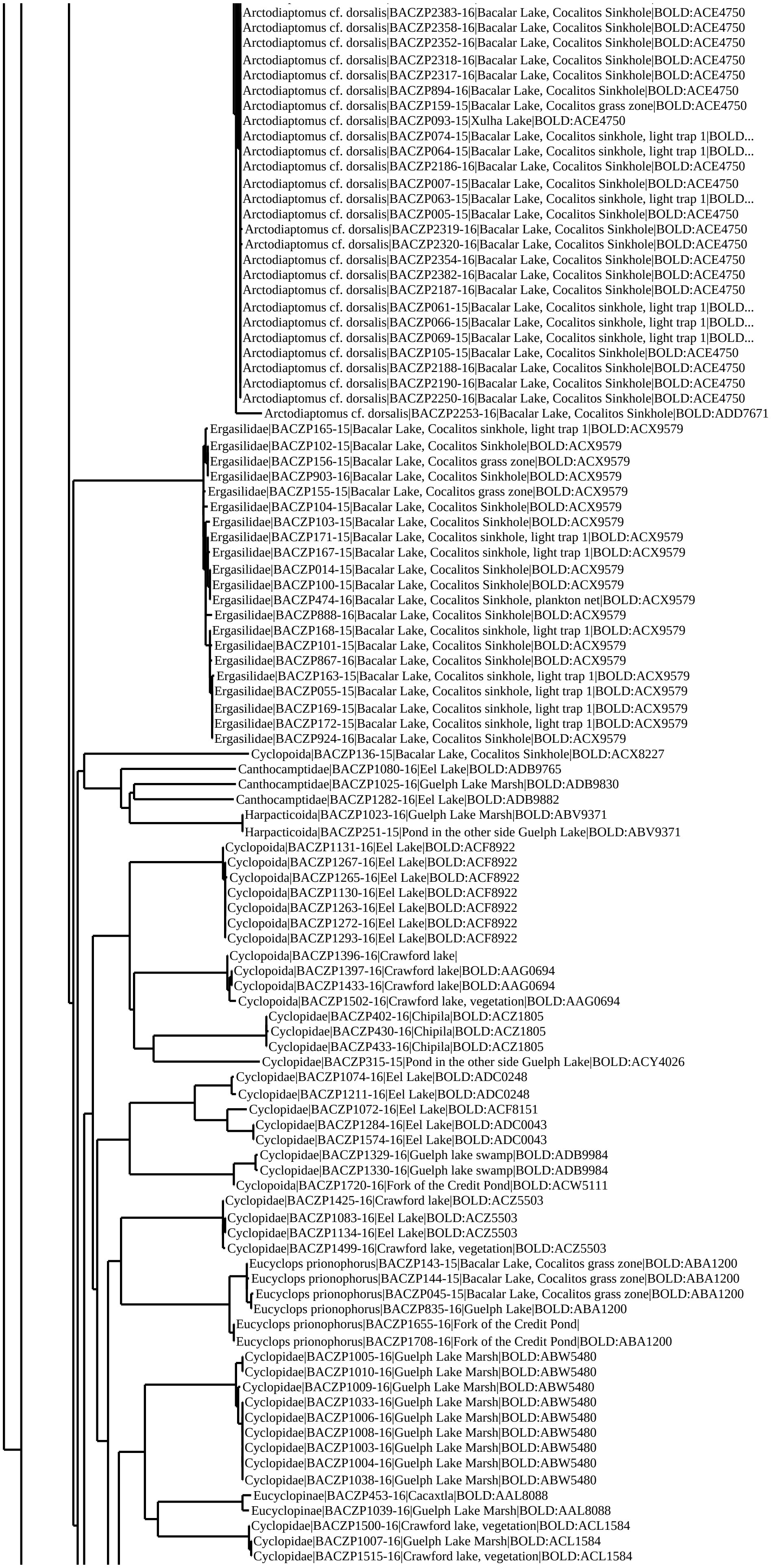
Arctodiaptomus cf. dorsalis|BACZP2383-16|Bacalar Lake, Cocalitos Sinkhole|BOLD:ACE4750
Arctodiaptomus cf. dorsalis|BACZP2358-16|Bacalar Lake, Cocalitos Sinkhole|BOLD:ACE4750
Arctodiaptomus cf. dorsalis|BACZP2352-16|Bacalar Lake, Cocalitos Sinkhole|BOLD:ACE4750
Arctodiaptomus cf. dorsalis|BACZP2318-16|Bacalar Lake, Cocalitos Sinkhole|BOLD:ACE4750
Arctodiaptomus cf. dorsalis|BACZP2317-16|Bacalar Lake, Cocalitos Sinkhole|BOLD:ACE4750
Arctodiaptomus cf. dorsalis|BACZP894-16|Bacalar Lake, Cocalitos Sinkhole|BOLD:ACE4750
Arctodiaptomus cf. dorsalis|BACZP159-15|Bacalar Lake, Cocalitos grass zone|BOLD:ACE4750
Arctodiaptomus cf. dorsalis|BACZP093-15|Xulha Lake|BOLD:ACE4750
Arctodiaptomus cf. dorsalis|BACZP074-15|Bacalar Lake, Cocalitos sinkhole, light trap 1|BOLD...
Arctodiaptomus cf. dorsalis|BACZP064-15|Bacalar Lake, Cocalitos sinkhole, light trap 1|BOLD...
Arctodiaptomus cf. dorsalis|BACZP2186-16|Bacalar Lake, Cocalitos Sinkhole|BOLD:ACE4750
Arctodiaptomus cf. dorsalis|BACZP007-15|Bacalar Lake, Cocalitos Sinkhole|BOLD:ACE4750
Arctodiaptomus cf. dorsalis|BACZP063-15|Bacalar Lake, Cocalitos sinkhole, light trap 1|BOLD...
Arctodiaptomus cf. dorsalis|BACZP005-15|Bacalar Lake, Cocalitos Sinkhole|BOLD:ACE4750
Arctodiaptomus cf. dorsalis|BACZP2319-16|Bacalar Lake, Cocalitos Sinkhole|BOLD:ACE4750
Arctodiaptomus cf. dorsalis|BACZP2320-16|Bacalar Lake, Cocalitos Sinkhole|BOLD:ACE4750
Arctodiaptomus cf. dorsalis|BACZP2354-16|Bacalar Lake, Cocalitos Sinkhole|BOLD:ACE4750
Arctodiaptomus cf. dorsalis|BACZP2382-16|Bacalar Lake, Cocalitos Sinkhole|BOLD:ACE4750
Arctodiaptomus cf. dorsalis|BACZP2187-16|Bacalar Lake, Cocalitos Sinkhole|BOLD:ACE4750
Arctodiaptomus cf. dorsalis|BACZP061-15|Bacalar Lake, Cocalitos sinkhole, light trap 1|BOLD...
Arctodiaptomus cf. dorsalis|BACZP066-15|Bacalar Lake, Cocalitos sinkhole, light trap 1|BOLD...
Arctodiaptomus cf. dorsalis|BACZP069-15|Bacalar Lake, Cocalitos sinkhole, light trap 1|BOLD...
Arctodiaptomus cf. dorsalis|BACZP105-15|Bacalar Lake, Cocalitos Sinkhole|BOLD:ACE4750
Arctodiaptomus cf. dorsalis|BACZP2188-16|Bacalar Lake, Cocalitos Sinkhole|BOLD:ACE4750
Arctodiaptomus cf. dorsalis|BACZP2190-16|Bacalar Lake, Cocalitos Sinkhole|BOLD:ACE4750
Arctodiaptomus cf. dorsalis|BACZP2250-16|Bacalar Lake, Cocalitos Sinkhole|BOLD:ACE4750
Arctodiaptomus cf. dorsalis|BACZP2253-16|Bacalar Lake, Cocalitos Sinkhole|BOLD:ADD7671
Ergasilidae|BACZP165-15|Bacalar Lake, Cocalitos sinkhole, light trap 1|BOLD:ACX9579
Ergasilidae|BACZP102-15|Bacalar Lake, Cocalitos Sinkhole|BOLD:ACX9579
Ergasilidae|BACZP156-15|Bacalar Lake, Cocalitos grass zone|BOLD:ACX9579
Ergasilidae|BACZP903-16|Bacalar Lake, Cocalitos Sinkhole|BOLD:ACX9579
Ergasilidae|BACZP155-15|Bacalar Lake, Cocalitos grass zone|BOLD:ACX9579
Ergasilidae|BACZP104-15|Bacalar Lake, Cocalitos Sinkhole|BOLD:ACX9579
Ergasilidae|BACZP103-15|Bacalar Lake, Cocalitos Sinkhole|BOLD:ACX9579
Ergasilidae|BACZP171-15|Bacalar Lake, Cocalitos sinkhole, light trap 1|BOLD:ACX9579
Ergasilidae|BACZP167-15|Bacalar Lake, Cocalitos sinkhole, light trap 1|BOLD:ACX9579
Ergasilidae|BACZP014-15|Bacalar Lake, Cocalitos Sinkhole|BOLD:ACX9579
Ergasilidae|BACZP100-15|Bacalar Lake, Cocalitos Sinkhole|BOLD:ACX9579
Ergasilidae|BACZP474-16|Bacalar Lake, Cocalitos Sinkhole, plankton net|BOLD:ACX9579
Ergasilidae|BACZP888-16|Bacalar Lake, Cocalitos Sinkhole|BOLD:ACX9579
Ergasilidae|BACZP168-15|Bacalar Lake, Cocalitos sinkhole, light trap 1|BOLD:ACX9579
Ergasilidae|BACZP101-15|Bacalar Lake, Cocalitos Sinkhole|BOLD:ACX9579
Ergasilidae|BACZP867-16|Bacalar Lake, Cocalitos Sinkhole|BOLD:ACX9579
Ergasilidae|BACZP163-15|Bacalar Lake, Cocalitos sinkhole, light trap 1|BOLD:ACX9579
Ergasilidae|BACZP055-15|Bacalar Lake, Cocalitos sinkhole, light trap 1|BOLD:ACX9579
Ergasilidae|BACZP169-15|Bacalar Lake, Cocalitos sinkhole, light trap 1|BOLD:ACX9579
Ergasilidae|BACZP172-15|Bacalar Lake, Cocalitos sinkhole, light trap 1|BOLD:ACX9579
Ergasilidae|BACZP924-16|Bacalar Lake, Cocalitos Sinkhole|BOLD:ACX9579
Cyclopoida|BACZP136-15|Bacalar Lake, Cocalitos Sinkhole|BOLD:ACX8227
Canthocamptidae|BACZP1080-16|Eel Lake|BOLD:ADB9765
Canthocamptidae|BACZP1025-16|Guelph Lake Marsh|BOLD:ADB9830
Canthocamptidae|BACZP1282-16|Eel Lake|BOLD:ADB9882
Harpacticoida|BACZP1023-16|Guelph Lake Marsh|BOLD:ABV9371
Harpacticoida|BACZP251-15|Pond in the other side Guelph Lake|BOLD:ABV9371
Cyclopoida|BACZP1131-16|Eel Lake|BOLD:ACF8922
Cyclopoida|BACZP1267-16|Eel Lake|BOLD:ACF8922
Cyclopoida|BACZP1265-16|Eel Lake|BOLD:ACF8922
Cyclopoida|BACZP1130-16|Eel Lake|BOLD:ACF8922
Cyclopoida|BACZP1263-16|Eel Lake|BOLD:ACF8922
Cyclopoida|BACZP1272-16|Eel Lake|BOLD:ACF8922
Cyclopoida|BACZP1293-16|Eel Lake|BOLD:ACF8922
Cyclopoida|BACZP1396-16|Crawford lake|
Cyclopoida|BACZP1397-16|Crawford lake|BOLD:AAG0694
Cyclopoida|BACZP1433-16|Crawford lake|BOLD:AAG0694
Cyclopoida|BACZP1502-16|Crawford lake, vegetation|BOLD:AAG0694
Cyclopidae|BACZP402-16|Chipila|BOLD:ACZ1805
Cyclopidae|BACZP430-16|Chipila|BOLD:ACZ1805
Cyclopidae|BACZP433-16|Chipila|BOLD:ACZ1805
Cyclopidae|BACZP315-15|Pond in the other side Guelph Lake|BOLD:ACY4026
Cyclopidae|BACZP1074-16|Eel Lake|BOLD:ADC0248
Cyclopidae|BACZP1211-16|Eel Lake|BOLD:ADC0248
Cyclopidae|BACZP1072-16|Eel Lake|BOLD:ACF8151
Cyclopidae|BACZP1284-16|Eel Lake|BOLD:ADC0043
Cyclopidae|BACZP1574-16|Eel Lake|BOLD:ADC0043
Cyclopidae|BACZP1329-16|Guelph lake swamp|BOLD:ADB9984
Cyclopidae|BACZP1330-16|Guelph lake swamp|BOLD:ADB9984
Cyclopoida|BACZP1720-16|Fork of the Credit Pond|BOLD:ACW5111
Cyclopidae|BACZP1425-16|Crawford lake|BOLD:ACZ5503
Cyclopidae|BACZP1083-16|Eel Lake|BOLD:ACZ5503
Cyclopidae|BACZP1134-16|Eel Lake|BOLD:ACZ5503
Cyclopidae|BACZP1499-16|Crawford lake, vegetation|BOLD:ACZ5503
Eucyclops prionophorus|BACZP143-15|Bacalar Lake, Cocalitos grass zone|BOLD:ABA1200
Eucyclops prionophorus|BACZP144-15|Bacalar Lake, Cocalitos grass zone|BOLD:ABA1200
Eucyclops prionophorus|BACZP045-15|Bacalar Lake, Cocalitos grass zone|BOLD:ABA1200
Eucyclops prionophorus|BACZP835-16|Guelph Lake|BOLD:ABA1200
Eucyclops prionophorus|BACZP1655-16|Fork of the Credit Pond|
Eucyclops prionophorus|BACZP1708-16|Fork of the Credit Pond|BOLD:ABA1200
Cyclopidae|BACZP1005-16|Guelph Lake Marsh|BOLD:ABW5480
Cyclopidae|BACZP1010-16|Guelph Lake Marsh|BOLD:ABW5480
Cyclopidae|BACZP1009-16|Guelph Lake Marsh|BOLD:ABW5480
Cyclopidae|BACZP1033-16|Guelph Lake Marsh|BOLD:ABW5480
Cyclopidae|BACZP1006-16|Guelph Lake Marsh|BOLD:ABW5480
Cyclopidae|BACZP1008-16|Guelph Lake Marsh|BOLD:ABW5480
Cyclopidae|BACZP1003-16|Guelph Lake Marsh|BOLD:ABW5480
Cyclopidae|BACZP1004-16|Guelph Lake Marsh|BOLD:ABW5480
Cyclopidae|BACZP1038-16|Guelph Lake Marsh|BOLD:ABW5480
Eucyclopinae|BACZP453-16|Cacaxtla|BOLD:AAL8088
Eucyclopinae|BACZP1039-16|Guelph Lake Marsh|BOLD:AAL8088
Cyclopidae|BACZP1500-16|Crawford lake, vegetation|BOLD:ACL1584
Cyclopidae|BACZP1007-16|Guelph Lake Marsh|BOLD:ACL1584
Cyclopidae|BACZP1515-16|Crawford lake, vegetation|BOLD:ACL1584

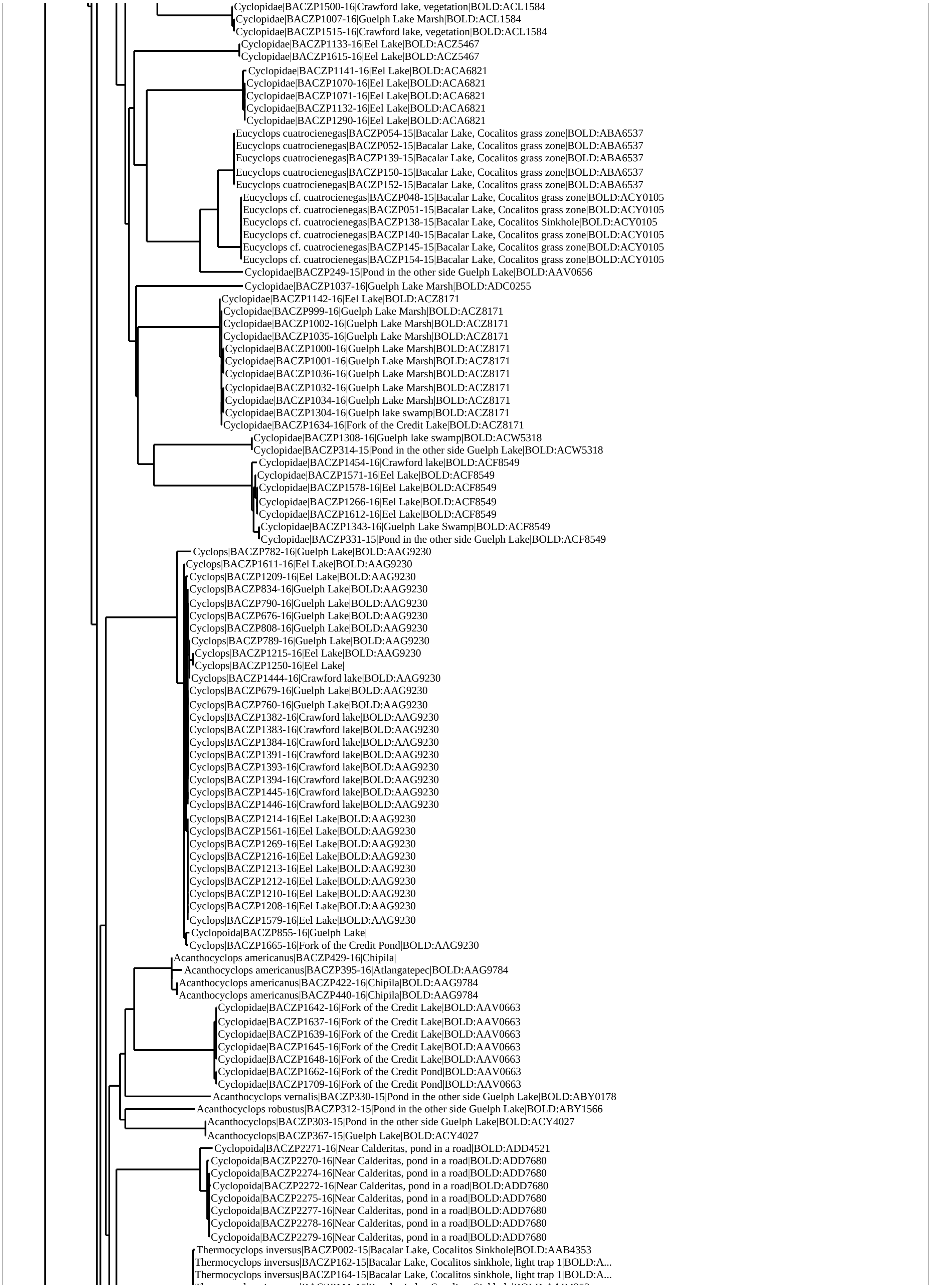
Cyclopidae|BACZP1500-16|Crawford lake, vegetation|BOLD:ACL1584
Cyclopidae|BACZP1007-16|Guelph Lake Marsh|BOLD:ACL1584
Cyclopidae|BACZP1515-16|Crawford lake, vegetation|BOLD:ACL1584
Cyclopidae|BACZP1133-16|Eel Lake|BOLD:ACZ5467
Cyclopidae|BACZP1615-16|Eel Lake|BOLD:ACZ5467
Cyclopidae|BACZP1141-16|Eel Lake|BOLD:ACA6821
Cyclopidae|BACZP1070-16|Eel Lake|BOLD:ACA6821
Cyclopidae|BACZP1071-16|Eel Lake|BOLD:ACA6821
Cyclopidae|BACZP1132-16|Eel Lake|BOLD:ACA6821
Cyclopidae|BACZP1290-16|Eel Lake|BOLD:ACA6821
Eucyclops cuatrocienegas|BACZP054-15|Bacalar Lake, Cocalitos grass zone|BOLD:ABA6537
Eucyclops cuatrocienegas|BACZP052-15|Bacalar Lake, Cocalitos grass zone|BOLD:ABA6537
Eucyclops cuatrocienegas|BACZP139-15|Bacalar Lake, Cocalitos grass zone|BOLD:ABA6537
Eucyclops cuatrocienegas|BACZP150-15|Bacalar Lake, Cocalitos grass zone|BOLD:ABA6537
Eucyclops cuatrocienegas|BACZP152-15|Bacalar Lake, Cocalitos grass zone|BOLD:ABA6537
Eucyclops cf. cuatrocienegas|BACZP048-15|Bacalar Lake, Cocalitos grass zone|BOLD:ACY0105
Eucyclops cf. cuatrocienegas|BACZP051-15|Bacalar Lake, Cocalitos grass zone|BOLD:ACY0105
Eucyclops cf. cuatrocienegas|BACZP138-15|Bacalar Lake, Cocalitos Sinkhole|BOLD:ACY0105
Eucyclops cf. cuatrocienegas|BACZP140-15|Bacalar Lake, Cocalitos grass zone|BOLD:ACY0105
Eucyclops cf. cuatrocienegas|BACZP145-15|Bacalar Lake, Cocalitos grass zone|BOLD:ACY0105
Eucyclops cf. cuatrocienegas|BACZP154-15|Bacalar Lake, Cocalitos grass zone|BOLD:ACY0105
Cyclopidae|BACZP249-15|Pond in the other side Guelph Lake|BOLD:AAV0656
Cyclopidae|BACZP1037-16|Guelph Lake Marsh|BOLD:ADC0255
Cyclopidae|BACZP1142-16|Eel Lake|BOLD:ACZ8171
Cyclopidae|BACZP999-16|Guelph Lake Marsh|BOLD:ACZ8171
Cyclopidae|BACZP1002-16|Guelph Lake Marsh|BOLD:ACZ8171
Cyclopidae|BACZP1035-16|Guelph Lake Marsh|BOLD:ACZ8171
Cyclopidae|BACZP1000-16|Guelph Lake Marsh|BOLD:ACZ8171
Cyclopidae|BACZP1001-16|Guelph Lake Marsh|BOLD:ACZ8171
Cyclopidae|BACZP1036-16|Guelph Lake Marsh|BOLD:ACZ8171
Cyclopidae|BACZP1032-16|Guelph Lake Marsh|BOLD:ACZ8171
Cyclopidae|BACZP1034-16|Guelph Lake Marsh|BOLD:ACZ8171
Cyclopidae|BACZP1304-16|Guelph lake swamp|BOLD:ACZ8171
Cyclopidae|BACZP1634-16|Fork of the Credit Lake|BOLD:ACZ8171
Cyclopidae|BACZP1308-16|Guelph lake swamp|BOLD:ACW5318
Cyclopidae|BACZP314-15|Pond in the other side Guelph Lake|BOLD:ACW5318
Cyclopidae|BACZP1454-16|Crawford lake|BOLD:ACF8549
Cyclopidae|BACZP1571-16|Eel Lake|BOLD:ACF8549
Cyclopidae|BACZP1578-16|Eel Lake|BOLD:ACF8549
Cyclopidae|BACZP1266-16|Eel Lake|BOLD:ACF8549
Cyclopidae|BACZP1612-16|Eel Lake|BOLD:ACF8549
Cyclopidae|BACZP1343-16|Guelph Lake Swamp|BOLD:ACF8549
Cyclopidae|BACZP331-15|Pond in the other side Guelph Lake|BOLD:ACF8549
Cyclops|BACZP782-16|Guelph Lake|BOLD:AAG9230
Cyclops|BACZP1611-16|Eel Lake|BOLD:AAG9230
Cyclops|BACZP1209-16|Eel Lake|BOLD:AAG9230
Cyclops|BACZP834-16|Guelph Lake|BOLD:AAG9230
Cyclops|BACZP790-16|Guelph Lake|BOLD:AAG9230
Cyclops|BACZP676-16|Guelph Lake|BOLD:AAG9230
Cyclops|BACZP808-16|Guelph Lake|BOLD:AAG9230
Cyclops|BACZP789-16|Guelph Lake|BOLD:AAG9230
Cyclops|BACZP1215-16|Eel Lake|BOLD:AAG9230
Cyclops|BACZP1250-16|Eel Lake|
Cyclops|BACZP1444-16|Crawford lake|BOLD:AAG9230
Cyclops|BACZP679-16|Guelph Lake|BOLD:AAG9230
Cyclops|BACZP760-16|Guelph Lake|BOLD:AAG9230
Cyclops|BACZP1382-16|Crawford lake|BOLD:AAG9230
Cyclops|BACZP1383-16|Crawford lake|BOLD:AAG9230
Cyclops|BACZP1384-16|Crawford lake|BOLD:AAG9230
Cyclops|BACZP1391-16|Crawford lake|BOLD:AAG9230
Cyclops|BACZP1393-16|Crawford lake|BOLD:AAG9230
Cyclops|BACZP1394-16|Crawford lake|BOLD:AAG9230
Cyclops|BACZP1445-16|Crawford lake|BOLD:AAG9230
Cyclops|BACZP1446-16|Crawford lake|BOLD:AAG9230
Cyclops|BACZP1214-16|Eel Lake|BOLD:AAG9230
Cyclops|BACZP1561-16|Eel Lake|BOLD:AAG9230
Cyclops|BACZP1269-16|Eel Lake|BOLD:AAG9230
Cyclops|BACZP1216-16|Eel Lake|BOLD:AAG9230
Cyclops|BACZP1213-16|Eel Lake|BOLD:AAG9230
Cyclops|BACZP1212-16|Eel Lake|BOLD:AAG9230
Cyclops|BACZP1210-16|Eel Lake|BOLD:AAG9230
Cyclops|BACZP1208-16|Eel Lake|BOLD:AAG9230
Cyclops|BACZP1579-16|Eel Lake|BOLD:AAG9230
Cyclopoida|BACZP855-16|Guelph Lake|
Cyclops|BACZP1665-16|Fork of the Credit Pond|BOLD:AAG9230
Acanthocyclops americanus|BACZP429-16|Chipila|
Acanthocyclops americanus|BACZP395-16|Atlangatepec|BOLD:AAG9784
Acanthocyclops americanus|BACZP422-16|Chipila|BOLD:AAG9784
Acanthocyclops americanus|BACZP440-16|Chipila|BOLD:AAG9784
Cyclopidae|BACZP1642-16|Fork of the Credit Lake|BOLD:AAV0663
Cyclopidae|BACZP1637-16|Fork of the Credit Lake|BOLD:AAV0663
Cyclopidae|BACZP1639-16|Fork of the Credit Lake|BOLD:AAV0663
Cyclopidae|BACZP1645-16|Fork of the Credit Lake|BOLD:AAV0663
Cyclopidae|BACZP1648-16|Fork of the Credit Lake|BOLD:AAV0663
Cyclopidae|BACZP1662-16|Fork of the Credit Pond|BOLD:AAV0663
Cyclopidae|BACZP1709-16|Fork of the Credit Pond|BOLD:AAV0663
Acanthocyclops vernalis|BACZP330-15|Pond in the other side Guelph Lake|BOLD:ABY0178
Acanthocyclops robustus|BACZP312-15|Pond in the other side Guelph Lake|BOLD:ABY1566
Acanthocyclops|BACZP303-15|Pond in the other side Guelph Lake|BOLD:ACY4027
Acanthocyclops|BACZP367-15|Guelph Lake|BOLD:ACY4027
Cyclopoida|BACZP2271-16|Near Calderitas, pond in a road|BOLD:ADD4521
Cyclopoida|BACZP2270-16|Near Calderitas, pond in a road|BOLD:ADD7680
Cyclopoida|BACZP2274-16|Near Calderitas, pond in a road|BOLD:ADD7680
Cyclopoida|BACZP2272-16|Near Calderitas, pond in a road|BOLD:ADD7680
Cyclopoida|BACZP2275-16|Near Calderitas, pond in a road|BOLD:ADD7680
Cyclopoida|BACZP2277-16|Near Calderitas, pond in a road|BOLD:ADD7680
Cyclopoida|BACZP2278-16|Near Calderitas, pond in a road|BOLD:ADD7680
Cyclopoida|BACZP2279-16|Near Calderitas, pond in a road|BOLD:ADD7680
Thermocyclops inversus|BACZP002-15|Bacalar Lake, Cocalitos Sinkhole|BOLD:AAB4353
Thermocyclops inversus|BACZP162-15|Bacalar Lake, Cocalitos sinkhole, light trap 1|BOLD:A...
Thermocyclops inversus|BACZP164-15|Bacalar Lake, Cocalitos sinkhole, light trap 1|BOLD:A...

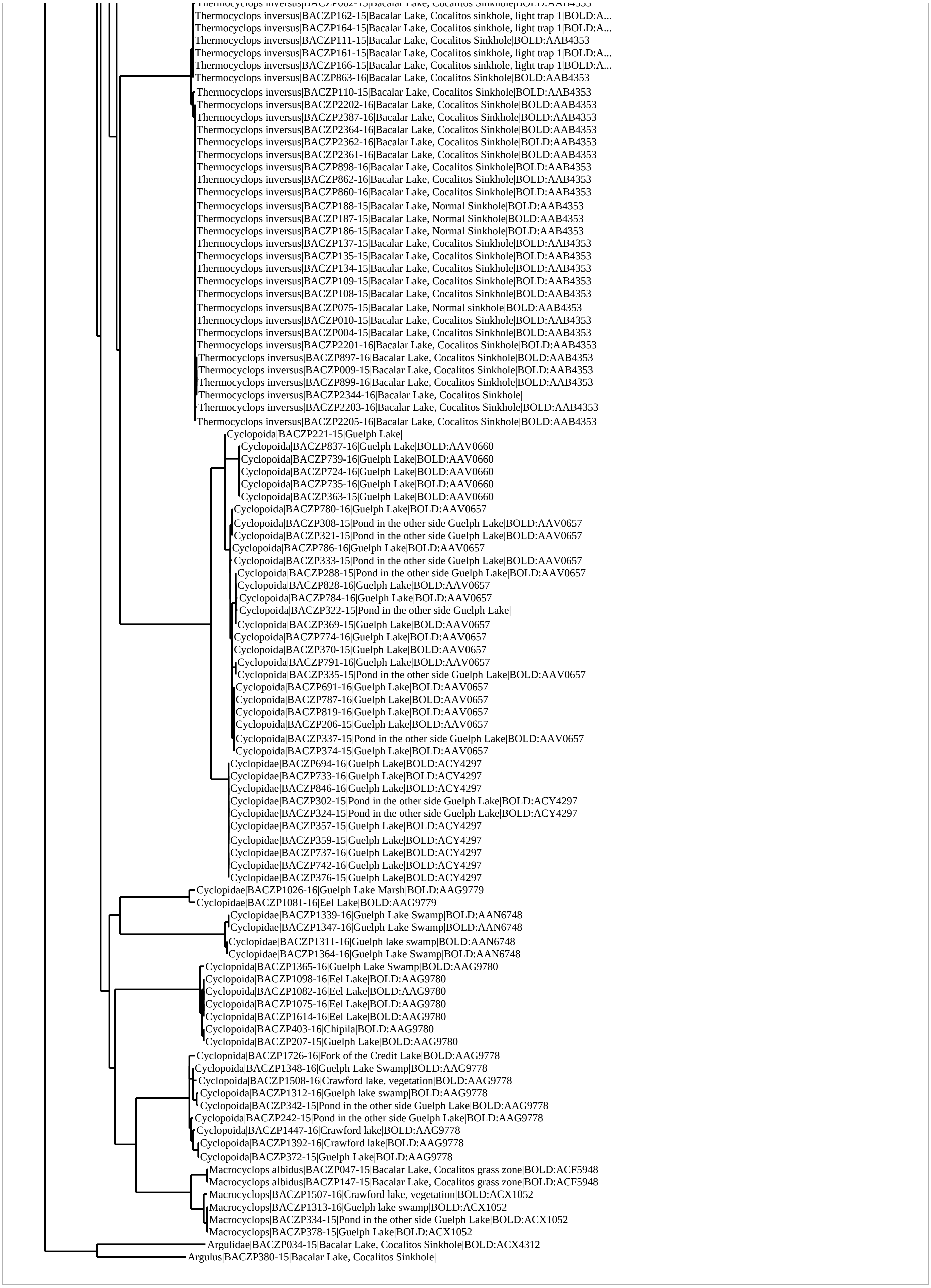
Thermocyclops inversus|BACZP002-15|Bacalar Lake, Cocalitos Sinkhole|BOLD:AAB4353
Thermocyclops inversus|BACZP162-15|Bacalar Lake, Cocalitos sinkhole, light trap 1|BOLD:A...
Thermocyclops inversus|BACZP164-15|Bacalar Lake, Cocalitos sinkhole, light trap 1|BOLD:A...
Thermocyclops inversus|BACZP111-15|Bacalar Lake, Cocalitos Sinkhole|BOLD:AAB4353
Thermocyclops inversus|BACZP161-15|Bacalar Lake, Cocalitos sinkhole, light trap 1|BOLD:A...
Thermocyclops inversus|BACZP166-15|Bacalar Lake, Cocalitos sinkhole, light trap 1|BOLD:A...
Thermocyclops inversus|BACZP863-16|Bacalar Lake, Cocalitos Sinkhole|BOLD:AAB4353
Thermocyclops inversus|BACZP110-15|Bacalar Lake, Cocalitos Sinkhole|BOLD:AAB4353
Thermocyclops inversus|BACZP2202-16|Bacalar Lake, Cocalitos Sinkhole|BOLD:AAB4353
Thermocyclops inversus|BACZP2387-16|Bacalar Lake, Cocalitos Sinkhole|BOLD:AAB4353
Thermocyclops inversus|BACZP2364-16|Bacalar Lake, Cocalitos Sinkhole|BOLD:AAB4353
Thermocyclops inversus|BACZP2362-16|Bacalar Lake, Cocalitos Sinkhole|BOLD:AAB4353
Thermocyclops inversus|BACZP2361-16|Bacalar Lake, Cocalitos Sinkhole|BOLD:AAB4353
Thermocyclops inversus|BACZP898-16|Bacalar Lake, Cocalitos Sinkhole|BOLD:AAB4353
Thermocyclops inversus|BACZP862-16|Bacalar Lake, Cocalitos Sinkhole|BOLD:AAB4353
Thermocyclops inversus|BACZP860-16|Bacalar Lake, Cocalitos Sinkhole|BOLD:AAB4353
Thermocyclops inversus|BACZP188-15|Bacalar Lake, Normal Sinkhole|BOLD:AAB4353
Thermocyclops inversus|BACZP187-15|Bacalar Lake, Normal Sinkhole|BOLD:AAB4353
Thermocyclops inversus|BACZP186-15|Bacalar Lake, Normal Sinkhole|BOLD:AAB4353
Thermocyclops inversus|BACZP137-15|Bacalar Lake, Cocalitos Sinkhole|BOLD:AAB4353
Thermocyclops inversus|BACZP135-15|Bacalar Lake, Cocalitos Sinkhole|BOLD:AAB4353
Thermocyclops inversus|BACZP134-15|Bacalar Lake, Cocalitos Sinkhole|BOLD:AAB4353
Thermocyclops inversus|BACZP109-15|Bacalar Lake, Cocalitos Sinkhole|BOLD:AAB4353
Thermocyclops inversus|BACZP108-15|Bacalar Lake, Cocalitos Sinkhole|BOLD:AAB4353
Thermocyclops inversus|BACZP075-15|Bacalar Lake, Normal sinkhole|BOLD:AAB4353
Thermocyclops inversus|BACZP010-15|Bacalar Lake, Cocalitos Sinkhole|BOLD:AAB4353
Thermocyclops inversus|BACZP004-15|Bacalar Lake, Cocalitos Sinkhole|BOLD:AAB4353
Thermocyclops inversus|BACZP2201-16|Bacalar Lake, Cocalitos Sinkhole|BOLD:AAB4353
Thermocyclops inversus|BACZP897-16|Bacalar Lake, Cocalitos Sinkhole|BOLD:AAB4353
Thermocyclops inversus|BACZP009-15|Bacalar Lake, Cocalitos Sinkhole|BOLD:AAB4353
Thermocyclops inversus|BACZP899-16|Bacalar Lake, Cocalitos Sinkhole|BOLD:AAB4353
Thermocyclops inversus|BACZP2344-16|Bacalar Lake, Cocalitos Sinkhole|
Thermocyclops inversus|BACZP2203-16|Bacalar Lake, Cocalitos Sinkhole|BOLD:AAB4353
Thermocyclops inversus|BACZP2205-16|Bacalar Lake, Cocalitos Sinkhole|BOLD:AAB4353
Cyclopoida|BACZP221-15|Guelph Lake|
Cyclopoida|BACZP837-16|Guelph Lake|BOLD:AAV0660
Cyclopoida|BACZP739-16|Guelph Lake|BOLD:AAV0660
Cyclopoida|BACZP724-16|Guelph Lake|BOLD:AAV0660
Cyclopoida|BACZP735-16|Guelph Lake|BOLD:AAV0660
Cyclopoida|BACZP363-15|Guelph Lake|BOLD:AAV0660
Cyclopoida|BACZP780-16|Guelph Lake|BOLD:AAV0657
Cyclopoida|BACZP308-15|Pond in the other side Guelph Lake|BOLD:AAV0657
Cyclopoida|BACZP321-15|Pond in the other side Guelph Lake|BOLD:AAV0657
Cyclopoida|BACZP786-16|Guelph Lake|BOLD:AAV0657
Cyclopoida|BACZP333-15|Pond in the other side Guelph Lake|BOLD:AAV0657
Cyclopoida|BACZP288-15|Pond in the other side Guelph Lake|BOLD:AAV0657
Cyclopoida|BACZP828-16|Guelph Lake|BOLD:AAV0657
Cyclopoida|BACZP784-16|Guelph Lake|BOLD:AAV0657
Cyclopoida|BACZP322-15|Pond in the other side Guelph Lake|
Cyclopoida|BACZP369-15|Guelph Lake|BOLD:AAV0657
Cyclopoida|BACZP774-16|Guelph Lake|BOLD:AAV0657
Cyclopoida|BACZP370-15|Guelph Lake|BOLD:AAV0657
Cyclopoida|BACZP791-16|Guelph Lake|BOLD:AAV0657
Cyclopoida|BACZP335-15|Pond in the other side Guelph Lake|BOLD:AAV0657
Cyclopoida|BACZP691-16|Guelph Lake|BOLD:AAV0657
Cyclopoida|BACZP787-16|Guelph Lake|BOLD:AAV0657
Cyclopoida|BACZP819-16|Guelph Lake|BOLD:AAV0657
Cyclopoida|BACZP206-15|Guelph Lake|BOLD:AAV0657
Cyclopoida|BACZP337-15|Pond in the other side Guelph Lake|BOLD:AAV0657
Cyclopoida|BACZP374-15|Guelph Lake|BOLD:AAV0657
Cyclopidae|BACZP694-16|Guelph Lake|BOLD:ACY4297
Cyclopidae|BACZP733-16|Guelph Lake|BOLD:ACY4297
Cyclopidae|BACZP846-16|Guelph Lake|BOLD:ACY4297
Cyclopidae|BACZP302-15|Pond in the other side Guelph Lake|BOLD:ACY4297
Cyclopidae|BACZP324-15|Pond in the other side Guelph Lake|BOLD:ACY4297
Cyclopidae|BACZP357-15|Guelph Lake|BOLD:ACY4297
Cyclopidae|BACZP359-15|Guelph Lake|BOLD:ACY4297
Cyclopidae|BACZP737-16|Guelph Lake|BOLD:ACY4297
Cyclopidae|BACZP742-16|Guelph Lake|BOLD:ACY4297
Cyclopidae|BACZP376-15|Guelph Lake|BOLD:ACY4297
Cyclopidae|BACZP1026-16|Guelph Lake Marsh|BOLD:AAG9779
Cyclopidae|BACZP1081-16|Eel Lake|BOLD:AAG9779
Cyclopidae|BACZP1339-16|Guelph Lake Swamp|BOLD:AAN6748
Cyclopidae|BACZP1347-16|Guelph Lake Swamp|BOLD:AAN6748
Cyclopidae|BACZP1311-16|Guelph lake swamp|BOLD:AAN6748
Cyclopidae|BACZP1364-16|Guelph Lake Swamp|BOLD:AAN6748
Cyclopoida|BACZP1365-16|Guelph Lake Swamp|BOLD:AAG9780
Cyclopoida|BACZP1098-16|Eel Lake|BOLD:AAG9780
Cyclopoida|BACZP1082-16|Eel Lake|BOLD:AAG9780
Cyclopoida|BACZP1075-16|Eel Lake|BOLD:AAG9780
Cyclopoida|BACZP1614-16|Eel Lake|BOLD:AAG9780
Cyclopoida|BACZP403-16|Chipila|BOLD:AAG9780
Cyclopoida|BACZP207-15|Guelph Lake|BOLD:AAG9780
Cyclopoida|BACZP1726-16|Fork of the Credit Lake|BOLD:AAG9778
Cyclopoida|BACZP1348-16|Guelph Lake Swamp|BOLD:AAG9778
Cyclopoida|BACZP1508-16|Crawford lake, vegetation|BOLD:AAG9778
Cyclopoida|BACZP1312-16|Guelph lake swamp|BOLD:AAG9778
Cyclopoida|BACZP342-15|Pond in the other side Guelph Lake|BOLD:AAG9778
Cyclopoida|BACZP242-15|Pond in the other side Guelph Lake|BOLD:AAG9778
Cyclopoida|BACZP1447-16|Crawford lake|BOLD:AAG9778
Cyclopoida|BACZP1392-16|Crawford lake|BOLD:AAG9778
Cyclopoida|BACZP372-15|Guelph Lake|BOLD:AAG9778
Macrocyclops albidus|BACZP047-15|Bacalar Lake, Cocalitos grass zone|BOLD:ACF5948
Macrocyclops albidus|BACZP147-15|Bacalar Lake, Cocalitos grass zone|BOLD:ACF5948
Macrocyclops|BACZP1507-16|Crawford lake, vegetation|BOLD:ACX1052
Macrocyclops|BACZP1313-16|Guelph lake swamp|BOLD:ACX1052
Macrocyclops|BACZP334-15|Pond in the other side Guelph Lake|BOLD:ACX1052
Macrocyclops|BACZP378-15|Guelph Lake|BOLD:ACX1052
Argulidae|BACZP034-15|Bacalar Lake, Cocalitos Sinkhole|BOLD:ACX4312
Argulus|BACZP380-15|Bacalar Lake, Cocalitos Sinkhole|

BOLD TaxonID Tree

Actinopterygii

Title	:	Tree Result - Search (196 records)
Date	:	23-Mar-2017
Data Type	:	Nucleotide
Distance Model	:	Kimura 2 Parameter
Marker	:	COI-5P
Colourization	:	[blue]=Stop Codons [red]=Contamination or misidentification

Label	:	Process ID
Label	:	Taxon
Label	:	Country
Label	:	Exact Site
Label	:	Barcode Cluster (BIN)

Sequence Count	:	181
Species count	:	16
Genus count	:	16
Family count	:	13
Unidentified	:	65

BIN Count	:	16

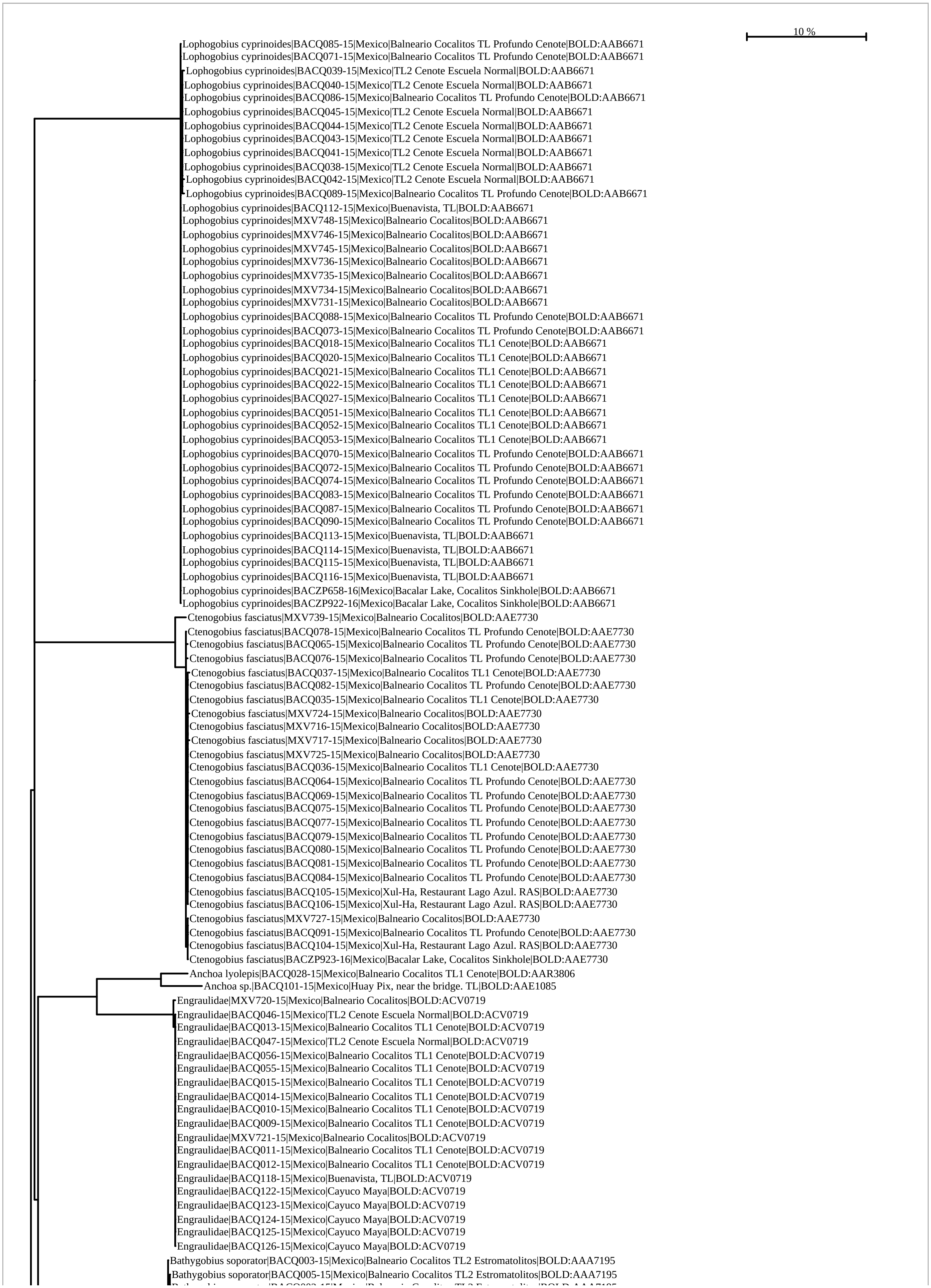
10 %
Lophogobius cyprinoides|BACQ085-15|Mexico|Balneario Cocalitos TL Profundo Cenote|BOLD:AAB6671
Lophogobius cyprinoides|BACQ071-15|Mexico|Balneario Cocalitos TL Profundo Cenote|BOLD:AAB6671
Lophogobius cyprinoides|BACQ039-15|Mexico|TL2 Cenote Escuela Normal|BOLD:AAB6671
Lophogobius cyprinoides|BACQ040-15|Mexico|TL2 Cenote Escuela Normal|BOLD:AAB6671
Lophogobius cyprinoides|BACQ086-15|Mexico|Balneario Cocalitos TL Profundo Cenote|BOLD:AAB6671
Lophogobius cyprinoides|BACQ045-15|Mexico|TL2 Cenote Escuela Normal|BOLD:AAB6671
Lophogobius cyprinoides|BACQ044-15|Mexico|TL2 Cenote Escuela Normal|BOLD:AAB6671
Lophogobius cyprinoides|BACQ043-15|Mexico|TL2 Cenote Escuela Normal|BOLD:AAB6671
Lophogobius cyprinoides|BACQ041-15|Mexico|TL2 Cenote Escuela Normal|BOLD:AAB6671
Lophogobius cyprinoides|BACQ038-15|Mexico|TL2 Cenote Escuela Normal|BOLD:AAB6671
Lophogobius cyprinoides|BACQ042-15|Mexico|TL2 Cenote Escuela Normal|BOLD:AAB6671
Lophogobius cyprinoides|BACQ089-15|Mexico|Balneario Cocalitos TL Profundo Cenote|BOLD:AAB6671
Lophogobius cyprinoides|BACQ112-15|Mexico|Buenavista, TL|BOLD:AAB6671
Lophogobius cyprinoides|MXV748-15|Mexico|Balneario Cocalitos|BOLD:AAB6671
Lophogobius cyprinoides|MXV746-15|Mexico|Balneario Cocalitos|BOLD:AAB6671
Lophogobius cyprinoides|MXV745-15|Mexico|Balneario Cocalitos|BOLD:AAB6671
Lophogobius cyprinoides|MXV736-15|Mexico|Balneario Cocalitos|BOLD:AAB6671
Lophogobius cyprinoides|MXV735-15|Mexico|Balneario Cocalitos|BOLD:AAB6671
Lophogobius cyprinoides|MXV734-15|Mexico|Balneario Cocalitos|BOLD:AAB6671
Lophogobius cyprinoides|MXV731-15|Mexico|Balneario Cocalitos|BOLD:AAB6671
Lophogobius cyprinoides|BACQ088-15|Mexico|Balneario Cocalitos TL Profundo Cenote|BOLD:AAB6671
Lophogobius cyprinoides|BACQ073-15|Mexico|Balneario Cocalitos TL Profundo Cenote|BOLD:AAB6671
Lophogobius cyprinoides|BACQ018-15|Mexico|Balneario Cocalitos TL1 Cenote|BOLD:AAB6671
Lophogobius cyprinoides|BACQ020-15|Mexico|Balneario Cocalitos TL1 Cenote|BOLD:AAB6671
Lophogobius cyprinoides|BACQ021-15|Mexico|Balneario Cocalitos TL1 Cenote|BOLD:AAB6671
Lophogobius cyprinoides|BACQ022-15|Mexico|Balneario Cocalitos TL1 Cenote|BOLD:AAB6671
Lophogobius cyprinoides|BACQ027-15|Mexico|Balneario Cocalitos TL1 Cenote|BOLD:AAB6671
Lophogobius cyprinoides|BACQ051-15|Mexico|Balneario Cocalitos TL1 Cenote|BOLD:AAB6671
Lophogobius cyprinoides|BACQ052-15|Mexico|Balneario Cocalitos TL1 Cenote|BOLD:AAB6671
Lophogobius cyprinoides|BACQ053-15|Mexico|Balneario Cocalitos TL1 Cenote|BOLD:AAB6671
Lophogobius cyprinoides|BACQ070-15|Mexico|Balneario Cocalitos TL Profundo Cenote|BOLD:AAB6671
Lophogobius cyprinoides|BACQ072-15|Mexico|Balneario Cocalitos TL Profundo Cenote|BOLD:AAB6671
Lophogobius cyprinoides|BACQ074-15|Mexico|Balneario Cocalitos TL Profundo Cenote|BOLD:AAB6671
Lophogobius cyprinoides|BACQ083-15|Mexico|Balneario Cocalitos TL Profundo Cenote|BOLD:AAB6671
Lophogobius cyprinoides|BACQ087-15|Mexico|Balneario Cocalitos TL Profundo Cenote|BOLD:AAB6671
Lophogobius cyprinoides|BACQ090-15|Mexico|Balneario Cocalitos TL Profundo Cenote|BOLD:AAB6671
Lophogobius cyprinoides|BACQ113-15|Mexico|Buenavista, TL|BOLD:AAB6671
Lophogobius cyprinoides|BACQ114-15|Mexico|Buenavista, TL|BOLD:AAB6671
Lophogobius cyprinoides|BACQ115-15|Mexico|Buenavista, TL|BOLD:AAB6671
Lophogobius cyprinoides|BACQ116-15|Mexico|Buenavista, TL|BOLD:AAB6671
Lophogobius cyprinoides|BACZP658-16|Mexico|Bacalar Lake, Cocalitos Sinkhole|BOLD:AAB6671
Lophogobius cyprinoides|BACZP922-16|Mexico|Bacalar Lake, Cocalitos Sinkhole|BOLD:AAB6671
Ctenogobius fasciatus|MXV739-15|Mexico|Balneario Cocalitos|BOLD:AAE7730
Ctenogobius fasciatus|BACQ078-15|Mexico|Balneario Cocalitos TL Profundo Cenote|BOLD:AAE7730
Ctenogobius fasciatus|BACQ065-15|Mexico|Balneario Cocalitos TL Profundo Cenote|BOLD:AAE7730
Ctenogobius fasciatus|BACQ076-15|Mexico|Balneario Cocalitos TL Profundo Cenote|BOLD:AAE7730
Ctenogobius fasciatus|BACQ037-15|Mexico|Balneario Cocalitos TL1 Cenote|BOLD:AAE7730
Ctenogobius fasciatus|BACQ082-15|Mexico|Balneario Cocalitos TL Profundo Cenote|BOLD:AAE7730
Ctenogobius fasciatus|BACQ035-15|Mexico|Balneario Cocalitos TL1 Cenote|BOLD:AAE7730
Ctenogobius fasciatus|MXV724-15|Mexico|Balneario Cocalitos|BOLD:AAE7730
Ctenogobius fasciatus|MXV716-15|Mexico|Balneario Cocalitos|BOLD:AAE7730
Ctenogobius fasciatus|MXV717-15|Mexico|Balneario Cocalitos|BOLD:AAE7730
Ctenogobius fasciatus|MXV725-15|Mexico|Balneario Cocalitos|BOLD:AAE7730
Ctenogobius fasciatus|BACQ036-15|Mexico|Balneario Cocalitos TL1 Cenote|BOLD:AAE7730
Ctenogobius fasciatus|BACQ064-15|Mexico|Balneario Cocalitos TL Profundo Cenote|BOLD:AAE7730
Ctenogobius fasciatus|BACQ069-15|Mexico|Balneario Cocalitos TL Profundo Cenote|BOLD:AAE7730
Ctenogobius fasciatus|BACQ075-15|Mexico|Balneario Cocalitos TL Profundo Cenote|BOLD:AAE7730
Ctenogobius fasciatus|BACQ077-15|Mexico|Balneario Cocalitos TL Profundo Cenote|BOLD:AAE7730
Ctenogobius fasciatus|BACQ079-15|Mexico|Balneario Cocalitos TL Profundo Cenote|BOLD:AAE7730
Ctenogobius fasciatus|BACQ080-15|Mexico|Balneario Cocalitos TL Profundo Cenote|BOLD:AAE7730
Ctenogobius fasciatus|BACQ081-15|Mexico|Balneario Cocalitos TL Profundo Cenote|BOLD:AAE7730
Ctenogobius fasciatus|BACQ084-15|Mexico|Balneario Cocalitos TL Profundo Cenote|BOLD:AAE7730
Ctenogobius fasciatus|BACQ105-15|Mexico|Xul-Ha, Restaurant Lago Azul. RAS|BOLD:AAE7730
Ctenogobius fasciatus|BACQ106-15|Mexico|Xul-Ha, Restaurant Lago Azul. RAS|BOLD:AAE7730
Ctenogobius fasciatus|MXV727-15|Mexico|Balneario Cocalitos|BOLD:AAE7730
Ctenogobius fasciatus|BACQ091-15|Mexico|Balneario Cocalitos TL Profundo Cenote|BOLD:AAE7730
Ctenogobius fasciatus|BACQ104-15|Mexico|Xul-Ha, Restaurant Lago Azul. RAS|BOLD:AAE7730
Ctenogobius fasciatus|BACZP923-16|Mexico|Bacalar Lake, Cocalitos Sinkhole|BOLD:AAE7730
Anchoa lyolepis|BACQ028-15|Mexico|Balneario Cocalitos TL1 Cenote|BOLD:AAR3806
Anchoa sp.|BACQ101-15|Mexico|Huay Pix, near the bridge. TL|BOLD:AAE1085
Engraulidae|MXV720-15|Mexico|Balneario Cocalitos|BOLD:ACV0719
Engraulidae|BACQ046-15|Mexico|TL2 Cenote Escuela Normal|BOLD:ACV0719
Engraulidae|BACQ013-15|Mexico|Balneario Cocalitos TL1 Cenote|BOLD:ACV0719
Engraulidae|BACQ047-15|Mexico|TL2 Cenote Escuela Normal|BOLD:ACV0719
Engraulidae|BACQ056-15|Mexico|Balneario Cocalitos TL1 Cenote|BOLD:ACV0719
Engraulidae|BACQ055-15|Mexico|Balneario Cocalitos TL1 Cenote|BOLD:ACV0719
Engraulidae|BACQ015-15|Mexico|Balneario Cocalitos TL1 Cenote|BOLD:ACV0719
Engraulidae|BACQ014-15|Mexico|Balneario Cocalitos TL1 Cenote|BOLD:ACV0719
Engraulidae|BACQ010-15|Mexico|Balneario Cocalitos TL1 Cenote|BOLD:ACV0719
Engraulidae|BACQ009-15|Mexico|Balneario Cocalitos TL1 Cenote|BOLD:ACV0719
Engraulidae|MXV721-15|Mexico|Balneario Cocalitos|BOLD:ACV0719
Engraulidae|BACQ011-15|Mexico|Balneario Cocalitos TL1 Cenote|BOLD:ACV0719
Engraulidae|BACQ012-15|Mexico|Balneario Cocalitos TL1 Cenote|BOLD:ACV0719
Engraulidae|BACQ118-15|Mexico|Buenavista, TL|BOLD:ACV0719
Engraulidae|BACQ122-15|Mexico|Cayuco Maya|BOLD:ACV0719
Engraulidae|BACQ123-15|Mexico|Cayuco Maya|BOLD:ACV0719
Engraulidae|BACQ124-15|Mexico|Cayuco Maya|BOLD:ACV0719
Engraulidae|BACQ125-15|Mexico|Cayuco Maya|BOLD:ACV0719
Engraulidae|BACQ126-15|Mexico|Cayuco Maya|BOLD:ACV0719
Bathygobius soporator|BACQ003-15|Mexico|Balneario Cocalitos TL2 Estromatolitos|BOLD:AAA7195
Bathygobius soporator|BACQ005-15|Mexico|Balneario Cocalitos TL2 Estromatolitos|BOLD:AAA7195

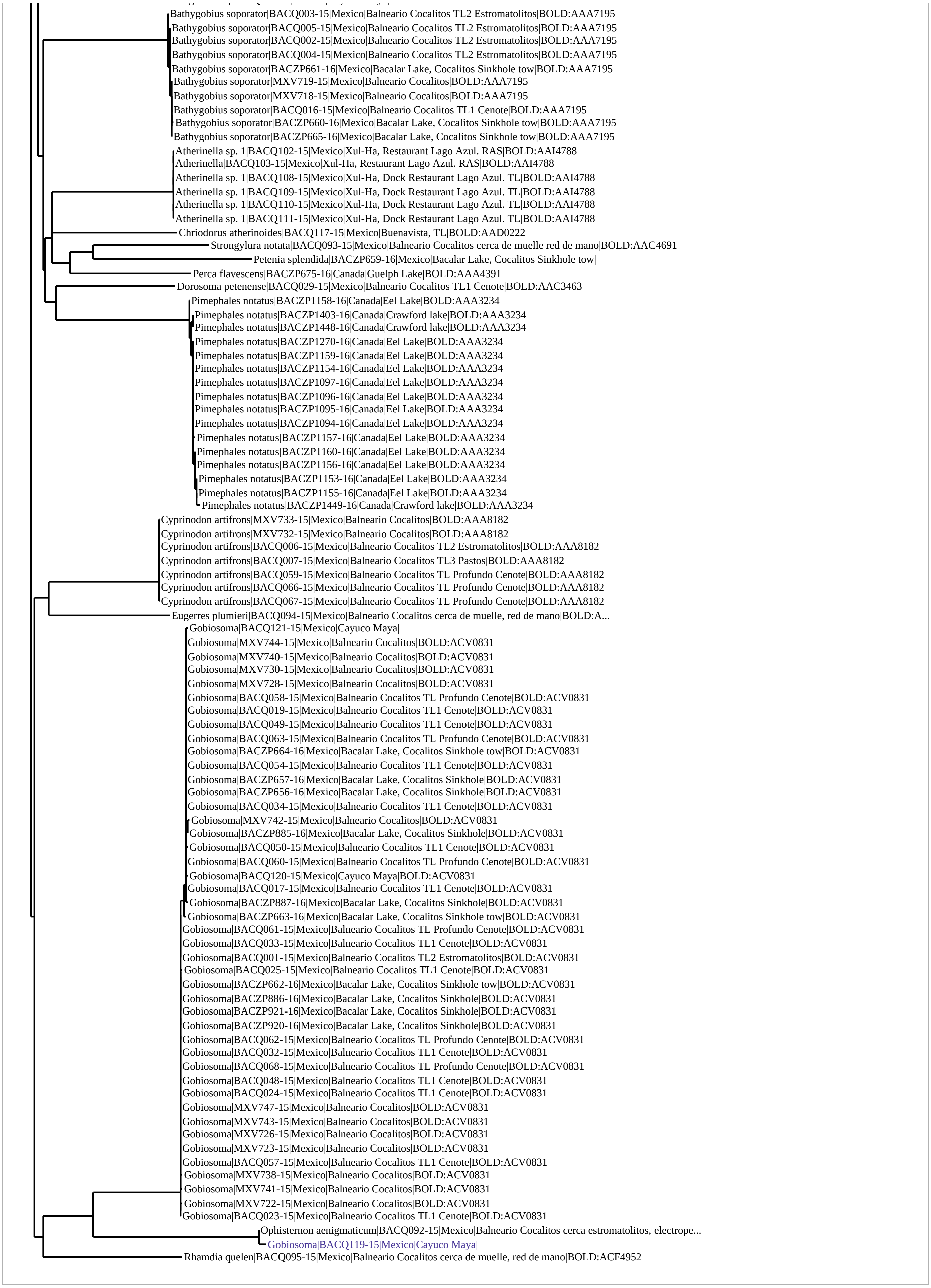
Bathygobius soporator|BACQ003-15|Mexico|Balneario Cocalitos TL2 Estromatolitos|BOLD:AAA7195
Bathygobius soporator|BACQ005-15|Mexico|Balneario Cocalitos TL2 Estromatolitos|BOLD:AAA7195
Bathygobius soporator|BACQ002-15|Mexico|Balneario Cocalitos TL2 Estromatolitos|BOLD:AAA7195
Bathygobius soporator|BACQ004-15|Mexico|Balneario Cocalitos TL2 Estromatolitos|BOLD:AAA7195
Bathygobius soporator|BACZP661-16|Mexico|Bacalar Lake, Cocalitos Sinkhole tow|BOLD:AAA7195
Bathygobius soporator|MXV719-15|Mexico|Balneario Cocalitos|BOLD:AAA7195
Bathygobius soporator|MXV718-15|Mexico|Balneario Cocalitos|BOLD:AAA7195
Bathygobius soporator|BACQ016-15|Mexico|Balneario Cocalitos TL1 Cenote|BOLD:AAA7195
Bathygobius soporator|BACZP660-16|Mexico|Bacalar Lake, Cocalitos Sinkhole tow|BOLD:AAA7195
Bathygobius soporator|BACZP665-16|Mexico|Bacalar Lake, Cocalitos Sinkhole tow|BOLD:AAA7195
Atherinella sp. 1|BACQ102-15|Mexico|Xul-Ha, Restaurant Lago Azul. RAS|BOLD:AAI4788
Atherinella|BACQ103-15|Mexico|Xul-Ha, Restaurant Lago Azul. RAS|BOLD:AAI4788
Atherinella sp. 1|BACQ108-15|Mexico|Xul-Ha, Dock Restaurant Lago Azul. TL|BOLD:AAI4788
Atherinella sp. 1|BACQ109-15|Mexico|Xul-Ha, Dock Restaurant Lago Azul. TL|BOLD:AAI4788
Atherinella sp. 1|BACQ110-15|Mexico|Xul-Ha, Dock Restaurant Lago Azul. TL|BOLD:AAI4788
Atherinella sp. 1|BACQ111-15|Mexico|Xul-Ha, Dock Restaurant Lago Azul. TL|BOLD:AAI4788
Chriodorus atherinoides|BACQ117-15|Mexico|Buenavista, TL|BOLD:AAD0222
Strongylura notata|BACQ093-15|Mexico|Balneario Cocalitos cerca de muelle red de mano|BOLD:AAC4691
Petenia splendida|BACZP659-16|Mexico|Bacalar Lake, Cocalitos Sinkhole tow|
Perca flavescens|BACZP675-16|Canada|Guelph Lake|BOLD:AAA4391
Dorosoma petenense|BACQ029-15|Mexico|Balneario Cocalitos TL1 Cenote|BOLD:AAC3463
Pimephales notatus|BACZP1158-16|Canada|Eel Lake|BOLD:AAA3234
Pimephales notatus|BACZP1403-16|Canada|Crawford lake|BOLD:AAA3234
Pimephales notatus|BACZP1448-16|Canada|Crawford lake|BOLD:AAA3234
Pimephales notatus|BACZP1270-16|Canada|Eel Lake|BOLD:AAA3234
Pimephales notatus|BACZP1159-16|Canada|Eel Lake|BOLD:AAA3234
Pimephales notatus|BACZP1154-16|Canada|Eel Lake|BOLD:AAA3234
Pimephales notatus|BACZP1097-16|Canada|Eel Lake|BOLD:AAA3234
Pimephales notatus|BACZP1096-16|Canada|Eel Lake|BOLD:AAA3234
Pimephales notatus|BACZP1095-16|Canada|Eel Lake|BOLD:AAA3234
Pimephales notatus|BACZP1094-16|Canada|Eel Lake|BOLD:AAA3234
Pimephales notatus|BACZP1157-16|Canada|Eel Lake|BOLD:AAA3234
Pimephales notatus|BACZP1160-16|Canada|Eel Lake|BOLD:AAA3234
Pimephales notatus|BACZP1156-16|Canada|Eel Lake|BOLD:AAA3234
Pimephales notatus|BACZP1153-16|Canada|Eel Lake|BOLD:AAA3234
Pimephales notatus|BACZP1155-16|Canada|Eel Lake|BOLD:AAA3234
Pimephales notatus|BACZP1449-16|Canada|Crawford lake|BOLD:AAA3234
Cyprinodon artifrons|MXV733-15|Mexico|Balneario Cocalitos|BOLD:AAA8182
Cyprinodon artifrons|MXV732-15|Mexico|Balneario Cocalitos|BOLD:AAA8182
Cyprinodon artifrons|BACQ006-15|Mexico|Balneario Cocalitos TL2 Estromatolitos|BOLD:AAA8182
Cyprinodon artifrons|BACQ007-15|Mexico|Balneario Cocalitos TL3 Pastos|BOLD:AAA8182
Cyprinodon artifrons|BACQ059-15|Mexico|Balneario Cocalitos TL Profundo Cenote|BOLD:AAA8182
Cyprinodon artifrons|BACQ066-15|Mexico|Balneario Cocalitos TL Profundo Cenote|BOLD:AAA8182
Cyprinodon artifrons|BACQ067-15|Mexico|Balneario Cocalitos TL Profundo Cenote|BOLD:AAA8182
Eugerres plumieri|BACQ094-15|Mexico|Balneario Cocalitos cerca de muelle, red de mano|BOLD:A...
Gobiosoma|BACQ121-15|Mexico|Cayuco Maya|
Gobiosoma|MXV744-15|Mexico|Balneario Cocalitos|BOLD:ACV0831
Gobiosoma|MXV740-15|Mexico|Balneario Cocalitos|BOLD:ACV0831
Gobiosoma|MXV730-15|Mexico|Balneario Cocalitos|BOLD:ACV0831
Gobiosoma|MXV728-15|Mexico|Balneario Cocalitos|BOLD:ACV0831
Gobiosoma|BACQ058-15|Mexico|Balneario Cocalitos TL Profundo Cenote|BOLD:ACV0831
Gobiosoma|BACQ019-15|Mexico|Balneario Cocalitos TL1 Cenote|BOLD:ACV0831
Gobiosoma|BACQ049-15|Mexico|Balneario Cocalitos TL1 Cenote|BOLD:ACV0831
Gobiosoma|BACQ063-15|Mexico|Balneario Cocalitos TL Profundo Cenote|BOLD:ACV0831
Gobiosoma|BACZP664-16|Mexico|Bacalar Lake, Cocalitos Sinkhole tow|BOLD:ACV0831
Gobiosoma|BACQ054-15|Mexico|Balneario Cocalitos TL1 Cenote|BOLD:ACV0831
Gobiosoma|BACZP657-16|Mexico|Bacalar Lake, Cocalitos Sinkhole|BOLD:ACV0831
Gobiosoma|BACZP656-16|Mexico|Bacalar Lake, Cocalitos Sinkhole|BOLD:ACV0831
Gobiosoma|BACQ034-15|Mexico|Balneario Cocalitos TL1 Cenote|BOLD:ACV0831
Gobiosoma|MXV742-15|Mexico|Balneario Cocalitos|BOLD:ACV0831
Gobiosoma|BACZP885-16|Mexico|Bacalar Lake, Cocalitos Sinkhole|BOLD:ACV0831
Gobiosoma|BACQ050-15|Mexico|Balneario Cocalitos TL1 Cenote|BOLD:ACV0831
Gobiosoma|BACQ060-15|Mexico|Balneario Cocalitos TL Profundo Cenote|BOLD:ACV0831
Gobiosoma|BACQ120-15|Mexico|Cayuco Maya|BOLD:ACV0831
Gobiosoma|BACQ017-15|Mexico|Balneario Cocalitos TL1 Cenote|BOLD:ACV0831
Gobiosoma|BACZP887-16|Mexico|Bacalar Lake, Cocalitos Sinkhole|BOLD:ACV0831
Gobiosoma|BACZP663-16|Mexico|Bacalar Lake, Cocalitos Sinkhole tow|BOLD:ACV0831
Gobiosoma|BACQ061-15|Mexico|Balneario Cocalitos TL Profundo Cenote|BOLD:ACV0831
Gobiosoma|BACQ033-15|Mexico|Balneario Cocalitos TL1 Cenote|BOLD:ACV0831
Gobiosoma|BACQ001-15|Mexico|Balneario Cocalitos TL2 Estromatolitos|BOLD:ACV0831
Gobiosoma|BACQ025-15|Mexico|Balneario Cocalitos TL1 Cenote|BOLD:ACV0831
Gobiosoma|BACZP662-16|Mexico|Bacalar Lake, Cocalitos Sinkhole tow|BOLD:ACV0831
Gobiosoma|BACZP886-16|Mexico|Bacalar Lake, Cocalitos Sinkhole|BOLD:ACV0831
Gobiosoma|BACZP921-16|Mexico|Bacalar Lake, Cocalitos Sinkhole|BOLD:ACV0831
Gobiosoma|BACZP920-16|Mexico|Bacalar Lake, Cocalitos Sinkhole|BOLD:ACV0831
Gobiosoma|BACQ062-15|Mexico|Balneario Cocalitos TL Profundo Cenote|BOLD:ACV0831
Gobiosoma|BACQ032-15|Mexico|Balneario Cocalitos TL1 Cenote|BOLD:ACV0831
Gobiosoma|BACQ068-15|Mexico|Balneario Cocalitos TL Profundo Cenote|BOLD:ACV0831
Gobiosoma|BACQ048-15|Mexico|Balneario Cocalitos TL1 Cenote|BOLD:ACV0831
Gobiosoma|BACQ024-15|Mexico|Balneario Cocalitos TL1 Cenote|BOLD:ACV0831
Gobiosoma|MXV747-15|Mexico|Balneario Cocalitos|BOLD:ACV0831
Gobiosoma|MXV743-15|Mexico|Balneario Cocalitos|BOLD:ACV0831
Gobiosoma|MXV726-15|Mexico|Balneario Cocalitos|BOLD:ACV0831
Gobiosoma|MXV723-15|Mexico|Balneario Cocalitos|BOLD:ACV0831
Gobiosoma|BACQ057-15|Mexico|Balneario Cocalitos TL1 Cenote|BOLD:ACV0831
Gobiosoma|MXV738-15|Mexico|Balneario Cocalitos|BOLD:ACV0831
Gobiosoma|MXV741-15|Mexico|Balneario Cocalitos|BOLD:ACV0831
Gobiosoma|MXV722-15|Mexico|Balneario Cocalitos|BOLD:ACV0831
Gobiosoma|BACQ023-15|Mexico|Balneario Cocalitos TL1 Cenote|BOLD:ACV0831
Ophisternon aenigmaticum|BACQ092-15|Mexico|Balneario Cocalitos cerca estromatolitos, electrope...
Gobiosoma|BACQ119-15|Mexico|Cayuco Maya|
Rhamdia quelen|BACQ095-15|Mexico|Balneario Cocalitos cerca de muelle, red de mano|BOLD:ACF4952
